# A Theoretical Kinetic Study on Concerted Elimination Reaction Class of Peroxyl-hydroperoxyl-alkyl Radicals (•OOQOOH) in Normal-alkyl Cyclohexanes

**DOI:** 10.3390/molecules28186612

**Published:** 2023-09-14

**Authors:** Xiaoxia Yao, Jilong Zhang, Yifei Zhu

**Affiliations:** 1 National Key Lab of Aerospace Power System and Plasma Technology, Air Force Engineering University, Xi’an 710038, China; yaoxxkgd@163.com; 2 Institute of Theoretical Chemistry, College of Chemistry, Jilin University, Changchun 130023, China; 3 School of Electrical Engineering, Xi’an Jiaotong University, Xi’an 710049, China

**Keywords:** peroxyl-hydroperoxyl alkyl radicals, concerted elimination reaction, kinetic parameters, reaction rate rules

## Abstract

The concerted elimination reaction class of peroxyl-hydroperoxyl alkyl radicals (•OOQOOH) plays a crucial role in the low-temperature combustion of normal-alkyl cyclohexanes. The generation of the relatively unreactive HO_2_ radicals in this reaction is one of the factors leading to the negative temperature coefficient (NTC) behavior, which hinders the low-temperature oxidation of normal-alkyl cyclohexanes. In this study, 44 reactions are selected and divided into 4 different subclasses according to the nature of the carbon atom where the H atom is eliminated and the reaction center position. Utilizing the CBS-QB3 method, we compute the energy barriers for the concerted elimination reactions of peroxyl-hydroperoxyl alkyl radicals. Following this, we assess both the high-pressure limit and pressure-dependent rate constants for all reactions by applying TST and RRKM/ME theory. These calculations allow for the development of rate rules, which come to fruition through an averaging process involving the rate constants of representative reactions within each subclass. Our work provides accurate rate constants and rate rules for this reaction class, which can aid in constructing more accurate combustion mechanisms for normal-alkyl cyclohexanes.

## 1. Introduction

The energy required for aircraft flight is provided by the combustion process of fuel in the engine combustion chamber. Currently, the main fuel for aviation engines is hydrocarbons, which mainly consist of alkanes, alkenes, cycloalkanes, and aromatics [1,2]. However, while fuel provides energy for aircraft flight, the emissions of carbon particles, nitrogen oxides, and gaseous byproducts from fuel combustion in the combustion chamber severely affects air quality and human health [3,4,5,6]. Therefore, to improve the performance and efficiency of existing engine combustion chambers and reduce the production of harmful pollutants during combustion, extensive research has been conducted on the oxidation process of hydrocarbon fuels under various operating conditions. Understanding the fundamental mechanisms of combustion and replicating these processes through detailed kinetic models are powerful tools for improving fuel quality and optimizing combustion device design [7]. However, due to the complex composition of hydrocarbon fuels, which contain hundreds or thousands of chemical components, including all of these components in a kinetic model would make the model difficult to establish and computationally expensive. To facilitate the simulation of real fuel combustion, a common approach is to select representative surrogate components and construct detailed combustion reaction mechanisms for these surrogate components to effectively replicate the important combustion characteristics of the target fuel. 

Cycloalkanes, as an important hydrocarbon compound in real transportation fuels [8,9,10,11], typically make up about 35% of conventional diesel, around 20% of jet fuel, and roughly 10% of gasoline. Cycloalkanes are also widely used in the surrogate fuel formulations [12,13,14,15,16,17]. Compared to n-alkanes and alkenes, cycloalkanes exhibit unique combustion characteristics, especially in terms of their low-temperature chemical properties. The negative temperature coefficient (NTC) behavior of cycloalkanes falls between those of alkenes and normal alkanes: it is more pronounced than that of alkenes, but less than that of n-alkanes. During the combustion process, cycloalkanes undergo dehydrogenation reactions to produce aromatic hydrocarbons, which are considered the starting points for soot growth [18]. Thus, the high concentration of cycloalkanes can increase the soot emissions in the actual fuel combustion process. Therefore, considering the significant correlation between cyclic hydrocarbons and the combustion process, it is necessary to conduct detailed research on cycloalkanes.

**Scheme 1:**
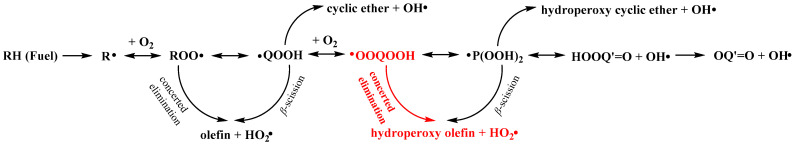
The kinetic scheme for cycloalkane low-temperature oxidation (The class of reaction studied in this study is marked in red).

Similar to alkanes, the simplified kinetic scheme of low-temperature oxidation of cycloalkanes is shown in Scheme 1 [19,20]. 

At low temperature, alkane consumption is initiated by H-atom abstraction from the alkanes, which produces alkyl radicals (R•). These R• can add to the first molecular oxygen to form alkyl peroxy radical (ROO•), which can occur via intermolecular hydrogen migration to form hydroperoxyalkyl radical (•QOOH). •QOOH can add to the second molecular oxygen to produce hydroperoxyl-alkyl-peroxyl radical (•OOQOOH), and it is now widely accepted that the reactions starting from •OOQOOH are very important for the combustion modeling of hydrocarbons at low temperature. The first and most important reaction class for the consumption of •OOQOOH is the internal transfer of a hydrogen atom to form a dihydroperoxy alkyl radical (•P(OOH)_2_). The second reaction pathway for the consumption of •OOQOOH is the concerted elimination of HO_2_• to produce hydroperoxy olefin, which is responsible for a chain propagation since only one radical is produced. This reaction pathway of the concerted elimination from hydroperoxyl-alkyl-peroxyl radicals (•OOQOOH) is also responsible for the majority of relatively unreactive HO_2_• and high-molecular-weight alkenes formed in the low-temperature regions, and it shows the characteristic negative temperature coefficient (NTC) behavior [21,22].

The reliability of combustion dynamic simulations depends on the rationality of the reaction network in the detailed mechanism and the accuracy of the relevant thermodynamic parameters and transport parameters [23]. A detailed hydrocarbon fuel mechanism typically consists of two parts: a core mechanism composed of smaller species (C_0_–C_4_, excluding species with more than four carbon atoms) that have good generality, and an extended mechanism for larger molecules (C_5_ and above) that is automatically generated by mechanism generation programs based on reaction class rules. This hierarchical strategy ensures the rationality of combustion mechanism construction while greatly simplifying the mechanism. In the development of mechanisms, two aspects are of crucial importance: analyzing possible reaction pathways to maintain the completeness of reactions, and ensuring the accuracy of relevant thermodynamic parameters. In the construction of detailed mechanisms, due to the large volume of the mechanism, it is not feasible to calculate the exact rate constants for each reaction class using electronic structure methods. Therefore, reliable rate constants for each reaction class can be obtained based on rate rule estimation methods, without the need for time-consuming ab initio calculations [24,25,26,27]. This method is commonly used in the automatic construction of large molecule combustion mechanisms. To our knowledge, there have been no specific studies on the kinetics of the concerted elimination of •OOQOOH for cycloalkanes, and there are no reported rate rules for this reaction class, which play a crucial role in building the low and intermediate temperature models of cycloalkanes. In modeling studies of cycloalkane combustion, some kinetic parameters are approximated from similar reaction classes in alkanes when specific parameters for cycloalkanes are not available. This approximation can lead to significant errors in the modeling process.

Therefore, in this study, we systematically investigate the concerted elimination reaction class of •OOQOOH in order to fill in the research gap in this area. We determine the high-pressure-limit rate constants and the pressure-dependent rate constants of these reactions through theoretical calculations and establish the corresponding high-pressure-limit rate rules and the pressure-dependent rate rules. In sum, this study will establish accurate rate rules for the concerted elimination reaction class of •OOQOOH in normal-alkyl cyclohexanes, providing a theoretical basis for constructing more comprehensive normal-alkyl cyclohexane low-temperature combustion mechanisms.

## 2. Results and Discussion

**Scheme 2:**
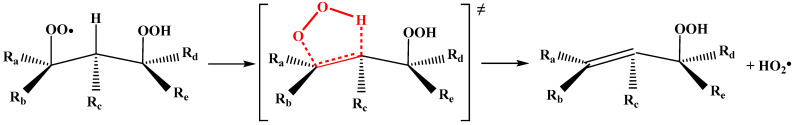
The reaction process of the concerted elimination reaction class of •OOQOOH.

**Scheme 3:**
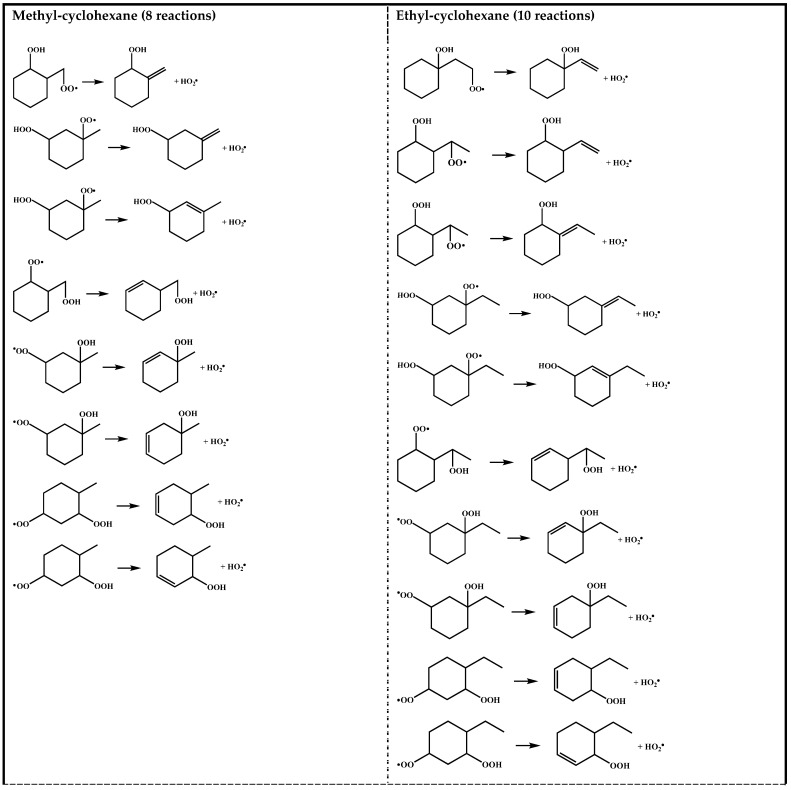

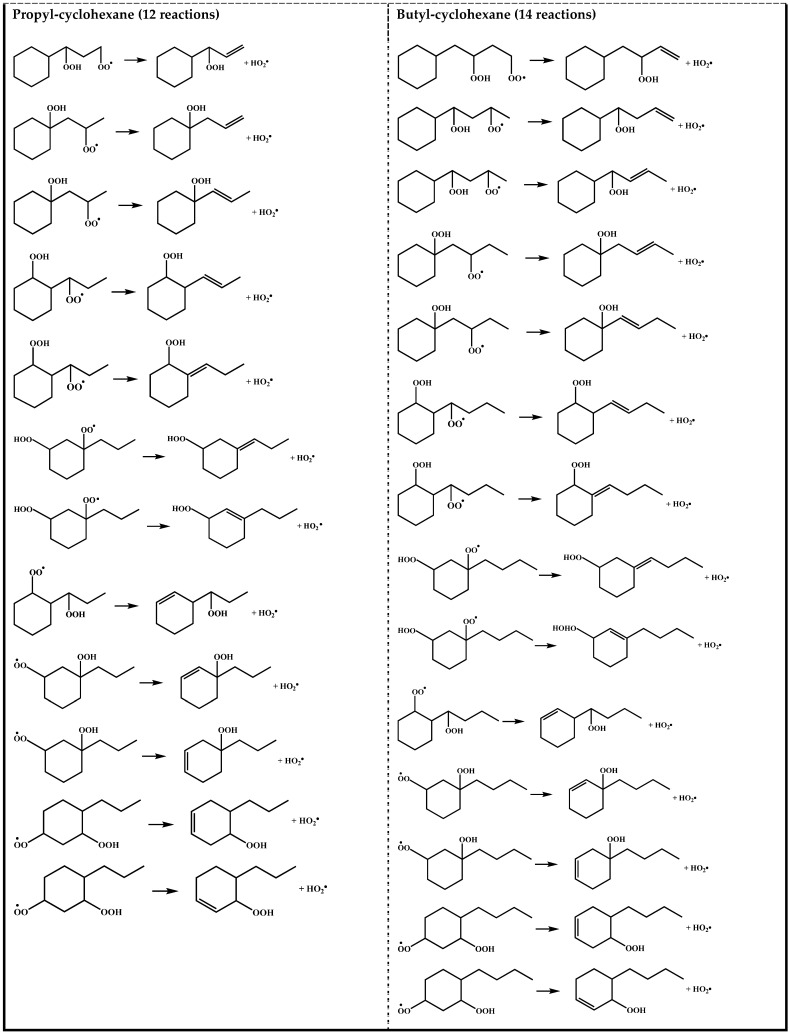
Summary scheme of the concerted elimination reaction class of •OOQOOH.

In our previous study [25], •QOOH formed from 1,5 H-migration of ROO• in normal-alkyl cyclohexanes proved to be the dominant intermediate undergoing further O_2_ addition for the studied H-migration reaction pathways; thus, in this study, the configurations of •OOQOOH corresponding to the 1,5 H-migration of ROO• were selected as the starting reactant configurations for concerted elimination reactions to be studied. The reaction process of the concerted elimination reactions of •OOQOOH is shown in Scheme 2, and the reaction process and the reaction center are marked in red. In Scheme 2, Ra, Rb, Rc, and Rd represent hydrogen or alkyl substituents. A set of 44 representative reactions (methyl, ethyl, n-propyl and n-butyl cyclohexane) was selected for this study, and the studied reactions are shown in Scheme 3.

### 2.1. Conformational Analysis 

**Scheme 4:**
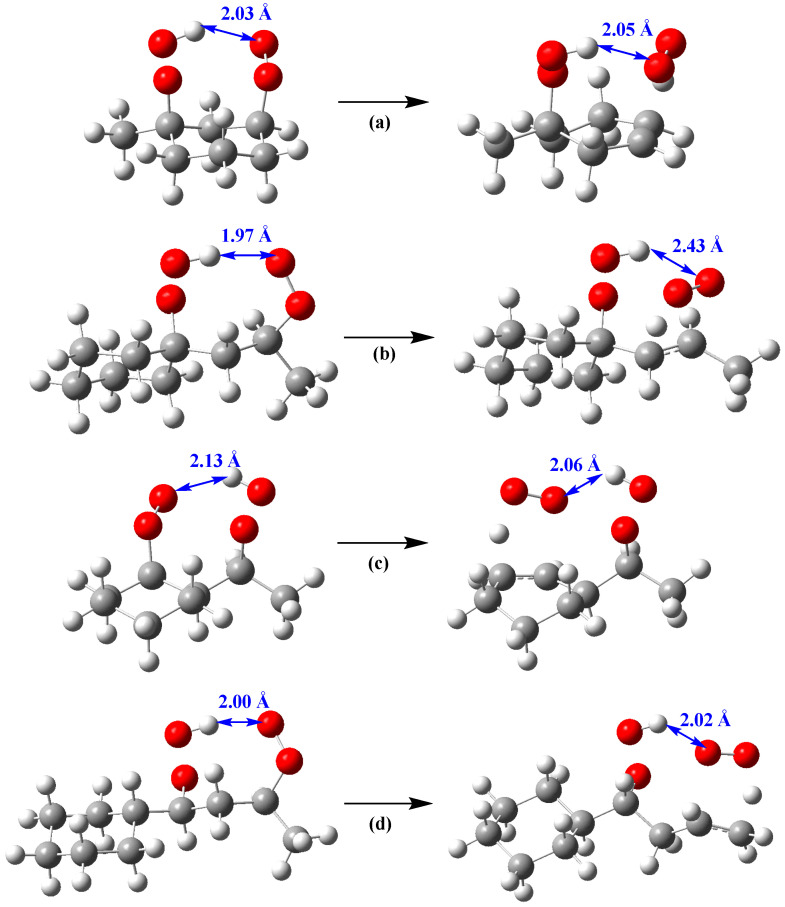
Hydrogen bonds in reactants (**left**) and transition state (**right**): (**a**) both -OOH and -OO moieties are located on the ring; (**b**) one -OOH moiety is located on the ring and one -OO moiety is located on the alkyl side chain; (**c**) one -OO moiety is located on the ring and one -OOH moiety is located on the alkyl side chain; (**d**) both -OOH and -OO moieties are located on the alkyl side chain (The distance of hydrogen bonds are indicated by the blue arrows).

In this study system, since both the reactants and transition states contain -OOH moieties and -OO moieties, it is necessary to consider the effects of intramolecular hydrogen bonds between these two moieties on the conformations. In this study, the effects of intramolecular hydrogen bonds between the hydrogen atoms in one -OOH moiety and the oxygen atoms in one -OO moiety on the conformations of species are considered. The effects of hydrogen bonds can lead to the formation of ring structures involving several conformations, which are described by ring-to-ring conversion. In this study, there are three different scenarios for the presence of the -OOH and -OO moieties in a cycloalkane, as follows: (1) both -OOH and -OO moieties are located on the ring, as in Scheme 4a; (2) one -OOH moiety is located on the ring and one -OO moiety is located on the alkyl side chain, as in Scheme 4b; (3) one -OO moiety is located on the ring and one -OOH moiety is located on the alkyl side chain, as in Scheme 4c; (4) both -OOH and -OO moieties are located on the alkyl side chain, as in Scheme 4d.

### 2.2. Energy Barrier

The reaction barriers for all the concerted elimination reactions of •OO11QOOH are calculated in this study at the CBS-QB3 level and are listed in Table 1 and Table 2. In this research system, the concerted elimination reactions of •OOQOOH are divided into four subclasses according to the following rules: (1) the type of carbon atom in which the H atom is eliminated (a carbon atom that connects with three hydrogen atoms is defined as p section, a carbon atom that connects with two hydrogen atoms is defined as s section, and one hydrogen atom that connects with one hydrogen atom is defined as t section), and (2) the locations of the reaction center (on the alkyl side chain or on the ring). These four subclasses are named: Eli-p-SC, Eli-s-SC, Eli-t-SC, and Eli-s-RI. To evaluate whether the classification criteria of subclasses are reasonable, we give the maximum barrier deviation (the difference between the maximum barrier and the minimum barrier in a subclass) in each subclass to evaluate the reasonableness of our subclasses. From Table 1 and Table 2, it can be seen that the maximum absolute deviation of the reaction barriers in each subclass is within the range of ~3.5 kcal mol^−1^, indicating that the rate rules constructed in this study are reasonable. As can be seen from Table 1, the average energy barriers change as follows: when the positions of the reaction centers are all located on the alkyl side chain of the cyclohexanes, the average energy barrier at the p site is higher than that at the s site, and the s site is higher than that at the t site. In addition, it can be seen from Table 1 and Table 2 that for the four subclasses of the concerted elimination reactions of •OOQOOH, when the reaction center occurs on the ring, the energy barrier of the subclass is higher than those of the reaction centers occurring on the alkyl side chain.

**Figure 1:**
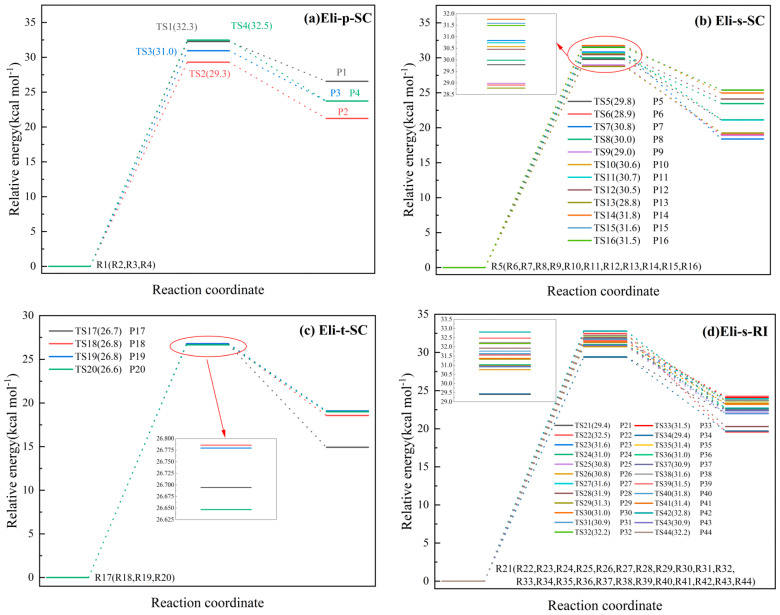
Energy level diagrams of the reactants, transition states and products of reactions in each subclass (reactants just use the same starting point, not the same reactants).

In order to compare the difference between different reactions, energy level diagrams of the reactants, transition states and products of reactions in each subclass are shown in Figure 1. As can be seen from Figure 1, there are certain differences in the energy barriers of different reactions in the same subclass. At the same time, when comparing Figure 1a–d, it can be seen that the energy barriers of different reactions in different subclasses are more obvious. The comparison of the positive and reverse reaction activation energies of each reaction in the concerted elimination class can also be seen in Figure 1. In each reaction, the activation energy of the positive reaction is greater than the activation energy of the reverse reaction, indicating that the concerted elimination reactions studied in this work belong to endothermic reactions.

### 2.3. Energy Barrier Comparison with Similar Reactions in Alkanes

Sun et al. [20] used the isodesmic reaction method to calculate the rate constants for a representative set of 45 reactions in the class of the concerted elimination reactions from •OOQOOH, and some representative reactions are selected to be compared with the concerted elimination reactions with similar reaction types in this study, to explore the influence of the presence of rings in cyclohexane on the reaction energy barrier heights. The energy barrier of the reaction R5 (
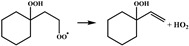
) calculated in this study is 29.8 kcal mol^−1^, which is much lower than the energy barrier of 33.7 kcal mol^−1^ for the analogous reaction (•OO(CH_2_)_3_OOH → CH_2_=CHCH_2_OOH + HO_2_•) calculated by Sun et al. The energy barrier of the reaction R8 (
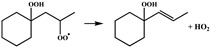
) calculated in this work is 30.0 kcal mol^−1^, which is lower than the energy barrier of 32.9 kcal mol^−1^ for the analogous reaction (CH_3_CH (OO•)(CH_2_)_2_OOH → CH_3_CH=CHCH_2_OOH + HO_2_•) calculated by Sun et al. Goldsmith et al. [28] studied the kinetics of the C_3_H_7_O_2_ and C_3_H_7_O_4_ potential energy surfaces in high–level ab initio calculations, which also included the concerted HO_2_ elimination reactions of •OOCH_2_CH_2_CH_2_OOH and •OOCH_2_CH (OOH)CH_3_. The energy barrier of the reaction R5 (
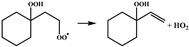
) calculated in this work is 29.8 kcal mol^−1^, which is lower than the energy barrier of 30.3 kcal mol^−1^ for the analogous reaction (•OOCH_2_CH_2_CH_2_OOH → CH_2_=CHCH_2_OOH + HO_2_•) calculated by Goldsmith et al. The results of comparisons show that the energy barriers for the reactions in different types of the concerted elimination of •OOQOOH calculated in this study are obviously different from the analogous reactions in non-cyclic alkanes, indicating that the presence of rings in cyclohexane can influence the heights of the energy barriers.

### 2.4. High-Pressure-Limit Rate Constants and Rate Rules

According to the traditional transition state theory, this study calculates the high-pressure-limit rate constants for concerted elimination of •OOQOOH from 500 K to 1500 K. The simple rate rule method is employed, which involves taking the average of the high-pressure-limit rate constants for all reactions within each subclass to determine the corresponding high-pressure-limit rate rules. Table 3 and Table 4 provide all the rate constants and rate rules, represented by three parameters (*A*, *n*, *E*), obtained by fitting the modified Arrhenius equation in this study. To facilitate comparisons between different reactions or rate rules, the high-pressure-limit rate constants at 800 K are also included in the tables. Additionally, the ratios of the rate constants for each reaction within a subclass to the average rate constant are presented in the tables, to visually illustrate the deviation of the rate constants within each subclass. Moreover, to assess the uncertainty of each subclass rate rule, the ratio of the maximum and minimum high-pressure-limit rate constants within each subclass is defined as the uncertainty factor for that subclass, and the results are also listed in the tables. From Table 3 and Table 4, it can be observed that the ratios between the rate constants for reactions and the average rate constant within each subclass, as well as the uncertainty factors for each subclass, are within 1~2 orders of magnitude. This indicates that the high-pressure-limit rate rules constructed for the concerted elimination reactions of •OOQOOH are reasonable and acceptable.

#### 2.4.1. Comparison of the High-Pressure-Limit Rate Rules between Different Subclasses

**Figure 2:**
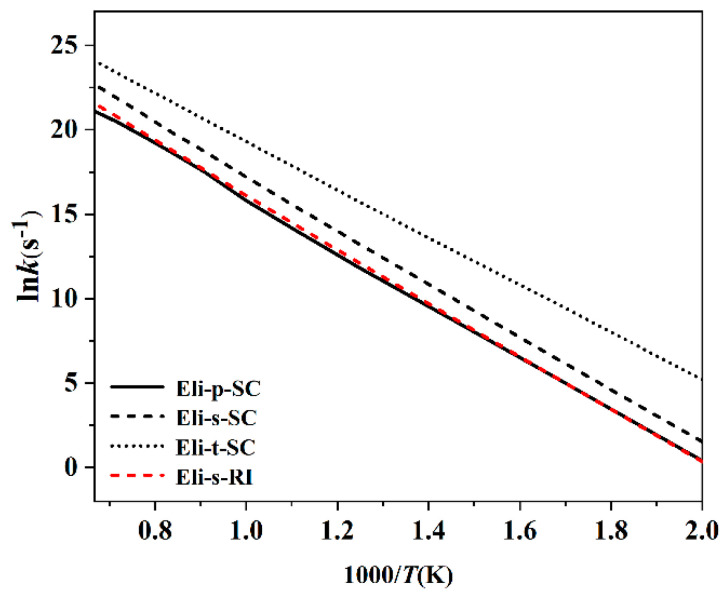
Comparisons of the rate rule derived for subclasses of the concerted elimination in this study.

The high-pressure-limit rate rules of the concerted elimination class of •OOQOOH in the range of 500–1500 K are shown in Figure 2. When reaction centers are located on the alkyl side chain, it can be seen from Figure 1 that the high-pressure-limit rate constants of subclass Eli-t-SC are larger than those of subclass Eli-s-SC, and the high-pressure-limit rate constants of subclass Eli-s-SC are larger than those of subclass Eli-p-SC. When reaction centers are located on the ring, it can be seen from Figure 1 that the high-pressure-limit rate constants of subclass Eli-s-RI are smaller than those of subclasses Eli-p-SC and Eli-s-SC. However, for the subclass Eli-s-RI, high-pressure-limit rate constants are very close to the values of the subclass Eli-p-SC.

#### 2.4.2. Comparison of the High-Pressure-Limit Rate Constants with the Literature Values

**Figure 3:**
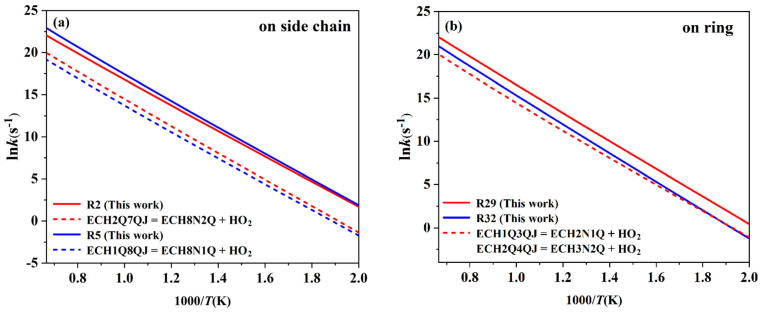
Comparison of the high-pressure-limit rate constants for concerted elimination reactions of •OOQOOH: (**a**) R2 and R5 correspond to ECH2Q7QJ = ECH8N2Q + HO_2_ and ECH1Q8QJ = ECH8N1Q + HO_2_ in the mechanism [29], respectively. (**b**) R29 and R32 correspond to ECH1Q3QJ = ECH2N1Q + HO_2_ and ECH2Q4QJ = ECH3N2Q + HO_2_ in the mechanism [29], respectively.

Zou et al. [29] constructed a detailed combustion mechanism for ethyl cyclohexane at low temperature. In this oxidation mechanism, the high-pressure-limit rate constants of the concerted elimination reactions of •OOQOOH were mainly quoted from the kinetic data of similar reactions in the literature. When the reaction centers were located on the side chain, the high-pressure-limit rate constants of the reactions were taken from Villano et al.’s high-pressure-limit rate rule for alkanes [30]. When the reaction centers were located on the ring, the high-pressure-limit rate constants of the reactions were taken from Fernandes et al.’s cyclohexane study and Xing et al.’s methyl cyclohexane study [31,32,33]. To see the difference between the high-pressure-limit rate constants calculated by us and the values of the similar reactions from the model of ethyl cyclohexane developed by Zou et al., comparisons for rate constants at the temperature of 500 K to 1500 K are given in Figure 3. When the reaction centers are located on the side chain, it can be seen from Figure 3a that the rate constants of R2 and R5 calculated in this study are all larger than the values of the similar reactions in the mechanism, with their ratios ranging from 7.9 to 20.9 and 37.0 to 43.0, respectively. This indicates a large deviation between the rate constants calculated in our study and those reported in the mechanism from Zou et al. When the reaction centers are located on the ring, the high-pressure-limit rate constants of reactions ECH1Q3QJ = ECH2N1Q + HO_2_ and ECH2Q4QJ = ECH3N2Q + HO_2_ from the mechanism are the same as the high-pressure-limit rate constants calculated in this study. It can be seen from Figure 3b that the rate constants of R29 and R32 calculated in this study are close to the values reported in the mechanism from Zou et al.

Mao et al. [34] developed a mechanism based on the combined model of Natelson et al. [35], which was used to simulate the ignition of n-butyl cyclohexane. Recently, a detailed kinetic model has been proposed by Mao et al. [36]. The model is composed of 1802 species and 7246 reactions, and it can simulate the ignition and oxidation of n-butylcyclohexane under various operating conditions. However, the concerted elimination reactions of •OOQOOH in n-butyl cyclohexane are not included in the above-detailed kinetic models. 

**Figure 4:**
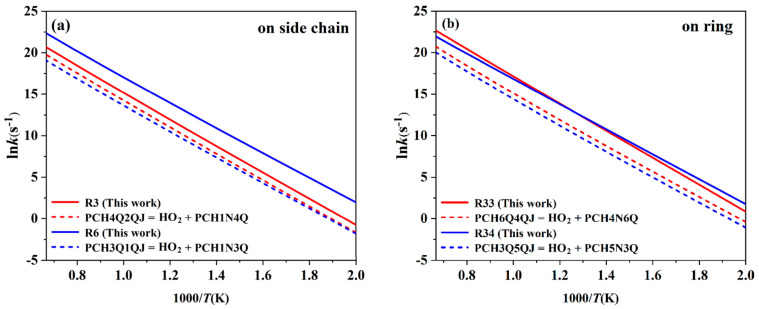
Comparison of the high-pressure-limit rate constants for concerted elimination reactions of •OOQOOH: (**a**) R3 and R6 correspond to PCH4Q2QJ = HO_2_ + PCH1N4Q and PCH3Q1QJ = HO_2_ + PCH1N3Q in the mechanism [37], respectively. (**b**) R33 and R34 correspond to PCH6Q4QJ = HO_2_ + PCH4N6Q and PCH3Q5QJ = HO_2_ + PCH5N3Q in the mechanism [37], respectively.

Very recently, a detailed chemical kinetic model covering low- to high-temperature combustion has been developed by Liu et al. [37] to describe n-propyl cyclohexane combustion, based on the combination of the comprehensive core mechanism taken from AramcoMech 3.0 and 34 low- to high-temperature reaction classes. When the reaction centers are located on the alkyl side chain of n-propyl cyclohexane, the rate constants of the concerted elimination reactions of •OOQOOH in the mechanism adopt the kinetic data of concerted elimination reactions of •OOQOOH with similar structures in alkanes [30]. The differences in these reaction rate constants are compared in Figure 4a. It can be seen from Figure 4a that the rate constants of R3 and R6 calculated in this study are both larger than those of similar reactions in mechanism, and their ratios are 2.4~2.5 and 26.7~43.3, respectively. When the reaction centers are located on the ring of n-propyl cyclohexane, the rate constants of the concerted elimination reactions of •OOQOOH in the mechanism use the kinetic data of the concerted elimination reactions of ROO• with a similar structure in cyclohexane [31]. The differences in these reaction rate constants are compared in Figure 4b. It can be seen from Figure 4b that the rate constants of R33 and R34 calculated in this study are larger than those of similar reactions in the mechanism, and their ratios are 3.5~7.7 and 6.7~17.4, respectively. Figure 4 shows that in the temperature range of 500–1500 K, there are certain deviations between the rate constants we calculated and the values reported in the literature.

### 2.5. Pressure-Dependent Rate Constants and Rate Rules 

The concerted elimination reactions of •OOQOOH belong to the pressure-dependent channel, and according to the RRKM/ME theory, the pressure-dependent rate constants for all reactions are calculated at pressures of 0.01–100 atm and temperatures of 500–1500 K. All calculated pressure-dependent rate constants are given in the form of (*A*, *n*, *E*), and they are listed in Tables S4 and S5 of Supplemental Material-I. The simple rate rule method is employed, which involves taking the average of the pressure-dependent rate constants for all reactions within each subclass to determine the corresponding pressure-dependent rate rules. The pressure-dependent rate rules are fitted to form (*A*, *n*, *E*), as shown in Table 5. To facilitate comparisons between different reactions or rate rules, the pressure-dependent rate constants at 800 K are also included in Table 5. Additionally, the ratios of the rate constants for each reaction to the average rate constant within a subclass are presented in the tables to visually illustrate the deviation of the rate constants within each subclass. Moreover, to assess the uncertainty of each subclass rate rule, the ratio of the maximum and minimum pressure-dependent rate constants within each subclass is defined as the uncertainty factor for that subclass, and the results are also listed in the tables. From Table 5, it can be observed that the ratios between the rate constants for reactions and the average rate constants within each subclass, as well as the uncertainty factors for each subclass, are all within 1~2 orders of magnitude. This indicates that the pressure-dependent rate rules constructed for the concerted elimination of •OOQOOH are reasonable and acceptable.

#### Comparison of the Pressure-Dependent Rate Rules at Different Pressures

**Figure 5:**
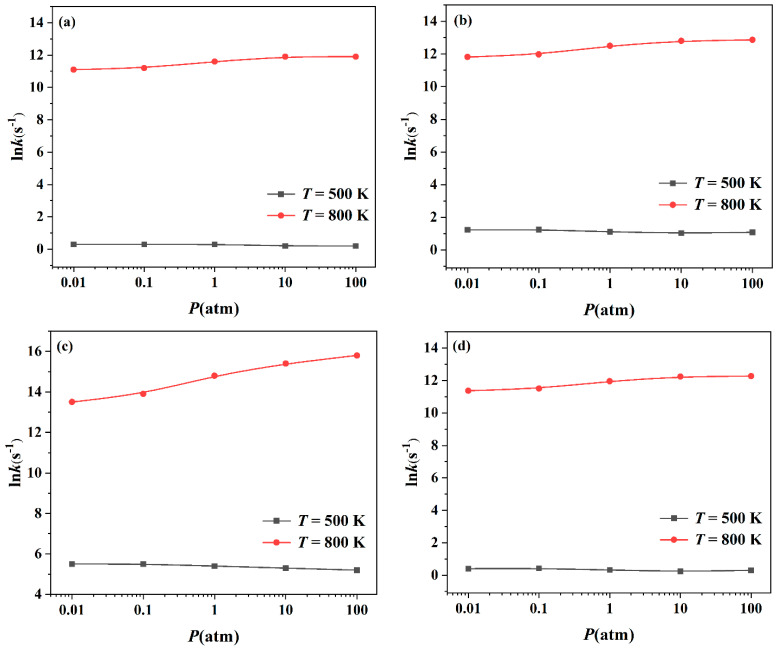
Comparisons of the pressure-dependent rate constants for concerted elimination reactions of •OOQOOH: (**a**) the subclass of Eli-p-SC; (**b**) the subclass of Eli-s-SC; (**c**) the subclass of Eli-t-SC; (**d**) the subclass of Eli-s-RI.

The rate constants of the reaction subclasses at certain temperatures are selected to observe the pressure dependence of our studied concerted elimination reactions of •OOQOOH, and Figure 5 is provided to show the pressure dependence of four subclasses at both 500 K and 800 K. As can be discerned from Figure 5, in the case of the Eli-p-SC subclass, the rate constants reach the high-pressure limit at 0.01 atm at the lower temperatures of 500 K, while the rate constants reach the high-pressure limit at 10 atm at the higher temperatures of 800 K. In the case of the Eli-s-SC subclass, the rate constants reach the high-pressure limit at 0.01 atm at the lower temperatures of 500 K, while the rate constants reach the high-pressure limit at 10 atm at the higher temperatures of 800 K. In the case of the Eli-t-SC subclass, the rate constants reach the high-pressure limit at 0.01 atm at the lower temperatures of 500 K. However, when the temperature rises to 800 K, the rate constants increase at the pressures from 0.01 to 100 atm, and even 100 atm is not enough to make the rate constants to reach the high-pressure limit. In the case of the Eli-s-RI subclass, the rate constants reach the high-pressure limit at 0.01 atm at the lower temperatures of 500 K, while the rate constants reach the high-pressure limit at 10 atm at the higher temperatures of 800 K. From the above comparison, it can be seen that the rate constants of the Eli-t-SC subclass are relatively strong on the pressure, while those of three subclasses are relatively weak on the pressure, indicating that the pressure effect of the Eli-t-SC subclass cannot be ignored, especially at high temperature.

## 3. Methods

### 3.1. Electronic Structure Calculation

The electronic structure calculations for all species in this study were performed using the Gaussian 16 quantum chemistry software package (Revision C. 01) [38]. Initially, all species were preliminarily optimized at the B3LYP/CBSB7 level, and then the intrinsic reaction coordinate (IRC) analysis [39] was performed at this theory level to confirm the reaction pathways and ensure that the transition states connect the correct reactants and products. Subsequently, the single-point energies of all species in this study were calculated using the CBS-QB3 method [40], and the Cartesian coordinates of all optimized structures in this study are provided in Supplemental Material-I.

### 3.2. Calculation of Rate Constant

This study utilized the ChemRate program [41] to obtain the high-pressure-limit rate constants and the pressure-dependent rate constants for all reactions. The calculations of these rate constants were based on the traditional transition state theory (TST) [42,43] and the expression as follows:

(1) $$k\left(T\right)=\kappa \left(T\right)rpd\frac{\kappa_{B}T}{h}\frac{Q_{TS}(T)}{Q_{R}(T)}exp\left[-\frac{V^{\neq }}{RT}\right]$$

In the expression, $\kappa \left(T\right)$, rpd, $\kappa_{B}$, and h are the Eckart tunneling correction factor, reaction path degeneracy, Boltzmann constant, and Planck constant, respectively. T is the temperature. $V^{\neq }$ is the height of the energy barrier. $Q_{TS}(T)$ and $Q_{R}(T)$ are the partition functions of the transition states and reactants, respectively.

The pressure-dependent rate constants were calculated using the RRKM/ME theory [44], and the collision frequency between the reactant and bath gas (Ar) was estimated by using the Lennard-Jones (L-J) parameters σ(Å) and ε(K). The L-J parameters were estimated with the method proposed by Wang and Frenklach [45], and σ(Å) and ε(K) used in this study are shown in Table S1 of Supplemental Material-I.

In this study, tunneling correction was performed using the one-dimensional asymmetric Eckart tunneling correction method [46,47]. Due to the large errors caused by treating low-frequency vibrations of single bond torsions with harmonic approximation [48], a one-dimensional (1-D) hindered rotor model [49] was used to handle the low-frequency vibrations of single bond torsions in the reactions, with a frequency correction factor of 0.99 [40]. The torsional potential energies involved in the reactants were obtained by scanning the dihedral angles every 10° at the B3LYP/CBSB7 theoretical level. When scanning the transition states, all atoms involved in the reaction centers were fixed to ensure that the reaction centers remained unchanged during the scanning process, and only the remaining single bonds were scanned for torsion. When the torsional potential energies exceeded 10 kcal mol^−1^, the torsional motions were approximated as harmonic vibration [50]. In addition, thermodynamic parameters such as enthalpy, entropy, and heat capacity were fitted into 14 parameters in the NASA format to facilitate combustion modeling using practical modeling software such as Chemkin-PRO (15092) [51], and the results are given in Supplemental Material-II.

Finally, the rate constants from 500 K to 1500 K were fitted using the three-parameter form of the Arrhenius equation:

(2) $$k\left(T\right)=AT^{n}\exp(-\frac{E}{RT})$$

## 4. Conclusions

In this study, the energy barriers, the high-pressure-limit rate constant and the pressure-dependent rate constants of the concerted elimination reactions of normal-alkyl cyclohexanes were calculated, and the following conclusions can be drawn: (1) The energy barriers and the rate constants among the class of concerted elimination reactions of normal-alkyl cyclohexanes have a large difference, so this reaction class should be further divided into different reaction subclasses according to the type of carbon atom in which the H atom is eliminated and whether the reaction center is located on the ring. Accordingly, the high-pressure-limit rate rules and the pressure-dependent rate rules of the corresponding subclasses were constructed. (2) The maximum energy barrier deviations and the rate rule uncertainty factors in the same subclass were within the range of chemical accuracy, which indicates that our classification schemes are acceptable. (3) The rate constants of the concerted elimination reactions of normal-alkyl cyclohexanes were very different from the kinetic data of similar reactions in some published mechanisms, which indicates that it is important to establish the rate rules of the concerted elimination reactions of normal-alkyl cyclohexanes. (4) The pressures had significant effects on the rate constants of concerted elimination reactions of normal-alkyl cyclohexanes. The high-pressure-limit rate rules and pressure-dependent rate rules provided in this paper are applicable to the actual combustion process of normal-alkyl cyclohexanes in the temperature range of 500–1500 K and pressure range of 0.01–100 atm. Therefore, our accurately calculated rate rules are of great significance, and they will contribute to the development of the automatic generation of a combustion mechanism for normal-alkyl cyclohexanes.

## Figures and Tables

**Table 1:** Energy barriers for the reaction center of concerted elimination class occur on the alkyl side chain (kcal mol^−1^).

Subclass		Reaction	Energy Barrier
**Eli-p-SC**	R1	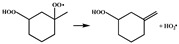	32.3
	R2	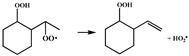	29.3
	R3	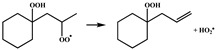	31.0
	R4	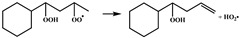	32.5
			**(31.3 ^a^, 3.2 ^b^)**
**Eli-s-SC**	R5	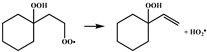	29.8
	R6	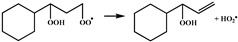	28.9
	R7	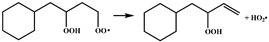	30.8
	R8	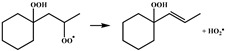	30.0
	R9	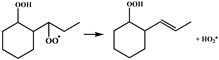	29.0
	R10	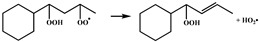	30.6
	R11	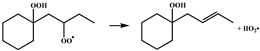	30.7
	R12	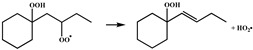	30.5
	R13	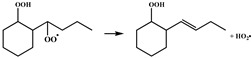	28.8
	R14	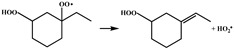	31.8
	R15	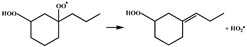	31.6
	R16	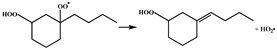	31.5
			**(30.3 ^a^, 3.0 ^b^)**
**Eli-t-SC**	R17	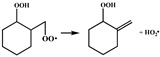	26.7
	R18	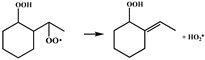	26.8
	R19	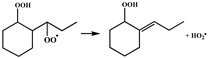	26.8
	R20	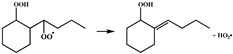	26.6
			**(26.7 ^a^, 0.2 ^b^)**

^a^ The average energy barrier of reactions in each subclass. ^b^ The maximum deviation of energy barriers in each subclass.

**Table 2:** Energy barriers for the reaction center of concerted elimination class occur on ring (kcal mol^−1^).

Subclass		Reaction	Energy Barrier
**Eli-s-RI**	R21	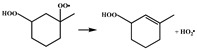	29.4
	R22	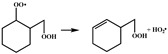	32.5
	R23	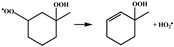	31.6
	R24	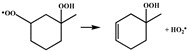	31.0
	R25	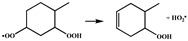	30.8
	R26	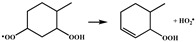	30.8
	R27	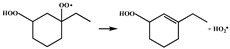	31.6
	R28	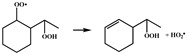	31.9
	R29	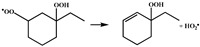	31.3
	R30	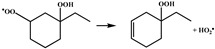	31.0
	R31	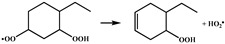	30.9
	R32	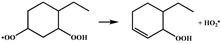	32.2
	R33	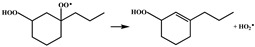	31.5
	R34	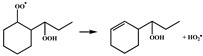	29.4
	R35	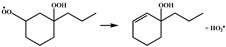	31.4
	R36	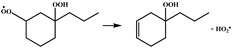	31.0
	R37	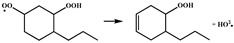	30.9
	R38	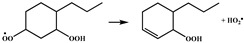	31.6
	R39	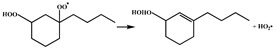	31.5
	R40	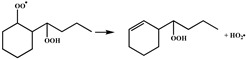	31.8
	R41	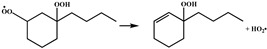	31.4
	R42	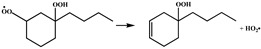	32.8
	R43	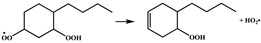	30.9
	R44	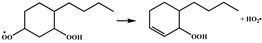	32.2
			**(31.3 ^a^, 3.4 ^b^)**

^a^ The average energy barrier of reactions in each subclass. ^b^ The maximum deviation of energy barriers in each subclass.

**Table 3:** Calculated rate constants, rate rules, ratios of rate constants of reactions to the average value of the reactions and uncertainty factor values of the rate rule in each subclass for the reaction center of concerted elimination occurring on the alkyl side chain at high pressure.

	Modified Arrhenius Parameters			*T* = 800 K	
Reaction	*A* (s^−1^)	*n*	*E* (cal mol^−1^)	*k* (s^−1^)	*k/k_ave_* ^e^
R1	4.93 × 10^7^	1.81	30,533.6	4.0 × 10^4^	0.3
R2	2.05 × 10^8^	1.43	28,588.6	4.2 × 10^5^	3.1
R3	7.84 × 10^9^	1.09	30,111.4	6.9 × 10^4^	0.5
R4	3.79 × 10^56^	−13.32	49,803.3	2.2 × 10^4^	0.2
**Eli-p-SC rate rule**	**1.57 × 10^8^**	**1.64**	**28,481.2**	**1.4 × 10^5^**	**19.1 ^#^**
R5	3.79 × 10^8^	1.74	28,497.1	6.9 × 10^5^	1.4
R6	2.57 × 10^8^	1.67	27,626.4	5.3 × 10^5^	1.0
R7	4.00 × 10^9^	1.68	29,468.1	2.8 × 10^6^	5.2
R8	1.86 × 10^10^	1.19	29,349.6	5.3 × 10^5^	1.0
R9	2.47 × 10^10^	1.20	29,350.9	7.0 × 10^5^	1.3
R10	5.18 × 10^11^	0.52	30,599	7.1 × 10^4^	0.1
R11	7.74 × 10^11^	0.52	30,600	1.1 × 10^5^	0.2
R12	2.16 × 10^10^	1.19	29,866.6	4.2 × 10^5^	0.8
R13	4.12 × 10^10^	0.98	28,308.2	5.4 × 10^5^	1.0
R14	1.11 × 10^11^	0.61	31,317.3	1.8 × 10^4^	0.03
R15	1.25 × 10^10^	0.78	30,983.5	8.1 × 10^3^	0.02
R16	3.49 × 10^8^	1.43	30,593.3	2.2 × 10^4^	0.04
**Eli-s-SC rate rule**	**2.60 × 10^10^**	**1.08**	**30,836.8**	**5.3 × 10^5^**	**24.4 ^#^**
R17	2.80 × 10^8^	1.57	25,706.1	9.8 × 10^5^	0.1
R18	5.47 × 10^9^	1.42	26,183	5.1 × 10^6^	0.8
R19	8.18 × 10^9^	1.42	26,184.1	7.6 × 10^6^	1.1
R20	1.20 × 10^13^	0.47	26,879.2	1.3 × 10^7^	1.9
**Eli-t-SC rate rule**	**8.58 × 10^10^**	**1.06**	**26,361.1**	**6.6 × 10^6^**	**13.1 ^#^**

^#^ The uncertainty factor values of the high-pressure-limit rule at 800 K for each subclass in our work (the ratios of the largest rate constant to the smallest rate constant at 800 K for each subclass). ^e^ *k_ave_* is the average rate constant at 800 K for each reaction subclass.

**Table 4:** Calculated rate constants, rate rules, ratios of rate constants of reactions to the average value of the reactions and uncertainty factor values of the rate rule in each subclass for the reaction center of concerted elimination occurring on the ring.

	Modified Arrhenius Parameters			*T* = 800 K	
Reaction	*A* (s^−1^)	*n*	*E* (cal mol^−1^)	*k* (s^−1^)	*k/k_ave_* ^e^
R21	1.51 × 10^12^	0.51	29,246.6	4.6 × 10^5^	2.6
R22	6.39 × 10^8^	1.42	27,224.4	3.1 × 10^5^	1.7
R23	4.89 × 10^10^	1.01	30,984.9	1.4 × 10^5^	0.8
R24	1.27 × 10^11^	0.79	30,377.0	1.3 × 10^5^	0.7
R25	5.33 × 10^10^	0.80	30,115.3	6.4 × 10^4^	0.4
R26	5.22 × 10^10^	0.80	30,107.6	6.4 × 10^4^	0.4
R27	2.44 × 10^9^	1.46	30,788.5	1.6 × 10^5^	0.9
R28	4.58 × 10^5^	2.18	30,452.6	4.6 × 10^3^	0.03
R29	1.55 × 10^11^	0.90	30,708.6	2.5 × 10^5^	1.4
R30	4.57 × 10^10^	0.81	30,372.2	5.1 × 10^4^	0.3
R31	2.78 × 10^8^	1.60	29,764.3	8.9 × 10^4^	0.5
R32	6.66 × 10^10^	0.91	31,607	6.7 × 10^4^	0.4
R33	5.56 × 10^12^	0.54	31,618.5	4.6 × 10^5^	2.6
R34	1.51 × 10^12^	0.51	29,246.6	4.6 × 10^5^	2.6
R35	4.60 × 10^6^	1.94	30,292	1.0 × 10^4^	0.1
R36	3.56 × 10^11^	0.50	30,800.5	3.9 × 10^4^	0.2
R37	1.16 × 10^12^	0.52	30,713.4	1.6 × 10^5^	0.9
R38	2.60 × 10^10^	1.08	30,836.8	1.3 × 10^5^	0.8
R39	2.34 × 10^12^	0.73	31,431.1	7.9 × 10^5^	4.5
R40	6.75 × 10^10^	0.95	31,069.9	1.3 × 10^5^	0.7
R41	6.38 × 10^10^	0.97	30,914.1	1.5 × 10^5^	0.8
R42	2.72 × 10^10^	0.99	31,994.3	3.7 × 10^4^	0.2
R43	8.30 × 10^8^	1.27	30,107	2.3 × 10^4^	0.1
R44	3.11 × 10^9^	1.38	31,484.1	7.8 × 10^4^	0.4
**Eli-s-RI rate rule**	**2.40 × 10^9^**	**1.35**	**29,501.2**	**1.8 × 10^5^**	**30.0 ^#^**

^#^ The uncertainty factor values of the high-pressure-limit rule at 800 K for each subclass in our work (the ratios of the largest rate constant to the smallest rate constant at 800 K for each subclass). ^e^ *k_ave_* is the average rate constant at 800 K for each reaction subclass.

**Table 5:** Pressure-dependent rate rules for the concerted elimination of •OOQOOH (*A*, *n*, *E*).

		Modified Arrhenius Parameters			*T* = 800 K	
Reaction	Pressure (atm)	*A* (s^−1^)	*n*	*E* (cal mol^−1^)	*k* (s^−1^)	*k/k_ave_* ^e^
Eli-p-SC rate rule	0.01	4.60 × 10^54^	−12.97	44,652.8	6.5 × 10^4^	8.1 ^#^
	0.1	4.34 × 10^53^	−12.57	44,825.9	7.4 × 10^4^	9.3 ^#^
	1	4.10 × 10^39^	−8.13	40,143.2	1.1 × 10^5^	13.2 ^#^
	10	1.91 × 10^33^	−6.07	38,490.7	1.4 × 10^5^	17.2 ^#^
	100	3.90 × 10^19^	−1.86	33,084.3	1.4 × 10^5^	18.7 ^#^
Eli-s-SC rate rule	0.01	7.66 × 10^59^	−14.54	46,136.0	1.4 × 10^5^	31.1 ^#^
	0.1	2.50 × 10^53^	−12.49	43,974.3	1.6 × 10^5^	14.6 ^#^
	1	3.07 × 10^49^	−11.07	43,941.0	2.7 × 10^5^	21.4 ^#^
	10	2.31 × 10^42^	−8.75	42,013.0	3.6 × 10^5^	27.0 ^#^
	100	1.48 × 10^27^	−4.05	36,249.4	3.9 × 10^5^	30.1 ^#^
Eli-t-SC rate rule	0.01	6.43 × 10^54^	−13.30	37,835.8	7.4 × 10^5^	4.2 ^#^
	0.1	7.17 × 10^46^	−10.71	35,555.7	1.1 × 10^6^	4.8 ^#^
	1	6.74 × 10^54^	−12.82	40,914.0	2.7 × 10^6^	6.2 ^#^
	10	8.99 × 10^50^	−11.38	41,058.3	5.1 × 10^6^	7.9 ^#^
	100	4.00 × 10^40^	−8.03	38,136.5	7.4 × 10^6^	11.3 ^#^
Eli-s-RI rate rule	0.01	1.57 × 10^60^	−14.59	47,219.6	8.6 × 10^4^	13.9 ^#^
	0.1	4.79 × 10^53^	−12.53	45,013.8	9.9 × 10^4^	16.4 ^#^
	1	9.77 × 10^45^	−9.99	43,234.3	1.6 × 10^5^	25.0 ^#^
	10	8.58 × 10^39^	−8.00	41,735.5	2.1 × 10^5^	34.2 ^#^
	100	7.40 × 10^24^	−3.36	35,886.8	2.1 × 10^5^	36.6 ^#^

^#^ The uncertainty factor values of the high-pressure-limit rule at 800 K for each subclass in our work (the ratios of the largest rate constant to the smallest rate constant at 800 K for each subclass). ^e^ *k_ave_* is the average rate constant at 800 K for each reaction subclass.

## Data Availability

Not applicable.

## Supplementary Materials

The following supporting information can be downloaded at: https://www.mdpi.com/article/10.3390/molecules28186612/s1, The Lennard-Jones parameters used in this work, high-pressure-limit rate constants, pressure-dependent rate constants, and the Cartesian coordinates for all reactants, products and transition states in this work are given in Supplemental Material-I. The thermodynamic parameters including the enthalpies, entropies, and heat capacities in the NASA format with 14 parameters are given in Supplemental Material-II.
