# Supplementary material for: A Theoretical Kinetic Study on Concerted Elimination Reaction Class of Peroxyl-hydroperoxyl-alkyl Radicals (•OOQOOH) in Normal-alkyl Cyclohexanes
Source: Molecules. 2023 Sep 14;28(18):6612. doi: 10.3390/molecules28186612 (PMC10536632; doi:10.3390/molecules28186612)
Supplement: Supplementary file 1 [file molecules-28-06612-s001.zip › Supplemental Material-I.pdf]

Supplemental Material-I

A Theoretical Kinetic Study on Concerted Elimination Reaction Class of Peroxyl-hydroperoxyl-alkyl Radicals (•OOQOOH) in Normal-alkyl Cyclohexanes

Xiaoxia Yao [1], Jilong Zhang [2,*] and Yifei Zhu [3,*]

1 National Key Lab of Aerospace Power System and Plasma Technology, Air Force Engineering University, Xi'an 710038, China; yaoxxkgd@163.com
2 Institute of Theoretical Chemistry, College of Chemistry, Jilin University, Changchun 130023, China
3 School of Electrical Engineering, Xi'an Jiaotong University, Xian 710049, China
* Correspondence: jilongzhang@jlu.edu.cn (J.Z.); yifei.zhu.plasma@gmail.com (Y.Z.)

Contents:

1. Lennard-Jones parameters used in this work

Table S1. Lennard-Jones parameters σ (Å) and ε (K) used in this work.

2. High-pressure limit rate constants of the class of concerted elimination

Table S2. Calculated rate constants, rate rules, ratios of rate constants of reactions to the average value of the reactions and uncertainty factor values of the rate rule in each subclass for the reaction center of concerted elimination occurring on the alkyl side chain at high pressure.

Table S3. Calculated rate constants, rate rules, ratios of rate constants of reactions to the average value of the reactions and uncertainty factor values of the rate rule in each subclass for the reaction center of concerted elimination occurring on the ring.

3. Pressure-dependent rate constants of the concerted elimination class

Table S4. Calculated rate constants, rate rules, ratios of rate constants of reactions to the average value of the reactions and uncertainty factor values of the rate rule in each subclass for the reaction center of concerted elimination occurring on the alkyl side chain at different pressures.

Table S5. Calculated rate constants, rate rules, ratios of rate constants of reactions to the average value of the reactions and uncertainty factor values of the rate rule in each class for the reaction center of concerted elimination occurring on the ring at different pressures.

4. Cartesian coordinates for all reactants, transition states and products in the class of concerted elimination

1. Lennard-Jones parameters used in this work

The collision frequency between the reactant and bath gas (Ar) is estimated by using the Lennard-Jones (L-J) parameters σ (Å) and ε (K). The L-J parameters for species are estimated using the method proposed by Wang and co-workers [1].

Table S1. Lennard-Jones parameters σ (Å) and ε (K) used in this work.

Reactant[a]	σ	ε
$C_7H_{12}OOHOO$	6.72	707.81
$C_8H_{14}OOHOO$	6.91	742.88
$C_9H_{16}OOHOO$	7.09	776.80
$C_{10}H_{18}OOHOO$	7.26	809.67
Ar	3.47	114

[a]Represents the reactants in the class of cyclization.

[1] Wang, H.; Frenklach, M. Transport properties of polycyclic aromatic hydrocarbons for flame modeling. Combust. Flame **1994**, 96, 163-170.

2. High-pressure limit rate constants of the class of concerted elimination

Table S2. Calculated rate constants, rate rules, ratios of rate constants of reactions to the average value of the reactions and uncertainty factor values of the rate rule in each subclass for the reaction center of concerted elimination occurring on the alkyl side chain at high pressure.

Reaction	Modified Arrhenius parameters			T=800 K	
	A (s^{-1})	n	E (cal mol^{-1})	k (s^{-1})	$k/k_{ave}{}^{e}$
R1	4.93E+07	1.81	30533.6	4.0E+04	0.3
R2	2.05E+08	1.43	28588.6	4.2E+05	3.1
R3	7.84E+09	1.09	30111.4	6.9E+04	0.5
R4	3.79E+56	-13.32	49803.3	2.2E+04	0.2
**Eli-p-SC rate rule**	**1.57E+08**	**1.64**	**28481.2**	**1.4E+05**	**19.1#**
R5	3.79E+08	1.74	28497.1	6.9E+05	1.4
R6	2.57E+08	1.67	27626.4	5.3E+05	1.0
R7	4.00E+09	1.68	29468.1	2.8E+06	5.2
R8	1.86E+10	1.19	29349.6	5.3E+05	1.0
R9	2.47E+10	1.20	29350.9	7.0E+05	1.3
R10	5.18E+11	0.52	30599	7.1E+04	0.1
R11	7.74E+11	0.52	30600	1.1E+05	0.2
R12	2.16E+10	1.19	29866.6	4.2E+05	0.8
R13	4.12E+10	0.98	28308.2	5.4E+05	1.0
R14	1.11E+11	0.61	31317.3	1.8E+04	0.03
R15	1.25E+10	0.78	30983.5	8.1E+03	0.02
R16	3.49E+08	1.43	30593.3	2.2E+04	0.04
**Eli-s-SC rate rule**	**2.60E+10**	**1.08**	**30836.8**	**5.3E+05**	**24.4#**
R17	2.80E+08	1.57	25706.1	9.8E+05	0.1

R18	5.47E+09	1.42	26183	5.1E+06	0.8
R19	8.18E+09	1.42	26184.1	7.6E+06	1.1
R20	1.20E+13	0.47	26879.2	1.3E+07	1.9
**Eli-t-SC rate rule**	**8.58E+10**	**1.06**	**26361.1**	**6.6E+06**	**13.1#**

#The uncertainty factor values of the high-pressure limit rule at 800 K for each subclass in our work (The ratios of the largest rate constant of a reaction to the smallest rate constant at 800 K for each subclass).
ek_{ave} is the average rate constants at 800 K for each reaction subclass.

Table S3. Calculated rate constants, rate rules, ratios of rate constants of reactions to the average value of the reactions and uncertainty factor values of the rate rule in each subclass for the reaction center of concerted elimination occurring on the ring.

	**Modified Arrhenius parameters**			***T*=800 K**	
**Reaction**	***A* (s^{-1})**	***n***	***E* (cal mol^{-1})**	***k* (s^{-1})**	**k/k_{ave}^{e}**
R21	1.51E+12	0.51	29246.6	4.6E+05	2.6
R22	6.39E+08	1.42	27224.4	3.1E+05	1.7
R23	4.89E+10	1.01	30984.9	1.4E+05	0.8
R24	1.27E+11	0.79	30377.0	1.3E+05	0.7
R25	5.33E+10	0.80	30115.3	6.4E+04	0.4
R26	5.22E+10	0.80	30107.6	6.4E+04	0.4
R27	2.44E+09	1.46	30788.5	1.6E+05	0.9
R28	4.58E+05	2.18	30452.6	4.6E+03	0.03
R29	1.55E+11	0.90	30708.6	2.5E+05	1.4
R30	4.57E+10	0.81	30372.2	5.1E+04	0.3
R31	2.78E+08	1.60	29764.3	8.9E+04	0.5
R32	6.66E+10	0.91	31607	6.7E+04	0.4
R33	5.56E+12	0.54	31618.5	4.6E+05	2.6
R34	1.51E+12	0.51	29246.6	4.6E+05	2.6
R35	4.60E+06	1.94	30292	1.0E+04	0.1
R36	3.56E+11	0.50	30800.5	3.9E+04	0.2
R37	1.16E+12	0.52	30713.4	1.6E+05	0.9
R38	2.60E+10	1.08	30836.8	1.3E+05	0.8
R39	2.34E+12	0.73	31431.1	7.9E+05	4.5
R40	6.75E+10	0.95	31069.9	1.3E+05	0.7
R41	6.38E+10	0.97	30914.1	1.5E+05	0.8
R42	2.72E+10	0.99	31994.3	3.7E+04	0.2
R43	8.30E+08	1.27	30107	2.3E+04	0.1
R44	3.11E+09	1.38	31484.1	7.8E+04	0.4
**Eli-s-RI rate rule**	**2.40E+09**	**1.35**	**29501.2**	**1.8E+05**	**30.0#**

#The uncertainty factor values of the high-pressure limit rule at 800 K for each class in our work (The ratios of the largest rate constant of a reaction to the smallest rate constant at 800 K for each subclass).
ek_{ave} is the average rate constants at 800 K for each reaction subclass.

3. Pressure-dependent rate constants of the concerted elimination class

Table S4. Calculated rate constants, rate rules, ratios of rate constants of reactions to the average value of the reactions and uncertainty factor values of the rate rule in each subclass for the reaction center of concerted elimination occurring on the alkyl side chain at different pressures.

Reaction	Pressure (atm)	Modified Arrhenius parameters			*T*=800K	
		A (s^{-1})	*n*	*E* (cal mol^{-1})	*k* (s^{-1})	k/k_{ave}^{e}
R1	0.01	1.72E+61	-14.81	50468.6	2.8E+04	0.4
	0.1	1.86E+54	-12.63	47992.3	3.1E+04	0.4
	1	8.91E+41	-8.72	43980.6	4.1E+04	0.4
	10	4.23E+27	-4.26	38719.0	4.7E+04	0.3
	100	2.57E+15	-0.52	33804.6	4.6E+04	0.3
R2	0.01	2.66E+62	-15.32	46656.6	1.6E+05	2.5
	0.1	3.17E+54	-12.82	43937.4	1.9E+05	2.6
	1	5.91E+45	-9.94	41839.2	3.1E+05	2.8
	10	4.93E+41	-8.54	41262.3	4.3E+05	3.0
	100	1.12E+25	-3.42	34725.0	4.3E+05	3.0
R3	0.01	9.41E+59	-14.43	49023.7	5.0E+04	0.8
	0.1	2.32E+53	-12.37	46577.9	5.3E+04	0.7
	1	2.59E+40	-8.30	42137.7	6.6E+04	0.6
	10	1.52E+32	-5.70	39392.8	7.7E+04	0.5
	100	6.27E+17	-1.31	33465.2	7.2E+04	0.5
R4	0.01	3.79E+56	-13.32	49803.3	2.0E+04	0.3
	0.1	6.53E+50	-11.54	47603.1	2.1E+04	0.3
	1	5.23E+36	-7.15	42406.1	2.3E+04	0.2
	10	1.82E+25	-3.60	38099.1	2.5E+04	0.2
	100	1.39E+14	-0.22	33459.4	2.3E+04	0.2
**Eli-p-SC rate rule**	**0.01**	**4.60E+54**	**-12.97**	**44652.8**	**6.5E+04**	**8.1#**
	**0.1**	**4.34E+53**	**-12.57**	**44825.9**	**7.4E+04**	**9.3#**
	**1**	**4.10E+39**	**-8.13**	**40143.2**	**1.1E+05**	**13.2#**
	**10**	**1.91E+33**	**-6.07**	**38490.7**	**1.4E+05**	**17.2#**
	**100**	**3.90E+19**	**-1.86**	**33084.3**	**1.4E+05**	**18.7#**
R5	0.01	7.24E+64	-16.06	47315.2	2.0E+05	1.7
	0.1	3.64E+56	-13.43	44591.5	2.5E+05	1.9
	1	3.26E+54	-12.52	45635.2	4.9E+05	2.1
	10	3.15E+47	-10.21	43937.7	7.0E+05	2.3
	100	9.42E+30	-5.09	37761.4	7.8E+05	2.4
R6	0.01	4.23E+63	-15.68	46640.3	2.3E+05	1.7
	0.1	1.91E+55	-13.05	43778.3	2.7E+05	1.7
	1	7.86E+47	-10.56	42357.1	4.6E+05	1.7
	10	6.26E+44	-9.45	42267.6	6.6E+05	1.8
	100	3.88E+27	-4.14	35623.6	7.0E+05	1.8
R7	0.01	3.22E+66	-16.41	50501.2	1.2E+05	0.9

	0.1	3.56E+58	-13.92	47644.5	1.3E+05	0.9
	1	3.12E+47	-10.36	44396.5	2.0E+05	0.7
	10	7.53E+42	-8.81	43520.7	2.6E+05	0.7
	100	1.21E+25	-3.35	36423.0	2.6E+05	0.7
R8	0.01	3.16E+66	-16.49	48741.6	2.0E+05	1.5
	0.1	1.35E+58	-13.85	45864.2	2.4E+05	1.5
	1	3.24E+56	-13.11	46940.4	4.3E+05	1.6
	10	3.35E+46	-9.92	43825.2	5.7E+05	1.6
	100	8.67E+29	-4.79	37529.3	6.1E+05	1.6
R9	0.01	7.80E+66	-16.63	48504.3	2.3E+05	1.7
	0.1	2.34E+58	-13.93	45613.7	2.8E+05	1.8
	1	1.26E+59	-13.89	47759.1	5.4E+05	2.0
	10	2.63E+48	-10.48	44426.8	7.3E+05	2.0
	100	1.32E+32	-5.42	38349.9	8.1E+05	2.1
R10	0.01	6.87E+59	-14.37	48862.8	5.8E+04	0.4
	0.1	3.04E+53	-12.40	46486.3	6.1E+04	0.4
	1	2.71E+40	-8.30	41944.8	7.4E+04	0.3
	10	3.71E+32	-5.82	39337.4	8.6E+04	0.2
	100	7.17E+18	-1.64	33654.6	8.0E+04	0.2
R11	0.01	2.02E+62	-15.12	49451.1	7.8E+04	0.6
	0.1	2.85E+55	-12.99	46918.3	8.3E+04	0.5
	1	7.74E+42	-9.02	42717.3	1.1E+05	0.4
	10	1.58E+36	-6.88	40704.2	1.3E+05	0.4
	100	8.83E+20	-2.22	34448.3	1.2E+05	0.3
R12	0.01	2.21E+68	-16.99	50305.7	1.9E+05	1.4
	0.1	8.88E+59	-14.35	47349.2	2.2E+05	1.4
	1	1.79E+57	-13.29	47891.8	3.7E+05	1.4
	10	1.37E+47	-10.08	44658.7	4.8E+05	1.3
	100	1.24E+30	-4.81	38131.0	5.1E+05	1.3
R13	0.01	1.54E+64	-15.82	46972.7	2.6E+05	1.9
	0.1	8.12E+55	-13.22	44060.5	3.0E+05	1.9
	1	2.94E+50	-11.33	43412.0	5.0E+05	1.9
	10	3.98E+44	-9.40	41994.7	6.8E+05	1.9
	100	5.78E+27	-4.21	35473.5	7.0E+05	1.8
R14	0.01	8.40E+52	-12.30	47633.1	1.6E+04	0.1
	0.1	3.34E+47	-10.63	45590.5	1.7E+04	0.1
	1	1.60E+34	-6.48	40675.3	1.9E+04	0.1
	10	5.69E+21	-2.65	35826.7	1.9E+04	0.1
	100	2.27E+14	-0.39	32761.6	1.8E+04	0.05
R15	0.01	1.16E+45	-9.94	44952.6	8.5E+03	0.1
	0.1	7.81E+40	-8.65	43324.3	8.6E+03	0.1
	1	1.68E+28	-4.75	38413.2	8.8E+03	0.03
	10	3.12E+16	-1.16	33674.7	8.6E+03	0.02
	100	3.87E+11	0.33	31620.7	8.3E+03	0.02

R16	0.01	1.79E+55	-12.96	48734.6	2.0E+04	0.1
	0.1	3.15E+49	-11.18	46553.3	2.1E+04	0.1
	1	5.00E+35	-6.89	41496.0	2.4E+04	0.1
	10	4.60E+24	-3.46	37356.0	2.5E+04	0.1
	100	5.05E+13	-0.13	32778.1	2.3E+04	0.1
**Eli-s-SC rate rule**	**0.01**	**7.66E+59**	**-14.54**	**46136.0**	**1.4E+05**	**31.1**[#]
	**0.1**	**2.50E+53**	**-12.49**	**43974.3**	**1.6E+05**	**14.6**[#]
	**1**	**3.07E+49**	**-11.07**	**43941.0**	**2.7E+05**	**21.4**[#]
	**10**	**2.31E+42**	**-8.75**	**42013.0**	**3.6E+05**	**27.0**[#]
	**100**	**1.48E+27**	**-4.05**	**36249.4**	**3.9E+05**	**30.1**[#]
R17	0.01	9.31E+56	-13.85	41273.8	3.0E+05	0.4
	0.1	9.78E+48	-11.30	38731.9	3.9E+05	0.4
	1	6.64E+43	-9.46	38408.4	7.5E+05	0.3
	10	1.10E+43	-9.02	39463.0	1.2E+06	0.2
	100	7.92E+26	-4.02	33342.9	1.3E+06	0.2
R18	0.01	8.86E+56	-13.91	39392.3	6.5E+05	0.9
	0.1	4.99E+48	-11.24	36969.7	9.5E+05	0.9
	1	5.38E+58	-13.95	43365.8	2.4E+06	0.9
	10	7.70E+50	-11.33	41647.5	4.1E+06	0.8
	100	3.30E+38	-7.39	37741.7	5.6E+06	0.8
R19	0.01	5.94E+55	-13.58	38297.9	7.5E+05	1.0
	0.1	4.96E+47	-10.95	36020.9	1.1E+06	1.0
	1	9.36E+58	-14.04	42932.4	3.1E+06	1.1
	10	2.15E+52	-11.76	41865.8	5.6E+06	1.1
	100	1.95E+41	-8.20	38669.6	8.2E+06	1.1
R20	0.01	5.34E+55	-13.57	37438.5	1.3E+06	1.7
	0.1	1.30E+47	-10.78	34938.9	1.9E+06	1.7
	1	4.18E+57	-13.67	41229.6	4.7E+06	1.7
	10	8.92E+53	-12.26	41677.1	9.4E+06	1.9
	100	5.47E+44	-9.24	39382.1	1.5E+07	2.0
**Eli-t-SC rate rule**	**0.01**	**6.43E+54**	**-13.30**	**37835.8**	**7.4E+05**	**4.2**[#]
	**0.1**	**7.17E+46**	**-10.71**	**35555.7**	**1.1E+06**	**4.8**[#]
	**1**	**6.74E+54**	**-12.82**	**40914.0**	**2.7E+06**	**6.2**[#]
	**10**	**8.99E+50**	**-11.38**	**41058.3**	**5.1E+06**	**7.9**[#]
	**100**	**4.00E+40**	**-8.03**	**38136.5**	**7.4E+06**	**11.3**[#]

[#]The uncertainty factor values of the high-pressure limit rule at 800 K for each subclass in our work (The ratios of the largest rate constant of a reaction to the smallest rate constant at 800 K for each subclass).

[e]k_{ave} is the average rate constants at 800 K for each reaction subclass.

Table S5. Calculated rate constants, rate rules, ratios of rate constants of reactions to the average value of the reactions and uncertainty factor values of the rate rule in each class for the reaction center of concerted elimination occurring on the ring at different pressures.

		Modified Arrhenius parameters			*T*=800K	
**Reaction**	**Pressure (atm)**	***A*** **(s^{-1})**	***n***	***E*** **(cal mol^{-1})**	***k*** **(s^{-1})**	k/k_{ave}^{e}
R21	0.01	4.96E+63	-15.67	47180.9	2.0E+05	2.3
	0.1	4.84E+55	-13.16	44383.5	2.3E+05	2.3
	1	5.11E+46	-10.21	42150.4	3.7E+05	2.4
	10	3.00E+43	-9.07	41908.5	5.1E+05	2.5
	100	5.90E+26	-3.93	35328.8	5.1E+05	2.4
R22	0.01	1.05E+59	-14.36	44433.2	1.5E+05	1.8
	0.1	2.14E+51	-11.93	41857.2	1.8E+05	1.8
	1	3.70E+43	-9.34	40190.4	3.0E+05	1.9
	10	2.12E+38	-7.59	39090.9	4.1E+05	2.0
	100	3.30E+22	-2.72	32912.9	4.2E+05	2.0
R23	0.01	3.35E+65	-16.17	50199.0	7.6E+04	0.9
	0.1	7.70E+57	-13.75	47624.7	9.0E+04	0.9
	1	5.90E+48	-10.76	45344.9	1.4E+05	0.9
	10	2.52E+42	-8.66	43773.6	1.9E+05	0.9
	100	7.95E+25	-3.59	37306.6	2.0E+05	0.9
R24	0.01	1.99E+62	-15.21	48712.3	6.7E+04	0.8
	0.1	9.71E+54	-12.91	46181.1	7.7E+04	0.8
	1	3.25E+44	-9.53	43147.8	1.1E+05	0.7
	10	2.49E+37	-7.24	41107.3	1.4E+05	0.7
	100	8.58E+21	-2.50	34911.1	1.4E+05	0.7
R25	0.01	1.11E+59	-14.22	47954.9	4.5E+04	0.5
	0.1	1.75E+52	-12.09	45532.0	5.0E+04	0.5
	1	2.89E+40	-8.35	41731.5	6.7E+04	0.4
	10	4.23E+31	-5.55	38806.7	7.9E+04	0.4
	100	1.56E+18	-1.45	33304.3	7.5E+04	0.4
R26	0.01	1.09E+59	-14.22	47946.6	4.5E+04	0.5
	0.1	1.71E+52	-12.09	45523.5	5.0E+04	0.5
	1	2.83E+40	-8.35	41723.6	6.7E+04	0.4
	10	4.16E+31	-5.55	38799.7	7.9E+04	0.4
	100	1.53E+18	-1.45	33296.9	7.5E+04	0.4
R27	0.01	8.73E+66	-16.55	51309.6	7.7E+04	0.9
	0.1	1.33E+59	-14.09	48609.2	9.0E+04	0.9
	1	4.73E+49	-11.00	46140.0	1.4E+05	0.9
	10	5.39E+42	-8.74	44279.1	1.8E+05	0.9
	100	5.32E+25	-3.51	37602.5	1.9E+05	0.9
R28	0.01	3.60E+47	-10.66	47073.1	5.8E+03	0.1
	0.1	3.04E+42	-9.09	45121.9	5.9E+03	0.1
	1	5.23E+28	-4.83	39875.6	6.3E+03	0.04
	10	1.79E+15	-0.70	34484.1	6.2E+03	0.03

	100	2.29E+08	1.39	31610.9	5.9E+03	0.03
R29	0.01	6.33E+66	-16.55	50123.2	1.2E+05	1.4
	0.1	6.56E+58	-14.03	47392.0	1.4E+05	1.4
	1	4.14E+51	-11.61	45982.5	2.3E+05	1.5
	10	6.59E+44	-9.39	44224.6	3.1E+05	1.5
	100	1.19E+28	-4.22	37731.9	3.2E+05	1.5
R30	0.01	5.19E+58	-14.08	48458.2	4.1E+04	0.5
	0.1	1.56E+52	-12.04	46067.1	4.4E+04	0.4
	1	3.16E+39	-8.05	41737.8	5.5E+04	0.4
	10	3.82E+30	-5.24	38677.9	6.3E+04	0.3
	100	2.20E+17	-1.20	33214.0	5.9E+04	0.3
R31	0.01	1.50E+62	-15.12	49549.1	5.5E+04	0.6
	0.1	8.40E+54	-12.85	46956.9	6.1E+04	0.6
	1	1.14E+43	-9.07	43206.8	8.4E+04	0.5
	10	2.10E+35	-6.60	40842.9	1.0E+05	0.5
	100	2.87E+19	-1.74	34407.0	9.9E+04	0.5
R32	0.01	5.63E+64	-15.85	51443.9	4.9E+04	0.6
	0.1	3.15E+57	-13.58	48850.7	5.5E+04	0.6
	1	3.51E+45	-9.77	45066.5	7.5E+04	0.5
	10	5.78E+37	-7.28	42698.6	9.3E+04	0.5
	100	8.18E+21	-2.43	36262.9	8.9E+04	0.4
R33	0.01	1.57E+70	-17.55	51309.8	1.7E+05	2.0
	0.1	5.61E+61	-14.88	48430.0	2.1E+05	2.1
	1	3.88E+60	-14.27	49750.4	3.8E+05	2.4
	10	3.20E+50	-11.04	46647.2	5.1E+05	2.5
	100	6.79E+33	-5.87	40369.8	5.6E+05	2.7
R34	0.01	4.96E+63	-15.67	47180.9	2.0E+05	2.3
	0.1	4.84E+55	-13.16	44383.5	2.3E+05	2.3
	1	5.11E+46	-10.21	42150.4	3.7E+05	2.4
	10	3.00E+43	-9.07	41908.5	5.1E+05	2.5
	100	5.90E+26	-3.93	35328.8	5.1E+05	2.4
R35	0.01	4.72E+50	-11.58	47661.7	1.1E+04	0.1
	0.1	2.74E+45	-9.96	45648.8	1.1E+04	0.1
	1	3.25E+31	-5.65	40393.2	1.2E+04	0.1
	10	1.64E+18	-1.56	35147.3	1.2E+04	0.1
	100	2.89E+10	0.80	31923.7	1.2E+04	0.1
R36	0.01	1.24E+56	-13.26	47924.0	3.3E+04	0.4
	0.1	1.91E+50	-11.45	45736.6	3.4E+04	0.3
	1	1.08E+37	-7.32	40970.3	4.0E+04	0.3
	10	1.46E+27	-4.24	37365.2	4.3E+04	0.2
	100	2.13E+16	-0.95	32848.2	4.1E+04	0.2
R37	0.01	5.09E+64	-15.88	49865.9	9.7E+04	1.1
	0.1	1.64E+57	-13.54	47178.1	1.1E+05	1.1
	1	9.44E+45	-9.93	43760.0	1.5E+05	1.0

	10	1.60E+40	-8.05	42279.0	1.9E+05	0.9
	100	7.25E+23	-3.04	35701.5	1.9E+05	0.9
R38	0.01	2.63E+66	-16.36	51282.6	8.3E+04	1.0
	0.1	6.15E+58	-13.97	48548.1	9.3E+04	0.9
	1	2.32E+47	-10.31	45078.3	1.3E+05	0.8
	10	2.28E+41	-8.35	43533.9	1.7E+05	0.8
	100	2.00E+24	-3.12	36691.9	1.7E+05	0.8
R39	0.01	6.57E+71	-18.03	51484.6	2.5E+05	2.9
	0.1	9.19E+62	-15.24	48466.0	3.0E+05	3.1
	1	2.98E+65	-15.73	51395.4	5.9E+05	3.8
	10	1.28E+54	-12.10	47852.1	8.1E+05	3.9
	100	6.45E+37	-7.02	41894.1	9.4E+05	4.4
R40	0.01	5.15E+66	-16.41	51719.3	8.6E+04	1.0
	0.1	1.75E+59	-14.08	48992.6	9.3E+04	0.9
	1	1.28E+47	-10.22	45130.6	1.3E+05	0.8
	10	2.13E+41	-8.33	43644.9	1.6E+05	0.8
	100	1.49E+24	-3.09	36701.0	1.5E+05	0.7
R41	0.01	4.54E+66	-16.41	51468.2	9.3E+04	1.1
	0.1	1.33E+59	-14.06	48723.4	1.0E+05	1.0
	1	2.27E+47	-10.30	45036.4	1.4E+05	0.9
	10	2.20E+41	-8.35	43418.8	1.8E+05	0.9
	100	2.01E+24	-3.13	36583.4	1.7E+05	0.8
R42	0.01	1.57E+62	-14.99	51826.4	3.3E+04	0.4
	0.1	6.70E+55	-13.02	49428.6	3.4E+04	0.3
	1	1.03E+42	-8.69	44520.4	4.1E+04	0.3
	10	1.33E+33	-5.90	41452.9	4.7E+04	0.2
	100	4.10E+18	-1.48	35436.1	4.3E+04	0.2
R43	0.01	2.02E+53	-12.39	47463.2	2.4E+04	0.3
	0.1	9.04E+47	-10.74	45411.7	2.4E+04	0.2
	1	1.78E+34	-6.48	40316.1	2.7E+04	0.2
	10	3.23E+22	-2.85	35819.3	2.8E+04	0.1
	100	3.12E+13	-0.11	32063.3	2.7E+04	0.1
R44	0.01	5.91E+66	-16.38	52927.5	5.7E+04	0.7
	0.1	2.95E+59	-14.11	50240.1	6.1E+04	0.6
	1	3.53E+46	-10.02	45967.6	8.0E+04	0.5
	10	3.91E+39	-7.78	43921.3	9.9E+04	0.5
	100	2.71E+22	-2.54	36946.7	9.4E+04	0.4
**Eli-s-RI rate rule**	**0.01**	**1.57E+60**	**-14.59**	**47219.6**	**8.6E+04**	**13.9#**
	**0.1**	**4.79E+53**	**-12.53**	**45013.8**	**9.9E+04**	**16.4#**
	**1**	**9.77E+45**	**-9.99**	**43234.3**	**1.6E+05**	**25.0#**
	**10**	**8.58E+39**	**-8.00**	**41735.5**	**2.1E+05**	**34.2#**
	**100**	**7.40E+24**	**-3.36**	**35886.8**	**2.1E+05**	**36.6#**

The uncertainty factor values of the high-pressure limit rule at 800 K for each subclass in our work (The ratios of the largest rate constant of a reaction to the smallest rate constant at 800 K for each subclass).

$^{e}k_{ave}$ is the average rate constants at 800 K for each reaction subclass.

4. Cartesian coordinates for all reactants, transition states and products in the class of concerted elimination

HO_2

O	0.05528900	-0.60941600	0.00000000
H	-0.88463000	-0.87113300	0.00000000
O	0.05528900	0.71830800	0.00000000

R1

C	-1.17139900	0.07933200	0.20266200
C	-1.17939100	1.49025000	-0.39211700
C	0.19886400	1.94982400	-0.88781700
C	1.27105800	1.80442100	0.20051500
C	1.33472800	0.37539400	0.74560700
C	-0.03795200	-0.12535200	1.21269800
H	0.48786300	1.35777100	-1.75894200
H	-1.91296300	1.52677700	-1.20314800
H	-1.54031300	2.16815100	0.39036000
H	2.25467600	2.08021100	-0.18758600
H	2.03879900	0.31292500	1.58352900
H	0.02435000	-1.18088100	1.47867200
H	-0.30682500	0.41577300	2.12613200
H	0.13798900	2.99167400	-1.21517900
H	1.05482300	2.47630000	1.03948500
O	-0.92417800	-0.79432900	-0.99996300
O	-0.74620400	-2.06012700	-0.70430700
C	-2.52669200	-0.33393100	0.76555200
H	-2.77698300	0.28125100	1.63294000
H	-2.50211500	-1.38026600	1.07419400
O	1.86392200	-0.39377400	-0.34316600
O	2.02652400	-1.76462500	0.08304800
H	1.25417200	-2.17852100	-0.34168000
H	-3.30928000	-0.20795100	0.01373900

TS1

C	-2.12705000	1.52276900	-0.03101900
H	-2.97770700	0.56111500	-0.43358200
C	-1.20447200	0.66962600	0.56267100
H	-1.30963800	0.43843600	1.61904800
O	-2.44299500	-1.20347900	0.12975300
O	-3.38657400	-0.63839600	-0.51483300
H	-2.77745900	2.11026600	0.61300700
H	-1.85371000	2.00185400	-0.96967500
C	0.08842900	0.25560500	-0.07839300

C	1.15794000	1.35114100	0.18948100
C	0.59949400	-1.11393300	0.40624300
H	-0.06741100	0.20949900	-1.16485100
C	2.51612900	0.98550700	-0.42739000
H	1.27214100	1.47737700	1.27400600
H	0.80747200	2.31061600	-0.20195400
C	1.95965500	-1.47243400	-0.20839100
H	0.69187900	-1.08697900	1.50054700
H	-0.13655400	-1.88475800	0.17239900
C	3.00947200	-0.38501000	0.05249100
H	3.24906100	1.76205100	-0.18567600
H	2.42277400	0.97446200	-1.52086800
H	2.30177300	-2.43408400	0.18681600
H	1.84156400	-1.60822600	-1.29133300
H	3.95332900	-0.63992600	-0.43985800
H	3.22145000	-0.33633000	1.12845800

P1

C	-1.36277600	1.35554600	0.26413900
C	0.13798600	1.16573500	-0.00225900
C	0.62758700	-0.21704300	0.45403300
C	-0.21913000	-1.33279700	-0.20301900
C	-1.72007900	-1.14460600	0.05769400
C	-2.19961500	0.24445100	-0.38451200
H	0.33029100	1.27788500	-1.07694900
H	0.71068000	1.95044700	0.50242300
H	-1.54024900	1.35520200	1.34756200
H	-1.68533800	2.33575700	-0.10160200
H	-0.02881800	-1.32460400	-1.28387200
H	0.10883000	-2.31168700	0.16333700
H	-2.28953800	-1.92503000	-0.45756900
H	-1.91864600	-1.27099600	1.12993600
H	-2.11844200	0.32293800	-1.47646800
H	-3.25890200	0.37564900	-0.14072200
H	0.45274200	-0.28843300	1.53980300
C	2.09754000	-0.47282100	0.23464200
H	2.44263600	-1.44230500	0.59400600
C	2.98187600	0.33384500	-0.34623200
H	4.01946600	0.03901800	-0.45727600
H	2.71494900	1.31229300	-0.72952300

R2

C	-3.41285600	0.33399500	0.16672700
C	-2.34471300	1.35167900	0.58889800
C	-1.03365100	0.66120100	0.99329000

C	-0.49577600	-0.26073000	-0.11740400
C	-1.57717500	-1.27641000	-0.52962400
C	-2.89020200	-0.59398800	-0.93785900
H	-0.28933600	1.41880600	1.25905600
H	-2.14690000	2.03593700	-0.24604100
H	-2.71200700	1.96859500	1.41532400
H	-3.70249800	-0.26802200	1.03800700
H	-4.31776300	0.85084100	-0.16856800
H	-0.27285000	0.36004200	-0.99520200
H	-1.20581600	-1.89795000	-1.35176100
H	-1.76881200	-1.95585500	0.31290300
H	-2.72170000	-0.00871300	-1.85082300
H	-3.64282300	-1.34881000	-1.18755300
H	-1.20458300	0.06083900	1.89801200
C	0.80215200	-0.98130900	0.28770600
H	1.02646400	-1.76937900	-0.44090900
H	0.65882700	-1.48408100	1.25166000
C	2.03540400	-0.09354100	0.39954800
H	1.83619400	0.81827800	0.96592500
C	3.27077400	-0.79601800	0.93916100
H	3.48397400	-1.70293500	0.36720000
H	3.11667700	-1.07350600	1.98505700
O	2.31548800	0.36407000	-0.97988800
O	3.13525900	1.39305100	-1.00460300
H	4.13323600	-0.13032600	0.88099000

TS2

C	-1.92902300	-0.93304200	0.03967600
C	-3.24042000	-1.00633700	0.49223400
H	-3.41840200	-0.95089300	1.56488400
H	-3.59936800	0.22648200	0.07443100
H	-3.94391700	-1.62434400	-0.06066400
O	-2.19470900	1.13783200	-0.87873900
O	-3.39987200	1.33345400	-0.50927600
H	-1.70417500	-1.29775400	-0.95639500
C	-0.76699900	-0.60774100	0.92917900
H	-0.48350300	-1.54273900	1.43974400
H	-1.10972500	0.06660800	1.72242400
C	0.48659700	-0.02365500	0.25252600
C	1.49949600	0.44024800	1.31586200
C	1.15515900	-1.01587600	-0.71746400
H	0.17576800	0.85685800	-0.32127000
C	2.77486400	1.02258200	0.69177500
H	1.76609400	-0.41432600	1.95402300
H	1.03066100	1.18225000	1.97163400

C	2.42940700	-0.43619500	-1.34918700
H	1.40865600	-1.93364900	-0.16811200
H	0.45859100	-1.30951300	-1.50922700
C	3.42771000	0.03315000	-0.28272800
H	3.48215400	1.30412800	1.47861500
H	2.52326200	1.94578600	0.15448600
H	2.89434200	-1.18176200	-2.00234800
H	2.15860700	0.41256800	-1.98997800
H	4.30318200	0.48853500	-0.75680900
H	3.79367200	-0.83732900	0.27745100

P2

C	1.55267400	1.50267000	-0.22705700
C	0.15319100	1.03615400	0.19891300
C	-0.14843900	-0.39782200	-0.27080500
C	0.95125300	-1.36042400	0.21178500
C	2.35396300	-0.90122600	-0.21149500
C	2.64270200	0.53364400	0.25105000
H	0.07898700	1.06885900	1.29506500
H	-0.60849500	1.72329900	-0.18106500
H	1.59078700	1.57658400	-1.32180000
H	1.74502100	2.50965400	0.15754000
H	0.90775200	-1.42762100	1.30764200
H	0.75470300	-2.36914300	-0.16861700
H	3.10930600	-1.58768100	0.18502000
H	2.43380500	-0.94998000	-1.30532000
H	2.68910000	0.55640900	1.34772900
H	3.62480600	0.85770000	-0.10861300
H	-0.13094100	-0.40037900	-1.37127300
C	-1.54551700	-0.87981800	0.17550900
H	-1.64301300	-1.93881300	-0.10041000
H	-1.61662700	-0.83401900	1.26876500
C	-2.68490900	-0.11732500	-0.43996300
H	-2.71744500	-0.11819800	-1.52947600
C	-3.63192400	0.53551100	0.22783800
H	-3.64301400	0.56668200	1.31344000
H	-4.43268800	1.05844900	-0.28327600

R3

C	-3.59852400	-0.86525500	-0.58467200
C	-2.12351900	-1.27756000	-0.48304000
C	-1.17035800	-0.06748000	-0.45801800
C	-1.58092900	0.90525800	0.66462000
C	-3.05545300	1.32364900	0.56418700
C	-3.98996100	0.10754400	0.53582000

H	-1.97312900	-1.86274200	0.43436000
H	-1.85928900	-1.93723400	-1.31709000
H	-3.77340700	-0.38534200	-1.55648000
H	-4.23846200	-1.75329700	-0.55860100
H	-1.40738400	0.41676500	1.63341900
H	-0.94704300	1.79714000	0.65031300
H	-3.31394700	1.98197200	1.39995300
H	-3.20211800	1.91201000	-0.35100100
H	-3.93472200	-0.41253900	1.50094000
H	-5.02938700	0.42932500	0.41428700
H	-1.28298800	0.46241100	-1.41658300
C	0.29169500	-0.53191800	-0.34319500
H	0.47259800	-1.30229600	-1.10365700
H	0.44022500	-1.01917200	0.62685500
C	1.33622100	0.57841800	-0.51606700
H	1.21708000	1.34906800	0.25224500
H	1.19234800	1.07662700	-1.48194000
C	2.77574100	0.08293000	-0.46369800
H	2.92050500	-0.79101200	-1.10449900
C	3.82334900	1.14478100	-0.75647900
H	3.68933700	2.01188200	-0.10447200
H	4.82254300	0.73579100	-0.59967200
H	3.74267100	1.47531600	-1.79514400
O	2.97588900	-0.41118500	0.91617700
O	4.05098600	-1.16217400	1.02382600
**TS3**			
C	2.64735200	-0.83205900	0.51663000
C	3.79132000	-1.30619800	-0.11208400
H	3.67823300	-1.82441100	-1.06280600
H	4.25164900	-0.09450200	-0.49607300
H	4.61496200	-1.65328200	0.50724600
O	3.15064900	1.35713300	0.12770800
O	4.21552000	1.16914500	-0.54895800
H	2.68820800	-0.60793500	1.57829400
C	1.28558300	-0.84404100	-0.10906500
H	0.89269100	-1.86187100	0.03057000
H	1.38162700	-0.71044700	-1.19290300
C	0.29415400	0.17452000	0.47063600
H	0.21811900	0.02617800	1.55611400
H	0.70218900	1.17800300	0.32448300
C	-1.11631300	0.11446100	-0.14175700
C	-1.85554200	-1.19981400	0.17301000
C	-1.96015100	1.31797800	0.31968400
H	-1.01145100	0.18599400	-1.23520500

C	-3.27668300	-1.22509000	-0.41025800
H	-1.90997900	-1.31940400	1.26419800
H	-1.29263200	-2.05895700	-0.20538700
C	-3.38079500	1.29961700	-0.26220600
H	-2.01936300	1.30840000	1.41684700
H	-1.45282200	2.24924600	0.04583900
C	-4.10169300	-0.01747900	0.05291800
H	-3.77838400	-2.15770600	-0.13211300
H	-3.21528600	-1.22067000	-1.50632800
H	-3.95440000	2.14890500	0.12306300
H	-3.32654500	1.43005400	-1.35084500
H	-5.09105300	-0.03124600	-0.41582000
H	-4.26849600	-0.08794500	1.13577300

P3

C	-2.24313600	1.44554000	0.21793700
C	-0.79352400	1.11330700	-0.16686800
C	-0.37648300	-0.29975800	0.28339700
C	-1.37856200	-1.34099800	-0.25017400
C	-2.82854600	-1.01567400	0.13532300
C	-3.22762400	0.39608500	-0.31427800
H	-0.68372600	1.18049900	-1.25834500
H	-0.12115300	1.86399000	0.25916800
H	-2.32322100	1.49225300	1.31186900
H	-2.50990000	2.44012400	-0.15435600
H	-1.29642100	-1.38209300	-1.34507900
H	-1.10629700	-2.33657200	0.11765500
H	-3.50738900	-1.75855900	-0.29628100
H	-2.93781900	-1.09116100	1.22509400
H	-3.23930500	0.43566300	-1.41137800
H	-4.24551200	0.62726300	0.01640800
H	-0.42501900	-0.32736500	1.38288300
C	1.06027700	-0.66110800	-0.13078400
H	1.23906300	-1.71505700	0.11676800
H	1.15468300	-0.58553300	-1.22128100
C	2.16277100	0.18973300	0.52844900
H	2.06722900	1.23867400	0.23327900
H	2.02417500	0.15412700	1.61804300
C	3.54519900	-0.29545200	0.19307700
H	3.77417500	-1.31722700	0.49502500
C	4.47718700	0.41033800	-0.44067100
H	5.45589800	-0.00252500	-0.65849500
H	4.29518200	1.43143500	-0.76343400

R4

C	-0.63918300	0.36132000	0.26074100
C	0.37096500	1.43660200	-0.15320200
C	1.64143700	0.86320900	-0.79726300
C	2.28647200	-0.21133700	0.08724400
C	1.28413600	-1.32516100	0.41639300
C	0.01102200	-0.76786000	1.06844500
H	1.38691300	0.42864200	-1.76942700
H	-0.12164500	2.14035700	-0.83127300
H	0.63488600	1.99949000	0.74974400
H	3.16716300	-0.62991400	-0.40938100
H	1.73759800	-2.06489900	1.08306600
H	-0.72993600	-1.55740800	1.21430300
H	0.24931000	-0.36217100	2.05877600
H	2.34840100	1.67564700	-0.99126500
H	2.64142900	0.24740000	1.01986600
H	1.01535500	-1.85601700	-0.50297400
O	-1.09281200	-0.19857800	-1.05865700
O	-1.94970100	-1.18707200	-0.93850700
C	-1.86722800	0.94316600	0.94920000
H	-1.58947900	1.36811400	1.91668300
H	-2.61300100	0.16339800	1.11065700
H	-2.31261400	1.73293200	0.33918000

TS4

C	1.43942100	-0.34398100	-1.27748800
C	0.36835100	0.75825600	-1.25707000
C	-0.41227200	0.84468800	0.02905200
C	0.36798800	0.66935900	1.30639300
C	1.43975200	-0.43126700	1.25078100
C	2.30372800	-0.31402000	-0.01086700
H	0.86377400	1.73495400	-1.38159700
H	-0.31776200	0.65672000	-2.10225700
H	0.94769700	-1.31686500	-1.35968200
H	2.06354900	-0.22320500	-2.16826600
H	0.86287900	1.63527500	1.49869300
H	-0.31845500	0.50905900	2.14217400
H	2.06385600	-0.37155600	2.14772000
H	0.94839300	-1.40768700	1.26610200
H	2.87660200	0.62272700	0.02138600
H	3.03691400	-1.12580200	-0.03895600
C	-1.70951400	1.35198300	0.04640600
H	-2.08010000	1.83673800	-0.85517400
H	-2.35167200	0.17611500	0.00528600
H	-2.08047500	1.77286900	0.97935500
O	-1.20773700	-1.37523200	-0.04736500

O	-2.45475300	-1.08844900	-0.03738800

P4

C	-1.02041500	-1.26825400	-0.30049600
C	0.34520200	-1.26902300	0.41644000
C	1.11528300	0.00002900	0.13174000
C	0.34518800	1.26904400	0.41642000
C	-1.02043400	1.26824700	-0.30049400
C	-1.82234900	-0.00002300	0.02202700
H	0.16915400	-1.34293700	1.49924500
H	0.93227200	-2.14535600	0.12873100
H	-0.85326500	-1.32627500	-1.38278200
H	-1.58888500	-2.16080400	-0.01992200
H	0.16913000	1.34295000	1.49922400
H	0.93224000	2.14540300	0.12873400
H	-1.58892900	2.16077500	-0.01990200
H	-0.85330900	1.32627500	-1.38278200
H	-2.08568500	-0.00002600	1.08795900
H	-2.76687200	-0.00001800	-0.53152500
C	2.35263900	-0.00000600	-0.36305800
H	2.88176800	0.92444000	-0.57124500
H	2.88169200	-0.92450600	-0.57120700

R5

C	-3.06673700	0.47814900	-0.11574200
C	-1.94573500	1.51942800	-0.00292100
C	-0.71500600	0.95109400	0.71957800
C	-0.19566500	-0.33750900	0.05396600
C	-1.33039500	-1.37208300	-0.05815400
C	-2.56309100	-0.81042700	-0.77982100
H	0.07100600	1.71233200	0.75293300
H	-1.65282800	1.84629600	-1.00874000
H	-2.30594300	2.41103600	0.52017900
H	-3.44435200	0.24365200	0.88811700
H	-3.91124100	0.88962900	-0.67772300
H	0.12610200	-0.08557300	-0.96518100
H	-0.96514900	-2.26435900	-0.57828000
H	-1.61807700	-1.69636300	0.95176100
H	-2.30237700	-0.59943800	-1.82481200
H	-3.35842400	-1.56223900	-0.80367400
H	-0.97929300	0.72830900	1.76297300
C	1.01257400	-0.94265300	0.79117600
H	1.23164900	-1.93415900	0.37934500
H	0.76084200	-1.09690100	1.84778400
C	2.28754800	-0.12007400	0.73882900

H	2.15554700	0.91605600	1.05375400
O	2.74076400	-0.08918200	-0.65290100
O	3.78417400	0.69767000	-0.81003500
H	3.09207100	-0.57172800	1.32359200

TS5

H	1.91185900	-0.55174000	0.46339700
C	2.10880900	1.36875600	0.00661700
H	2.02143100	1.65022200	-1.03621900
H	2.87887300	1.87761900	0.57162700
O	3.54104500	-0.23160800	-0.53210800
O	2.92894600	-1.20678000	0.00160800
C	1.17103000	0.54782000	0.61989300
H	1.13466800	0.59195800	1.71116300
C	-0.16182800	0.21730200	-0.05164800
C	-0.71913700	-1.14285200	0.41037200
C	-1.20080900	1.33210200	0.19166700
H	0.01867400	0.15569900	-1.13439500
C	-2.07927400	-1.46265200	-0.22564000
H	-0.82542700	-1.12714800	1.50385100
H	-0.00139100	-1.93653700	0.17880500
C	-2.56087000	1.01275100	-0.44527300
H	-1.32857400	1.46302800	1.27485400
H	-0.81379000	2.28246400	-0.19074200
C	-3.10002200	-0.34474300	0.02451600
H	-2.45636300	-2.41541900	0.15957400
H	-1.94834700	-1.59589600	-1.30738700
H	-3.27668100	1.80820900	-0.21383000
H	-2.45371400	0.99965600	-1.53773200
H	-4.04433400	-0.57323200	-0.47993600
H	-3.32419500	-0.29268200	1.09793900

P5

C	-1.36277600	1.35554600	0.26413900
C	0.13798600	1.16573500	-0.00225900
C	0.62758700	-0.21704300	0.45403300
C	-0.21913000	-1.33279700	-0.20301900
C	-1.72007900	-1.14460600	0.05769400
C	-2.19961500	0.24445100	-0.38451200
H	0.33029100	1.27788500	-1.07694900
H	0.71068000	1.95044700	0.50242300
H	-1.54024900	1.35520200	1.34756200
H	-1.68533800	2.33575700	-0.10160200
H	-0.02881800	-1.32460400	-1.28387200
H	0.10883000	-2.31168700	0.16333700
H	-2.28953800	-1.92503000	-0.45756900

H	-1.91864600	-1.27099600	1.12993600
H	-2.11844200	0.32293800	-1.47646800
H	-3.25890200	0.37564900	-0.14072200
H	0.45274200	-0.28843300	1.53980300
C	2.09754000	-0.47282100	0.23464200
H	2.44263600	-1.44230500	0.59400600
C	2.98187600	0.33384500	-0.34623200
H	4.01946600	0.03901800	-0.45727600
H	2.71494900	1.31229300	-0.72952300

R6

C	3.22902700	-1.08884800	0.05571300
C	1.73050100	-1.36403800	-0.13066300
C	0.84781900	-0.22963000	0.42279100
C	1.27151600	1.11763400	-0.19329100
C	2.77010800	1.39969000	-0.00697200
C	3.63587300	0.25836900	-0.55588100
H	1.51539400	-1.48840000	-1.20077200
H	1.45981600	-2.31030600	0.35088900
H	3.46477100	-1.08378900	1.12793400
H	3.81647100	-1.90007700	-0.38646800
H	1.03574300	1.10210800	-1.26631600
H	0.68937200	1.93722400	0.23930100
H	3.03427800	2.34466200	-0.49264600
H	2.98208500	1.53081000	1.06220000
H	3.52039400	0.20966800	-1.64642700
H	4.69496200	0.45869700	-0.36387200
H	1.02550300	-0.17170200	1.50786100
C	-0.64183600	-0.54575900	0.20644600
H	-0.83402700	-1.57053700	0.54928900
H	-0.86164000	-0.53643400	-0.86712700
C	-1.61281700	0.39652000	0.93054700
H	-1.48463600	1.42826200	0.58998000
H	-1.39995300	0.39488700	2.00628100
C	-3.07136800	0.01369300	0.75663400
H	-3.26544900	-1.03578000	0.99055000
O	-3.42135500	0.21082500	-0.65062700
O	-4.65397600	-0.17341600	-0.90612700
H	-3.74338600	0.64564100	1.34142600

TS6

C	-1.85651700	0.32443200	0.89393300
H	-2.00500700	-0.65228400	-0.00724400
C	-3.15284800	0.79246700	0.72013500
H	-3.97471100	0.43094100	1.32440600

H	-3.37021500	1.64034300	0.08137900
O	-3.72079600	-0.60093700	-0.90137700
O	-2.64990200	-1.28076300	-0.93736400
C	-0.65670100	1.13281200	0.40085900
H	-0.98435000	1.76310200	-0.43423200
H	-0.33859200	1.82007000	1.19607600
H	-1.68881800	-0.27545200	1.78879400
C	0.56231800	0.30998700	-0.05436700
C	1.64079000	1.22707000	-0.65975900
C	1.16614100	-0.54074600	1.07914800
H	0.22837200	-0.37593900	-0.84640700
C	2.87954900	0.44796100	-1.12379100
H	1.94130100	1.96682400	0.09510200
H	1.21729700	1.79248300	-1.49730000
C	2.40523700	-1.32452100	0.62061500
H	1.44352800	0.12378100	1.90962100
H	0.42008400	-1.23756100	1.47383300
C	3.46804700	-0.40169700	0.01069800
H	3.63411600	1.13975100	-1.51206100
H	2.60056400	-0.20810800	-1.95837700
H	2.82555400	-1.88430100	1.46241100
H	2.10186900	-2.06845400	-0.12709800
H	4.31586500	-0.98959800	-0.35558600
H	3.86263500	0.26141400	0.79178900

P6

C	1.55267400	1.50267000	-0.22705700
C	0.15319100	1.03615400	0.19891300
C	-0.14843900	-0.39782200	-0.27080500
C	0.95125300	-1.36042400	0.21178500
C	2.35396300	-0.90122600	-0.21149500
C	2.64270200	0.53364400	0.25105000
H	0.07898700	1.06885900	1.29506500
H	-0.60849500	1.72329900	-0.18106500
H	1.59078700	1.57658400	-1.32180000
H	1.74502100	2.50965400	0.15754000
H	0.90775200	-1.42762100	1.30764200
H	0.75470300	-2.36914300	-0.16861700
H	3.10930600	-1.58768100	0.18502000
H	2.43380500	-0.94998000	-1.30532000
H	2.68910000	0.55640900	1.34772900
H	3.62480600	0.85770000	-0.10861300
H	-0.13094100	-0.40037900	-1.37127300
C	-1.54551700	-0.87981800	0.17550900
H	-1.64301300	-1.93881300	-0.10041000

H	-1.61662700	-0.83401900	1.26876500
C	-2.68490900	-0.11732500	-0.43996300
H	-2.71744500	-0.11819800	-1.52947600
C	-3.63192400	0.53551100	0.22783800
H	-3.64301400	0.56668200	1.31344000
H	-4.43268800	1.05844900	-0.28327600

R7

C	-3.78959100	-0.59926700	-0.84189200
C	-2.49808600	-1.26875900	-0.35089100
C	-1.33273900	-0.27099300	-0.20886400
C	-1.75302000	0.90730300	0.68924500
C	-3.04317000	1.58315500	0.20040300
C	-4.19207200	0.57634200	0.05783100
H	-2.68313700	-1.73794600	0.62543800
H	-2.21036900	-2.07674800	-1.03272900
H	-3.63934900	-0.23432500	-1.86620400
H	-4.59860500	-1.33545300	-0.89062800
H	-1.90546600	0.53292900	1.71137400
H	-0.95144100	1.64980800	0.74614000
H	-3.32632900	2.38877100	0.88564900
H	-2.85501000	2.05542000	-0.77258100
H	-4.46325500	0.19478900	1.05094300
H	-5.08411700	1.07101400	-0.33997900
H	-1.11866800	0.13220900	-1.21021800
C	-0.06180800	-0.98079900	0.29009000
H	0.10074300	-1.87432400	-0.32594200
H	-0.23444900	-1.34516300	1.31224500
C	1.21423800	-0.13064100	0.26067100
H	1.10417000	0.74117800	0.91482300
H	1.37042800	0.25826800	-0.75121900
C	2.45844800	-0.91601800	0.69487500
H	2.33037000	-1.28236000	1.72024500
H	2.58547300	-1.80252400	0.06364300
C	3.74047000	-0.10483400	0.65859100
H	3.65734200	0.84461000	1.19287200
H	4.59999200	-0.66056100	1.03979800
O	4.02848400	0.21501200	-0.74029700
O	5.07972400	0.99842200	-0.85645600

TS7

C	2.58682200	-0.78246000	0.56007800
H	2.94874900	0.50117500	0.45355200
C	3.72186100	-1.24150000	-0.09464000
H	4.62267800	-1.50500900	0.44413700

H	3.70774200	-1.49481800	-1.14804400
O	4.57087400	0.74786400	-0.57207000
O	3.69831400	1.45894100	0.01394100
C	1.20710100	-0.85056700	-0.09294500
H	1.31361300	-0.68387800	-1.17153700
H	0.81366200	-1.86565600	0.02542200
H	2.58771800	-0.88200100	1.64741400
C	0.21094300	0.16510500	0.48405300
H	0.12387700	0.01153700	1.56821700
H	0.62266600	1.17306500	0.35244000
C	-1.19561700	0.12586500	-0.13878500
C	-1.94865600	-1.18403800	0.16191700
C	-2.03216300	1.33344500	0.32519900
H	-1.08179800	0.20322300	-1.23090100
C	-3.36600200	-1.19213600	-0.43100900
H	-2.01157200	-1.31032800	1.25190100
H	-1.38942400	-2.04439100	-0.21815600
C	-3.44897400	1.33216200	-0.26631900
H	-2.09891400	1.31692400	1.42189200
H	-1.51533100	2.26264100	0.06120400
C	-4.18364800	0.01942400	0.03481900
H	-3.87790100	-2.12213700	-0.16273100
H	-3.29732500	-1.18094500	-1.52662200
H	-4.01757300	2.18395800	0.12090500
H	-3.38607500	1.46951200	-1.35364300
H	-5.16988400	0.01772700	-0.44066700
H	-4.35841700	-0.05683900	1.11603400

P7

C	-2.24313600	1.44554000	0.21793700
C	-0.79352400	1.11330700	-0.16686800
C	-0.37648300	-0.29975800	0.28339700
C	-1.37856200	-1.34099800	-0.25017400
C	-2.82854600	-1.01567400	0.13532300
C	-3.22762400	0.39608500	-0.31427800
H	-0.68372600	1.18049900	-1.25834500
H	-0.12115300	1.86399000	0.25916800
H	-2.32322100	1.49225300	1.31186900
H	-2.50990000	2.44012400	-0.15435600
H	-1.29642100	-1.38209300	-1.34507900
H	-1.10629700	-2.33657200	0.11765500
H	-3.50738900	-1.75855900	-0.29628100
H	-2.93781900	-1.09116100	1.22509400
H	-3.23930500	0.43566300	-1.41137800
H	-4.24551200	0.62726300	0.01640800

H	-0.42501900	-0.32736500	1.38288300
C	1.06027700	-0.66110800	-0.13078400
H	1.23906300	-1.71505700	0.11676800
H	1.15468300	-0.58553300	-1.22128100
C	2.16277100	0.18973300	0.52844900
H	2.06722900	1.23867400	0.23327900
H	2.02417500	0.15412700	1.61804300
C	3.54519900	-0.29545200	0.19307700
H	3.77417500	-1.31722700	0.49502500
C	4.47718700	0.41033800	-0.44067100
H	5.45589800	-0.00252500	-0.65849500
H	4.29518200	1.43143500	-0.76343400

R8

C	-3.41285600	0.33399500	0.16672700
C	-2.34471300	1.35167900	0.58889800
C	-1.03365100	0.66120100	0.99329000
C	-0.49577600	-0.26073000	-0.11740400
C	-1.57717500	-1.27641000	-0.52962400
C	-2.89020200	-0.59398800	-0.93785900
H	-0.28933600	1.41880600	1.25905600
H	-2.14690000	2.03593700	-0.24604100
H	-2.71200700	1.96859500	1.41532400
H	-3.70249800	-0.26802200	1.03800700
H	-4.31776300	0.85084100	-0.16856800
H	-0.27285000	0.36004200	-0.99520200
H	-1.20581600	-1.89795000	-1.35176100
H	-1.76881200	-1.95585500	0.31290300
H	-2.72170000	-0.00871300	-1.85082300
H	-3.64282300	-1.34881000	-1.18755300
H	-1.20458300	0.06083900	1.89801200
C	0.80215200	-0.98130900	0.28770600
H	1.02646400	-1.76937900	-0.44090900
H	0.65882700	-1.48408100	1.25166000
C	2.03540400	-0.09354100	0.39954800
H	1.83619400	0.81827800	0.96592500
C	3.27077400	-0.79601800	0.93916100
H	3.48397400	-1.70293500	0.36720000
H	3.11667700	-1.07350600	1.98505700
O	2.31548800	0.36407000	-0.97988800
O	3.13525900	1.39305100	-1.00460300
H	4.13323600	-0.13032600	0.88099000

TS8

C	3.12657800	-1.58276200	0.38597400

C	1.95940300	-0.88294100	-0.23035900
H	2.93260300	-2.66309300	0.41943500
H	3.30212700	-1.24547000	1.40996600
C	0.94500000	-0.26298200	0.49387000
H	0.96220400	-0.43123300	1.57454100
H	1.50369900	0.94728100	0.42888800
O	3.06556400	1.03135000	-0.70339000
O	2.34905400	1.81994900	-0.00481900
H	1.82562600	-1.01993400	-1.29951900
H	4.03834800	-1.43168300	-0.19558700
C	-0.44939200	-0.06085400	-0.10049700
C	-1.29933100	-1.34437800	0.00633200
C	-1.18833600	1.12388300	0.55138200
H	-0.32477100	0.17070100	-1.16797800
C	-2.71330800	-1.15810300	-0.56217500
H	-1.36752700	-1.63440200	1.06370100
H	-0.78846300	-2.16596600	-0.50719500
C	-2.60232400	1.31178900	-0.01565900
H	-1.25311400	0.94714000	1.63385900
H	-0.60638500	2.04164800	0.41971900
C	-3.43428100	0.02695400	0.09410300
H	-3.29294700	-2.07752300	-0.43015500
H	-2.64671800	-0.98515900	-1.64414800
H	-3.10506500	2.13552300	0.50117200
H	-2.53057700	1.60589200	-1.07080700
H	-4.41901000	0.17216400	-0.36163000
H	-3.61103000	-0.19990600	1.15358400

P8

C	1.91360300	1.35313600	-0.22318100
C	0.39994400	1.16865800	-0.03833000
C	-0.07093300	-0.21184800	-0.52224700
C	0.73680800	-1.32957400	0.17903400
C	2.25052000	-1.14803300	0.00026000
C	2.71099000	0.23901300	0.46841900
H	0.15113400	1.28106900	1.02482100
H	-0.14088900	1.95687100	-0.57204800
H	2.14934700	1.35330200	-1.29535200
H	2.21930300	2.33191700	0.16066800
H	0.48789800	-1.32130400	1.24792100
H	0.42562200	-2.30722600	-0.20488000
H	2.78827900	-1.93099800	0.54497500
H	2.50644900	-1.27463000	-1.05982000
H	2.57173600	0.31727300	1.55454800
H	3.78229900	0.36685100	0.28185500

H	0.16691700	-0.28087400	-1.59628100
C	-1.55233200	-0.46393000	-0.38644300
H	-1.88068300	-1.43172700	-0.76901700
C	-2.47033100	0.34151600	0.14591500
H	-2.17092600	1.31177200	0.53565300
C	-3.93308100	0.01859800	0.25632400
H	-4.26550000	0.03613800	1.30053400
H	-4.54359400	0.75529600	-0.27831600
H	-4.15851500	-0.96894200	-0.15377200

R9

C	-2.60263000	0.77234500	0.85352200
C	-1.10604100	1.06220400	0.67043200
C	-0.28944400	-0.23021200	0.47784400
C	-0.85850000	-1.06688800	-0.68518300
C	-2.35780200	-1.34823200	-0.50402800
C	-3.16179800	-0.05646600	-0.31005000
H	-0.96622300	1.71051700	-0.20166200
H	-0.72122100	1.61771000	1.53231700
H	-2.75459900	0.22418000	1.79260400
H	-3.15323900	1.71325300	0.95051600
H	-0.69974300	-0.52292900	-1.62311200
H	-0.31884800	-2.01410700	-0.77543600
H	-2.73540200	-1.90555400	-1.36718900
H	-2.50062400	-1.99673900	0.37045100
H	-3.11765500	0.53855500	-1.23114900
H	-4.21767100	-0.28921100	-0.13859400
H	-0.38596300	-0.82335500	1.39963600
C	1.21443000	0.06418400	0.34757500
H	1.52489800	0.74939100	1.14039900
C	2.12884400	-1.15789900	0.30093200
H	1.94113800	-1.70927300	-0.62483900
H	1.84147600	-1.82171800	1.12360600
C	3.61485900	-0.80294400	0.40305800
H	3.91257000	-0.13018300	-0.40417700
H	3.84001300	-0.30742500	1.35212300
O	1.45411800	0.80796500	-0.90602300
O	1.64389800	2.09832500	-0.71385200
H	4.23545000	-1.69997700	0.33888600

TS9

C	2.03603800	-0.91409300	-0.31404900
H	2.58870600	0.22761600	-0.68723900
C	1.00660300	-0.31529600	0.40871400
H	1.13078000	-0.18055300	1.48059400

O	1.84965500	1.80505800	0.15194500
O	2.79475400	1.50617200	-0.64831000
C	-0.37269300	-0.08300000	-0.13641700
C	-1.20972400	-1.38292600	0.01914800
C	-1.10434100	1.09879200	0.52780400
H	-0.28429600	0.12008200	-1.21256100
C	-2.63739600	-1.20997700	-0.51962600
H	-1.25145200	-1.65319600	1.08235700
H	-0.70741700	-2.20821500	-0.49432100
C	-2.53229600	1.26613000	-0.00899000
H	-1.14212500	0.92442200	1.61173400
H	-0.53263600	2.01581400	0.37694200
C	-3.35319800	-0.02178300	0.13543300
H	-3.20432200	-2.13293100	-0.36023200
H	-2.59475700	-1.05346800	-1.60503400
H	-3.02766800	2.09281600	0.50994400
H	-2.48771900	1.54945200	-1.06862400
H	-4.34802500	0.10857900	-0.30233000
H	-3.50648900	-0.23556900	1.20118800
H	1.75322400	-1.32179900	-1.28729800
C	3.17474500	-1.64502600	0.38919600
H	4.07288500	-1.67664900	-0.23284000
H	3.43868700	-1.14545000	1.32509800
H	2.90094100	-2.67771700	0.62626100
**P9**			
C	1.91360300	1.35313600	-0.22318100
C	0.39994400	1.16865800	-0.03833000
C	-0.07093300	-0.21184800	-0.52224700
C	0.73680800	-1.32957400	0.17903400
C	2.25052000	-1.14803300	0.00026000
C	2.71099000	0.23901300	0.46841900
H	0.15113400	1.28106900	1.02482100
H	-0.14088900	1.95687100	-0.57204800
H	2.14934700	1.35330200	-1.29535200
H	2.21930300	2.33191700	0.16066800
H	0.48789800	-1.32130400	1.24792100
H	0.42562200	-2.30722600	-0.20488000
H	2.78827900	-1.93099800	0.54497500
H	2.50644900	-1.27463000	-1.05982000
H	2.57173600	0.31727300	1.55454800
H	3.78229900	0.36685100	0.28185500
H	0.16691700	-0.28087400	-1.59628100
C	-1.55233200	-0.46393000	-0.38644300
H	-1.88068300	-1.43172700	-0.76901700

C	-2.47033100	0.34151600	0.14591500
H	-2.17092600	1.31177200	0.53565300
C	-3.93308100	0.01859800	0.25632400
H	-4.26550000	0.03613800	1.30053400
H	-4.54359400	0.75529600	-0.27831600
H	-4.15851500	-0.96894200	-0.15377200

R10

C	-3.59852400	-0.86525500	-0.58467200
C	-2.12351900	-1.27756000	-0.48304000
C	-1.17035800	-0.06748000	-0.45801800
C	-1.58092900	0.90525800	0.66462000
C	-3.05545300	1.32364900	0.56418700
C	-3.98996100	0.10754400	0.53582000
H	-1.97312900	-1.86274200	0.43436000
H	-1.85928900	-1.93723400	-1.31709000
H	-3.77340700	-0.38534200	-1.55648000
H	-4.23846200	-1.75329700	-0.55860100
H	-1.40738400	0.41676500	1.63341900
H	-0.94704300	1.79714000	0.65031300
H	-3.31394700	1.98197200	1.39995300
H	-3.20211800	1.91201000	-0.35100100
H	-3.93472200	-0.41253900	1.50094000
H	-5.02938700	0.42932500	0.41428700
H	-1.28298800	0.46241100	-1.41658300
C	0.29169500	-0.53191800	-0.34319500
H	0.47259800	-1.30229600	-1.10365700
H	0.44022500	-1.01917200	0.62685500
C	1.33622100	0.57841800	-0.51606700
H	1.21708000	1.34906800	0.25224500
H	1.19234800	1.07662700	-1.48194000
C	2.77574100	0.08293000	-0.46369800
H	2.92050500	-0.79101200	-1.10449900
C	3.82334900	1.14478100	-0.75647900
H	3.68933700	2.01188200	-0.10447200
H	4.82254300	0.73579100	-0.59967200
H	3.74267100	1.47531600	-1.79514400
O	2.97588900	-0.41118500	0.91617700
O	4.05098600	-1.16217400	1.02382600

TS10

C	4.05534000	-0.92317100	0.73106400
C	2.83292800	-0.81820000	-0.12148900
H	4.29204700	-1.98095600	0.90731100
H	3.90817300	-0.44968200	1.70439500

C	1.55092900	-0.60254800	0.37308000
H	1.45209900	-0.64887700	1.46075100
H	1.60799200	0.72672600	0.18600000
O	3.18483700	1.32569000	-0.74672300
O	2.11051800	1.81488800	-0.26586700
H	2.94900200	-1.09879100	-1.16446100
H	4.92292300	-0.47013200	0.24694200
C	0.31849000	-1.06062500	-0.41071900
H	0.39969200	-0.71330000	-1.44858700
H	0.31446800	-2.15667700	-0.45553600
C	-1.03131800	-0.60619800	0.17127200
C	-1.21946300	0.92241300	0.15416800
C	-2.19451400	-1.29407200	-0.56813600
H	-1.06825400	-0.93469100	1.22145700
C	-2.59377000	1.34747800	0.69303400
H	-1.10543300	1.28120600	-0.87773500
H	-0.43418300	1.41217600	0.73700900
C	-3.56815200	-0.87347800	-0.02796500
H	-2.13352900	-1.03968400	-1.63528000
H	-2.08201800	-2.38212600	-0.50278800
C	-3.73941000	0.65114000	-0.05234700
H	-2.69961400	2.43483100	0.62351700
H	-2.65530900	1.09781300	1.76040300
H	-4.36198000	-1.35535300	-0.60806500
H	-3.67634800	-1.23202600	1.00401800
H	-4.70342400	0.93237500	0.38393000
H	-3.75549200	0.99611000	-1.09433500

P10

C	-2.10950200	1.51949900	0.19155000
C	-0.80887600	1.05068600	-0.47823600
C	-0.34279200	-0.31966900	0.04682800
C	-1.47555100	-1.35371000	-0.08916500
C	-2.77662200	-0.89143100	0.58207900
C	-3.22712300	0.47721200	0.05412300
H	-0.96675900	0.97781100	-1.56364800
H	-0.02685700	1.80073500	-0.32645000
H	-1.91872000	1.70476600	1.25661800
H	-2.42712200	2.47586000	-0.23695300
H	-1.66390300	-1.53436600	-1.15667400
H	-1.15382900	-2.31169200	0.33455700
H	-3.56413800	-1.63661600	0.42811400
H	-2.61902300	-0.82526800	1.66645500
H	-3.50393100	0.38235100	-1.00409600
H	-4.12613700	0.81149600	0.58237200

H	-0.11462100	-0.20917000	1.11659000
C	0.94237400	-0.82388800	-0.64895500
H	1.15665900	-1.83867300	-0.29519700
H	0.73740000	-0.90672200	-1.72589100
C	2.15878900	0.03472900	-0.43631200
H	2.13729500	1.03222200	-0.87344500
C	3.24500900	-0.33618600	0.24028000
H	3.27373600	-1.33652800	0.67255900
C	4.46279700	0.51271700	0.46247000
H	5.35833000	0.03919800	0.04386400
H	4.35432100	1.49769700	0.00177300
H	4.65628400	0.65715400	1.53148200

R11

C	3.74288000	0.29895300	-0.36767000
C	2.63942200	1.29038000	-0.75992300
C	1.30390200	0.57935400	-1.02443300
C	0.85967800	-0.28496500	0.17076100
C	1.97706700	-1.27239700	0.55587600
C	3.31316200	-0.56631100	0.82419600
H	0.53802500	1.32285400	-1.26671800
H	2.50367200	2.01964100	0.04890400
H	2.93869700	1.86187200	-1.64450700
H	3.96604800	-0.35087700	-1.22411500
H	4.66863300	0.83468700	-0.13423400
H	0.69794600	0.38145500	1.02824400
H	1.67276800	-1.84673100	1.43785900
H	2.10864400	-1.99946200	-0.25800900
H	3.21143800	0.06910600	1.71324400
H	4.08709600	-1.30483500	1.05704000
H	1.40636200	-0.06536500	-1.90884900
C	-0.45713300	-1.03524200	-0.09319600
H	-0.62285300	-1.76888100	0.70465200
H	-0.37461700	-1.60672800	-1.02570900
C	-1.70358800	-0.15979600	-0.18615400
H	-1.56739000	0.68821600	-0.85922100
C	-2.97472000	-0.93338100	-0.53520500
H	-3.12316500	-1.71324100	0.21987200
H	-2.79041800	-1.45022300	-1.48378000
C	-4.22881800	-0.06366100	-0.65112000
H	-4.11785500	0.69125000	-1.43460300
H	-5.09940400	-0.67683000	-0.89689800
H	-4.43827200	0.45843800	0.28435500
O	-1.88618200	0.44113200	1.15128400

O	-2.17587800	1.72612300	1.10417500

TS11

C	-2.94125400	-0.43545200	0.63306500
H	-3.10870600	0.77443100	0.11399400
C	-1.63481800	-0.57277300	0.17157200
H	-1.47047700	-1.09690300	-0.76436200
O	-1.68124700	1.36669700	-1.04664800
O	-2.81913100	1.77896900	-0.64645500
H	-3.04459600	-0.20756200	1.69642900
C	-4.08166300	-1.24723200	0.02970400
H	-5.04497600	-0.75234700	0.17689200
H	-3.93984800	-1.38048700	-1.04614600
H	-4.14860800	-2.24061800	0.48409800
C	-0.43001900	-0.25550400	1.00484400
H	-0.68266800	0.56024100	1.69214200
H	-0.24183200	-1.13212200	1.64681400
C	0.87064100	0.08217600	0.25340300
C	1.94702200	0.56712400	1.24231300
C	1.40532300	-1.10462000	-0.57014700
H	0.65344400	0.90528000	-0.43685500
C	3.27152300	0.90464300	0.54401300
H	2.12311200	-0.21683300	1.99251300
H	1.57822800	1.44228700	1.78865600
C	2.72873700	-0.77079100	-1.27473800
H	1.55853600	-1.96214600	0.10053600
H	0.66717000	-1.41970000	-1.31449800
C	3.79063700	-0.27963500	-0.28219000
H	4.01865500	1.20820300	1.28449400
H	3.12006800	1.76735400	-0.11712400
H	3.09347000	-1.64841800	-1.81826800
H	2.54885100	0.00819200	-2.02641300
H	4.70630900	-0.00042600	-0.81334200
H	4.06010800	-1.10137900	0.39422300

P11

C	-2.10950200	1.51949900	0.19155000
C	-0.80887600	1.05068600	-0.47823600
C	-0.34279200	-0.31966900	0.04682800
C	-1.47555100	-1.35371000	-0.08916500
C	-2.77662200	-0.89143100	0.58207900
C	-3.22712300	0.47721200	0.05412300
H	-0.96675900	0.97781100	-1.56364800
H	-0.02685700	1.80073500	-0.32645000
H	-1.91872000	1.70476600	1.25661800
H	-2.42712200	2.47586000	-0.23695300

H	-1.66390300	-1.53436600	-1.15667400
H	-1.15382900	-2.31169200	0.33455700
H	-3.56413800	-1.63661600	0.42811400
H	-2.61902300	-0.82526800	1.66645500
H	-3.50393100	0.38235100	-1.00409600
H	-4.12613700	0.81149600	0.58237200
H	-0.11462100	-0.20917000	1.11659000
C	0.94237400	-0.82388800	-0.64895500
H	1.15665900	-1.83867300	-0.29519700
H	0.73740000	-0.90672200	-1.72589100
C	2.15878900	0.03472900	-0.43631200
H	2.13729500	1.03222200	-0.87344500
C	3.24500900	-0.33618600	0.24028000
H	3.27373600	-1.33652800	0.67255900
C	4.46279700	0.51271700	0.46247000
H	5.35833000	0.03919800	0.04386400
H	4.35432100	1.49769700	0.00177300
H	4.65628400	0.65715400	1.53148200

R12

C	3.74288000	0.29895300	-0.36767000
C	2.63942200	1.29038000	-0.75992300
C	1.30390200	0.57935400	-1.02443300
C	0.85967800	-0.28496500	0.17076100
C	1.97706700	-1.27239700	0.55587600
C	3.31316200	-0.56631100	0.82419600
H	0.53802500	1.32285400	-1.26671800
H	2.50367200	2.01964100	0.04890400
H	2.93869700	1.86187200	-1.64450700
H	3.96604800	-0.35087700	-1.22411500
H	4.66863300	0.83468700	-0.13423400
H	0.69794600	0.38145500	1.02824400
H	1.67276800	-1.84673100	1.43785900
H	2.10864400	-1.99946200	-0.25800900
H	3.21143800	0.06910600	1.71324400
H	4.08709600	-1.30483500	1.05704000
H	1.40636200	-0.06536500	-1.90884900
C	-0.45713300	-1.03524200	-0.09319600
H	-0.62285300	-1.76888100	0.70465200
H	-0.37461700	-1.60672800	-1.02570900
C	-1.70358800	-0.15979600	-0.18615400
H	-1.56739000	0.68821600	-0.85922100
C	-2.97472000	-0.93338100	-0.53520500
H	-3.12316500	-1.71324100	0.21987200

H	-2.79041800	-1.45022300	-1.48378000
C	-4.22881800	-0.06366100	-0.65112000
H	-4.11785500	0.69125000	-1.43460300
H	-5.09940400	-0.67683000	-0.89689800
H	-4.43827200	0.45843800	0.28435500
O	-1.88618200	0.44113200	1.15128400
O	-2.17587800	1.72612300	1.10417500

TS12

C	2.86744400	-1.11659500	0.58132800
C	1.65961300	-0.55331300	-0.10552300
H	2.61538800	-2.14933200	0.86836500
H	3.03263700	-0.58302400	1.52353900
C	0.55969500	-0.02016500	0.55964000
H	0.55924800	-0.13861200	1.64733500
H	0.97795800	1.24032000	0.45820700
O	2.55040000	1.48808900	-0.63748700
O	1.71900900	2.19836400	0.01791100
H	1.58018900	-0.74369700	-1.17306400
C	-0.82907500	-0.00992300	-0.08174400
C	-1.52222800	-1.38004000	0.06693300
C	-1.72219300	1.10750300	0.49122400
H	-0.69752900	0.18505700	-1.15565700
C	-2.93130500	-1.39152100	-0.54266700
H	-1.58636100	-1.62768000	1.13528400
H	-0.90175000	-2.15600500	-0.39401300
C	-3.13188200	1.09681100	-0.11747000
H	-1.79712400	0.97686800	1.57963300
H	-1.24936700	2.08093800	0.32625000
C	-3.80627400	-0.27246700	0.03794700
H	-3.40170100	-2.36663500	-0.37905600
H	-2.85508400	-1.26202500	-1.63012900
H	-3.74410800	1.87737500	0.34537000
H	-3.06476500	1.34683500	-1.18419000
H	-4.78821200	-0.26897900	-0.44606800
H	-3.98427000	-0.46896600	1.10327400
C	4.14575200	-1.13036800	-0.26243800
H	4.95951000	-1.62012700	0.27807600
H	3.99252500	-1.67685300	-1.19784900
H	4.46026600	-0.11660600	-0.51206900

P12

C	2.29218500	1.45588200	-0.18816500
C	0.80994300	1.12680700	0.04404500
C	0.44323600	-0.26589600	-0.49198900
C	1.37509400	-1.33838800	0.12054100

C	2.85794000	-1.01262200	-0.10588300
C	3.21343000	0.38658300	0.41473100
H	0.59732100	1.16521900	1.12021100
H	0.17927200	1.88734900	-0.42755600
H	2.48153800	1.52864200	-1.26705100
H	2.52619500	2.43878000	0.23367600
H	1.17169900	-1.40318000	1.19708300
H	1.13546000	-2.32080900	-0.30069200
H	3.48525700	-1.76880100	0.37742600
H	3.07896900	-1.06515300	-1.17997300
H	3.11394300	0.40015100	1.50794800
H	4.26039600	0.61936600	0.19447000
H	0.64114000	-0.26213400	-1.57625700
C	-1.00317400	-0.65682000	-0.31472200
H	-1.26082700	-1.62939600	-0.73735700
C	-1.96563500	0.03209100	0.29761400
H	-1.74011000	1.00633700	0.72781300
C	-3.39317600	-0.42162600	0.43816300
H	-3.64620800	-0.49424800	1.50405300
H	-3.50040400	-1.42963200	0.02418800
C	-4.39331200	0.52872400	-0.24046500
H	-5.42229800	0.18879000	-0.09335800
H	-4.31537300	1.54095400	0.16790800
H	-4.20516500	0.58936300	-1.31575300

R13

C	-3.08916100	0.40893300	0.91446500
C	-1.66632200	0.95162900	0.72015100
C	-0.65098500	-0.17729300	0.45762700
C	-1.10052800	-1.05135800	-0.72988300
C	-2.52830600	-1.58488200	-0.53756700
C	-3.52993200	-0.45267000	-0.27532300
H	-1.65639100	1.64839400	-0.12524900
H	-1.35946900	1.52900300	1.59885000
H	-3.12726700	-0.19444800	1.83099200
H	-3.78578100	1.24028600	1.06122200
H	-1.05401900	-0.45325100	-1.64699300
H	-0.41259400	-1.89091500	-0.86674000
H	-2.82779600	-2.16265700	-1.41779200
H	-2.54257800	-2.28197600	0.31067400
H	-3.60430400	0.17815800	-1.17023700
H	-4.52902600	-0.86380200	-0.09857400
H	-0.62398000	-0.81403500	1.35462900
C	0.77882900	0.37082200	0.31331500

H	0.98867100	1.06712600	1.12903600
C	1.88385800	-0.67568000	0.19712000
H	1.78502500	-1.19681800	-0.76064100
H	1.72321500	-1.42738400	0.97902600
C	3.29583400	-0.08981800	0.32004100
H	3.42518700	0.69274100	-0.43390800
H	3.39837600	0.40201700	1.29460900
O	0.85978500	1.19361800	-0.91137200
O	0.82215300	2.48858800	-0.66765900
C	4.39287000	-1.14532400	0.16001100
H	5.38659900	-0.70046700	0.25577900
H	4.30536800	-1.92915900	0.91902900
H	4.33832300	-1.62661800	-0.82121100

TS13

C	1.74207600	-0.54691300	-0.18914000
H	2.15874800	0.64822000	-0.59125100
C	0.61897700	-0.06064800	0.47471000
H	0.68660300	0.13107200	1.54300600
O	1.18977600	2.14414700	0.15793700
O	2.19425000	1.93968000	-0.59920300
C	-0.76027300	-0.03420300	-0.11797400
C	-1.42196400	-1.42748600	0.07101900
C	-1.66129300	1.06460300	0.47691100
H	-0.66489600	0.13693200	-1.19903700
C	-2.84378200	-1.46693500	-0.50788100
H	-1.45831100	-1.66063200	1.14318900
H	-0.80022600	-2.19705900	-0.39607500
C	-3.08331300	1.01876200	-0.09868300
H	-1.70652900	0.92982600	1.56624100
H	-1.21122600	2.04282500	0.30030900
C	-3.72903000	-0.36113000	0.08089500
H	-3.28807900	-2.45058600	-0.32421400
H	-2.79124800	-1.34706300	-1.59758100
H	-3.69841100	1.79078500	0.37429300
H	-3.04753500	1.26472800	-1.16791600
H	-4.71974100	-0.38217200	-0.38423900
H	-3.88229000	-0.55268900	1.15091200
H	1.55185700	-1.02269800	-1.15515800
C	2.94138400	-1.10570600	0.57698100
H	3.07480700	-0.52721200	1.49766800
H	2.72234400	-2.13363300	0.88940300
C	4.24221500	-1.09208500	-0.23252400
H	5.06572600	-1.52862400	0.33850800
H	4.13793200	-1.66925600	-1.15666800

H	4.52829400	-0.07293700	-0.50714400

P13

C	2.29218500	1.45588200	-0.18816500
C	0.80994300	1.12680700	0.04404500
C	0.44323600	-0.26589600	-0.49198900
C	1.37509400	-1.33838800	0.12054100
C	2.85794000	-1.01262200	-0.10588300
C	3.21343000	0.38658300	0.41473100
H	0.59732100	1.16521900	1.12021100
H	0.17927200	1.88734900	-0.42755600
H	2.48153800	1.52864200	-1.26705100
H	2.52619500	2.43878000	0.23367600
H	1.17169900	-1.40318000	1.19708300
H	1.13546000	-2.32080900	-0.30069200
H	3.48525700	-1.76880100	0.37742600
H	3.07896900	-1.06515300	-1.17997300
H	3.11394300	0.40015100	1.50794800
H	4.26039600	0.61936600	0.19447000
H	0.64114000	-0.26213400	-1.57625700
C	-1.00317400	-0.65682000	-0.31472200
H	-1.26082700	-1.62939600	-0.73735700
C	-1.96563500	0.03209100	0.29761400
H	-1.74011000	1.00633700	0.72781300
C	-3.39317600	-0.42162600	0.43816300
H	-3.64620800	-0.49424800	1.50405300
H	-3.50040400	-1.42963200	0.02418800
C	-4.39331200	0.52872400	-0.24046500
H	-5.42229800	0.18879000	-0.09335800
H	-4.31537300	1.54095400	0.16790800
H	-4.20516500	0.58936300	-1.31575300

R14

C	0.39835900	-0.03582800	0.22763600
C	-0.23373100	-1.35859200	-0.21932900
C	-1.64089600	-1.19384400	-0.81398400
C	-2.56861300	-0.41175200	0.12318400
C	-1.95950800	0.94947400	0.48040600
C	-0.55864400	0.79936500	1.08849200
H	-1.56620400	-0.66819800	-1.77166700
H	0.42391600	-1.84768400	-0.94195100
H	-0.27787400	-2.01056900	0.66126100
H	-3.54976700	-0.27800100	-0.34268500
H	-2.60330800	1.48567100	1.18429500
H	-0.10227700	1.77766100	1.25662000

H	-0.63100400	0.30567500	2.06471000
H	-2.05793500	-2.18220500	-1.03065900
H	-2.73460200	-0.99018000	1.04189800
H	-1.89821300	1.56889700	-0.42075300
O	0.61491800	0.68890700	-1.07203400
O	1.11148600	1.89625700	-0.92075600
C	1.76485900	-0.19375000	0.90895000
H	1.59347100	-0.67586400	1.87732000
H	2.13392800	0.81350200	1.12102500
C	2.82304700	-0.96958700	0.11817300
H	2.98222900	-0.52617400	-0.86714400
H	2.54989600	-2.01819600	-0.02150300
H	3.77727200	-0.94855200	0.65038800

TS14

C	1.98501900	0.66098300	0.86943600
C	0.66345700	0.13736400	1.45010200
C	-0.31416400	-0.38801600	0.42701800
C	0.26595800	-1.18762000	-0.70975800
C	1.60418000	-0.65569800	-1.24904200
C	2.59288600	-0.33744700	-0.12189400
H	0.88568500	-0.70492600	2.12596000
H	0.17649400	0.89796700	2.06667000
H	1.80140900	1.61174600	0.36184900
H	2.68376400	0.86224200	1.68736100
H	0.41796200	-2.20905300	-0.32156800
H	-0.45971700	-1.28317900	-1.51973000
H	2.03101900	-1.39312100	-1.93595200
H	1.41493200	0.25067100	-1.83057800
H	2.86298100	-1.26266900	0.40530900
H	3.52081900	0.06419600	-0.54040000
C	-1.68343900	-0.37263300	0.71667300
H	-1.91845700	-0.12177900	1.75359200
H	-1.92322400	0.80277300	0.15433200
O	-0.45552800	1.63323000	-0.79043500
O	-1.69295000	1.85503100	-0.55322400
C	-2.68660200	-1.31491700	0.06093600
H	-3.70642500	-1.01497600	0.31249500
H	-2.60892400	-1.30935900	-1.02853800
H	-2.55425500	-2.34871800	0.39763500

P14

C	1.14849100	-1.42231300	0.26711600
C	-0.09863300	-1.08676400	-0.57769700
C	-0.60409000	0.30775700	-0.28166800
C	0.46270700	1.37266800	-0.42439100

C	1.70886600	1.04519400	0.42177400
C	2.24287400	-0.35868800	0.10779100
H	0.17824600	-1.14195300	-1.64050800
H	-0.87127800	-1.84057000	-0.41809300
H	0.85548700	-1.48646700	1.32199300
H	1.53233100	-2.40828300	-0.01478200
H	0.76805400	1.43543900	-1.47920200
H	0.06107500	2.35263900	-0.15188600
H	2.48659000	1.79627600	0.24843600
H	1.44249000	1.10370300	1.48409100
H	2.61840700	-0.37944300	-0.92386000
H	3.09528300	-0.59368200	0.75354000
C	-1.84803300	0.61828000	0.10034700
H	-2.05816600	1.67047300	0.29031800
C	-3.02277100	-0.29702900	0.29781300
H	-3.84859600	-0.01715100	-0.36665600
H	-2.78796400	-1.34517500	0.10914400
H	-3.40842600	-0.22044000	1.32095500

R15

C	0.00575300	0.03836300	0.14502500
C	-0.51602300	-1.31220400	-0.35908400
C	-1.98166700	-1.26672700	-0.81876100
C	-2.90048900	-0.67167400	0.25471100
C	-2.41238700	0.71855500	0.67967700
C	-0.95359200	0.68404300	1.15440100
H	-2.05125900	-0.66347200	-1.72978400
H	0.12066100	-1.66511900	-1.17393400
H	-0.40615100	-2.02989300	0.46264800
H	-3.92780500	-0.61513800	-0.11820900
H	-3.04296000	1.11910500	1.47928300
H	-0.59305400	1.69176800	1.37357000
H	-0.88259800	0.10679300	2.08361800
H	-2.30679500	-2.27701600	-1.08579400
H	-2.92179400	-1.33575700	1.12922600
H	-2.50216400	1.41016900	-0.16469000
O	0.01231000	0.88110600	-1.10028100
O	0.38020300	2.12458000	-0.88898100
C	1.43895000	-0.00490800	0.69162900
H	1.41659200	-0.57173200	1.62966200
H	1.71252600	1.02199400	0.95381500
C	2.50946500	-0.58906500	-0.23895400
H	2.47336700	-0.07061300	-1.20174900
H	2.29819600	-1.64331500	-0.44426400

C	3.91585000	-0.46646400	0.35622000
H	4.18028700	0.58046700	0.53241100
H	3.99001100	-0.99245100	1.31339500
H	4.66766700	-0.89079800	-0.31428800

TS15

C	2.40628000	0.42262700	0.97739900
C	1.01048400	0.00504200	1.46363400
C	0.03218200	-0.33422400	0.36573600
C	0.57907700	-1.11678400	-0.79941900
C	1.98897700	-0.69656300	-1.24633700
C	2.95273600	-0.56260000	-0.06206500
H	1.11044000	-0.90509600	2.07786200
H	0.57983300	0.76559500	2.12103200
H	2.34814500	1.42211000	0.53790900
H	3.08224800	0.48775000	1.83570600
H	0.60513500	-2.17161900	-0.47666700
H	-0.11298700	-1.08224500	-1.64293600
H	2.36809400	-1.42766200	-1.96719600
H	1.92182400	0.26185100	-1.76822200
H	3.10177800	-1.54534100	0.40569100
H	3.93520000	-0.23333200	-0.41399600
C	-1.33954700	-0.18706200	0.59788300
H	-1.59893000	0.02307900	1.63933500
H	-1.42507800	1.04175800	0.10268500
O	0.16616200	1.76518600	-0.72133100
O	-1.05270900	2.10129300	-0.52502300
C	-2.41293300	-0.97117700	-0.15634900
H	-2.25032500	-0.89113500	-1.23555100
H	-2.33113900	-2.03850900	0.08534300
C	-3.82796800	-0.48553100	0.17422800
H	-3.95899900	0.56550900	-0.09857400
H	-4.58053400	-1.06724700	-0.36396900
H	-4.03805800	-0.57967600	1.24411800

P15

C	-1.67766000	-1.41764600	-0.23126000
C	-0.37187200	-1.09174500	0.52393700
C	0.12468300	0.29700600	0.18726300
C	-0.91946200	1.37145400	0.40637300
C	-2.22515200	1.05530200	-0.34970100
C	-2.74870800	-0.34375900	0.00161100
H	-0.57597800	-1.13889300	1.60340900
H	0.38096500	-1.85360600	0.31561600
H	-1.45939000	-1.48661800	-1.30377600
H	-2.04982600	-2.39946700	0.07933100

H	-1.14843500	1.43555800	1.48020000
H	-0.52971300	2.34804300	0.10610300
H	-2.98188600	1.81338000	-0.12238100
H	-2.03364100	1.11174600	-1.42813500
H	-3.05269900	-0.36071300	1.05661800
H	-3.64555300	-0.57123500	-0.58381400
C	1.33805000	0.59492100	-0.29237600
H	1.54451700	1.64625600	-0.49697100
C	2.49473600	-0.32585400	-0.57146300
H	2.21218900	-1.37100400	-0.42527900
H	2.77604900	-0.23034600	-1.62841200
C	3.72608800	-0.00383200	0.29308600
H	4.56872800	-0.65231400	0.03669800
H	4.04710400	1.03250300	0.15100700
H	3.50334300	-0.13837600	1.35496100

R16

C	-0.44728900	0.10740000	0.17543800
C	-0.81848800	-1.31633400	-0.25605500
C	-2.25735000	-1.43975500	-0.78137900
C	-3.27812000	-0.86598000	0.20804400
C	-2.94153400	0.58994300	0.55278900
C	-1.51124000	0.72667400	1.09190100
H	-2.33677500	-0.90586300	-1.73393100
H	-0.11198000	-1.66014900	-1.01534900
H	-0.68495900	-1.96553100	0.61750800
H	-4.28745800	-0.93343000	-0.20944900
H	-3.64547000	0.98249700	1.29297300
H	-1.25623100	1.77699100	1.25129000
H	-1.43399800	0.22542700	2.06372000
H	-2.47480700	-2.49149500	-0.99180000
H	-3.27811400	-1.46860400	1.12612800
H	-3.05109700	1.21077200	-0.34270000
O	-0.45301100	0.86302200	-1.12533600
O	-0.20878400	2.14668500	-0.98617200
C	0.95429800	0.23441300	0.78700100
H	0.93430100	-0.26844400	1.76073800
H	1.12020800	1.29761600	0.98728800
C	2.11687000	-0.30920600	-0.05262200
H	2.08200000	0.13526400	-1.05294900
H	2.01347200	-1.39146300	-0.18878500
C	3.48284300	-0.01886800	0.58168800
H	3.59735300	1.06400600	0.70905200
H	3.51392400	-0.44914800	1.59032200

C	4.65417200	-0.56247400	-0.24104900
H	4.66712400	-0.12613000	-1.24449500
H	5.61331900	-0.33565000	0.23215800
H	4.58857700	-1.64919600	-0.35419200

TS16

C	2.92440600	0.30462600	0.85974500
C	1.51759100	0.10375800	1.44239700
C	0.45172300	-0.22597100	0.42646500
C	0.84638300	-1.17531400	-0.67458300
C	2.26930400	-0.96807800	-1.21915400
C	3.30165000	-0.83442600	-0.09479100
H	1.54606200	-0.74544800	2.14493500
H	1.20981800	0.97171900	2.03240900
H	2.95666500	1.25637900	0.32250100
H	3.64699100	0.37343900	1.67891500
H	0.77081800	-2.19004200	-0.24793000
H	0.11961400	-1.14106500	-1.48822100
H	2.52593000	-1.80476500	-1.87631400
H	2.28287600	-0.06322900	-1.83275300
H	3.36298200	-1.77787400	0.46471000
H	4.29567100	-0.65834100	-0.51723400
C	-0.88194000	0.09929700	0.69728200
H	-1.06265700	0.43703600	1.72157000
H	-0.85637200	1.27655400	0.08441200
O	0.76278500	1.72968000	-0.86990100
O	-0.39977900	2.22171200	-0.66141200
C	-2.07049300	-0.62724500	0.06981800
H	-1.95082500	-0.68151200	-1.01746600
H	-2.10226000	-1.66643300	0.42443300
C	-3.41140100	0.04826100	0.38515700
H	-3.38832600	1.08129500	0.01993700
H	-3.53615300	0.11153900	1.47286300
C	-4.60884400	-0.68520800	-0.22385100
H	-4.67584600	-1.71233100	0.14859200
H	-5.54889700	-0.18273100	0.01860100
H	-4.53041300	-0.73352300	-1.31437200

P16

C	2.07800800	1.49062500	-0.23068500
C	0.80571900	1.05386600	0.52577600
C	0.42836800	-0.37152100	0.18789500
C	1.56036400	-1.35379500	0.40332800
C	2.83321300	-0.92672100	-0.35431100
C	3.23663500	0.51109200	-0.00119500
H	1.00653500	1.11680200	1.60505700

H	-0.00963300	1.74902400	0.31957700
H	1.85297300	1.54249200	-1.30276400
H	2.36604400	2.50000200	0.08099900
H	1.79596500	-1.39984800	1.47665100
H	1.25430100	-2.35947800	0.10211300
H	3.65195700	-1.61822000	-0.12959500
H	2.64525900	-0.99734600	-1.43252800
H	3.53972100	0.55203500	1.05342100
H	4.11007200	0.81485800	-0.58741900
C	-0.75646100	-0.77072200	-0.28976700
H	-0.87254400	-1.83545900	-0.49545300
C	-1.98723300	0.04920400	-0.56353000
H	-1.78913200	1.11760600	-0.44076900
H	-2.28019000	-0.08820200	-1.61372000
C	-3.18097700	-0.34758300	0.32626200
H	-3.36984300	-1.42188900	0.21574700
H	-2.90735100	-0.19356300	1.37583100
C	-4.45669200	0.43377400	0.00078200
H	-5.28529100	0.13166500	0.64711900
H	-4.30728300	1.50986900	0.13446700
H	-4.76722100	0.27033800	-1.03606900

R17

C	-0.44001600	1.27168800	0.06878600
C	-1.97452800	1.26738700	0.02658800
C	-2.51793400	-0.00014700	-0.64705100
C	-1.97433400	-1.26754500	0.02668900
C	-0.43981900	-1.27153700	0.06899400
C	0.11576500	0.00017500	0.73880300
H	-3.61232400	-0.00021500	-0.62541300
H	-2.36574800	1.32798300	1.05064500
H	-2.33438700	2.15929800	-0.49575800
H	-0.04606500	1.33897900	-0.95136100
H	-0.07954900	2.15840100	0.60219700
H	-2.36560100	-1.32815500	1.05072500
H	-2.33396800	-2.15956300	-0.49562500
H	-0.07923000	-2.15809900	0.60257500
H	-0.04579300	-1.33893600	-0.95112700
H	-0.21772500	0.00029400	1.78707000
H	-2.22613400	-0.00016900	-1.70495500
C	1.63636800	0.00014700	0.80593200
H	2.03053500	-0.89176400	1.29906800
O	2.16765500	-0.00006900	-0.55749900
O	3.48386400	-0.00006200	-0.56742700

H	2.03082700	0.89198500	1.29892200

TS17

C	-1.56943600	-1.27010300	-0.43543400
C	-0.50470700	-1.28505000	0.67283700
C	0.33371600	0.00010100	0.67997500
C	-0.50479100	1.28521400	0.67258800
C	-1.56962000	1.26997000	-0.43561200
C	-2.43020100	-0.00011600	-0.37349600
H	-1.00497500	-1.38318500	1.64565100
H	0.14762600	-2.15719100	0.56503400
H	-1.07546700	-1.32444300	-1.41343500
H	-2.20110500	-2.16024200	-0.35496600
H	-1.00500200	1.38353000	1.64541600
H	0.14748800	2.15736900	0.56455100
H	-2.20137400	2.16004900	-0.35516200
H	-1.07574600	1.32429400	-1.41366000
H	-3.00803900	-0.00009300	0.56020400
H	-3.15885300	-0.00021000	-1.19013600
H	0.88003200	-0.00000700	-0.52843500
C	1.57800700	0.00013700	1.30091500
H	2.05385700	0.91918200	1.62080800
H	2.05380600	-0.91889800	1.62092300
O	2.81475100	-0.00005800	-0.54470200
O	1.86649200	-0.00007700	-1.38747600

P17

C	-1.02041500	-1.26825400	-0.30049600
C	0.34520200	-1.26902300	0.41644000
C	1.11528300	0.00002900	0.13174000
C	0.34518800	1.26904400	0.41642000
C	-1.02043400	1.26824700	-0.30049400
C	-1.82234900	-0.00002300	0.02202700
H	0.16915400	-1.34293700	1.49924500
H	0.93227200	-2.14535600	0.12873100
H	-0.85326500	-1.32627500	-1.38278200
H	-1.58888500	-2.16080400	-0.01992200
H	0.16913000	1.34295000	1.49922400
H	0.93224000	2.14540300	0.12873400
H	-1.58892900	2.16077500	-0.01990200
H	-0.85330900	1.32627500	-1.38278200
H	-2.08568500	-0.00002600	1.08795900
H	-2.76687200	-0.00001800	-0.53152500
C	2.35263900	-0.00000600	-0.36305800
H	2.88176800	0.92444000	-0.57124500
H	2.88169200	-0.92450600	-0.57120700

R18

C	-0.61754600	1.09998400	-0.53284900
C	-2.14765800	1.21275300	-0.45373000
C	-2.82400100	-0.16314500	-0.49957000
C	-2.27528100	-1.08600500	0.59632800
C	-0.74692100	-1.20360100	0.52125100
C	-0.05786700	0.17457100	0.56660600
H	-3.90866500	-0.05604000	-0.39759700
H	-2.42526200	1.72038600	0.47950200
H	-2.51343900	1.84383600	-1.26984500
H	-0.33093700	0.70322000	-1.51290600
H	-0.17349000	2.09668300	-0.45560100
H	-2.56048600	-0.68826100	1.57924000
H	-2.72897900	-2.07920800	0.52040300
H	-0.37748500	-1.82734100	1.34304800
H	-0.46431600	-1.71155400	-0.40782200
H	-0.28991600	0.63118900	1.53986900
H	-2.64569000	-0.62134600	-1.48078500
C	1.46888700	0.03375500	0.54059500
C	2.26953900	1.30696300	0.76790800
H	1.77991900	-0.73396900	1.25437700
H	3.33565800	1.08228200	0.71238600
H	2.03496600	2.06818500	0.02179800
H	2.05198100	1.71149000	1.75991100
O	1.81920400	-0.51786900	-0.78595300
O	3.03094900	-1.03103200	-0.80469800

TS18

C	-2.03479500	-0.97604100	0.68144100
C	-0.78786300	-0.41255600	1.37820600
C	0.17672200	0.26419300	0.39074000
C	-0.51083400	1.28907400	-0.52058600
C	-1.76806200	0.71139600	-1.19125500
C	-2.73204000	0.09424000	-0.16919700
H	-1.10422100	0.32787600	2.12576900
H	-0.26519900	-1.20530100	1.92269100
H	-1.74282600	-1.81589100	0.03914100
H	-2.72674900	-1.37876200	1.42785800
H	-0.79695600	2.16170100	0.08331400
H	0.17770500	1.65492300	-1.28626400
H	-2.27316400	1.49653900	-1.76272500
H	-1.46889000	-0.05763600	-1.91394000
H	-3.11872600	0.88442100	0.48819500
H	-3.59872400	-0.33535800	-0.68142700

H	0.47866300	-0.73155100	-0.43556400
C	1.50651200	0.42740900	0.78817700
H	1.83289100	-0.11974800	1.66762200
O	2.32944700	-1.27904300	-0.45916500
O	1.23688600	-1.60984600	-1.02467300
C	2.46935300	1.44573100	0.26886100
H	3.49931900	1.10127800	0.37464000
H	2.29307900	1.69023100	-0.77963100
H	2.36916500	2.37772500	0.84270600

P18

C	1.14849100	-1.42231300	0.26711600
C	-0.09863300	-1.08676400	-0.57769700
C	-0.60409000	0.30775700	-0.28166800
C	0.46270700	1.37266800	-0.42439100
C	1.70886600	1.04519400	0.42177400
C	2.24287400	-0.35868800	0.10779100
H	0.17824600	-1.14195300	-1.64050800
H	-0.87127800	-1.84057000	-0.41809300
H	0.85548700	-1.48646700	1.32199300
H	1.53233100	-2.40828300	-0.01478200
H	0.76805400	1.43543900	-1.47920200
H	0.06107500	2.35263900	-0.15188600
H	2.48659000	1.79627600	0.24843600
H	1.44249000	1.10370300	1.48409100
H	2.61840700	-0.37944300	-0.92386000
H	3.09528300	-0.59368200	0.75354000
C	-1.84803300	0.61828000	0.10034700
H	-2.05816600	1.67047300	0.29031800
C	-3.02277100	-0.29702900	0.29781300
H	-3.84859600	-0.01715100	-0.36665600
H	-2.78796400	-1.34517500	0.10914400
H	-3.40842600	-0.22044000	1.32095500

R19

C	-2.60263000	0.77234500	0.85352200
C	-1.10604100	1.06220400	0.67043200
C	-0.28944400	-0.23021200	0.47784400
C	-0.85850000	-1.06688800	-0.68518300
C	-2.35780200	-1.34823200	-0.50402800
C	-3.16179800	-0.05646600	-0.31005000
H	-0.96622300	1.71051700	-0.20166200
H	-0.72122100	1.61771000	1.53231700
H	-2.75459900	0.22418000	1.79260400
H	-3.15323900	1.71325300	0.95051600

H	-0.69974300	-0.52292900	-1.62311200
H	-0.31884800	-2.01410700	-0.77543600
H	-2.73540200	-1.90555400	-1.36718900
H	-2.50062400	-1.99673900	0.37045100
H	-3.11765500	0.53855500	-1.23114900
H	-4.21767100	-0.28921100	-0.13859400
H	-0.38596300	-0.82335500	1.39963600
C	1.21443000	0.06418400	0.34757500
H	1.52489800	0.74939100	1.14039900
C	2.12884400	-1.15789900	0.30093200
H	1.94113800	-1.70927300	-0.62483900
H	1.84147600	-1.82171800	1.12360600
C	3.61485900	-0.80294400	0.40305800
H	3.91257000	-0.13018300	-0.40417700
H	3.84001300	-0.30742500	1.35212300
O	1.45411800	0.80796500	-0.90602300
O	1.64389800	2.09832500	-0.71385200
H	4.23545000	-1.69997700	0.33888600

TS19

C	2.48155600	0.70819500	0.84117300
C	1.13446100	0.24920000	1.41791100
C	0.15729000	-0.20952700	0.32319100
C	0.76722800	-1.22505700	-0.65156700
C	2.12564400	-0.75529100	-1.19789900
C	3.09240900	-0.36301200	-0.07269700
H	1.31149900	-0.58784000	2.10725700
H	0.67909300	1.05108400	2.00738400
H	2.33535600	1.63221000	0.26851000
H	3.17095400	0.95060500	1.65603800
H	0.90379500	-2.18033300	-0.12504900
H	0.08511700	-1.42411900	-1.48189100
H	2.56498500	-1.54307500	-1.81793400
H	1.96904000	0.10869400	-1.85536700
H	3.33508400	-1.25247300	0.52389700
H	4.03630500	-0.00518900	-0.49589700
H	0.02974600	0.88309200	-0.41332600
C	-1.20379600	-0.24591400	0.63692600
H	-1.52077400	0.25590800	1.54751700
O	-1.72845500	1.68673400	-0.47481400
O	-0.56807400	1.90882300	-0.95254800
C	-2.24201300	-1.09723100	-0.02975100
H	-2.07446000	-1.11316700	-1.11105600
H	-2.08392100	-2.13504300	0.30414000
C	-3.68274900	-0.67659700	0.27398100

H	-4.39471100	-1.36018000	-0.19494800
H	-3.87601800	-0.68301000	1.35091400
H	-3.87903900	0.32978800	-0.09889300

P19

C	-1.67766000	-1.41764600	-0.23126000
C	-0.37187200	-1.09174500	0.52393700
C	0.12468300	0.29700600	0.18726300
C	-0.91946200	1.37145400	0.40637300
C	-2.22515200	1.05530200	-0.34970100
C	-2.74870800	-0.34375900	0.00161100
H	-0.57597800	-1.13889300	1.60340900
H	0.38096500	-1.85360600	0.31561600
H	-1.45939000	-1.48661800	-1.30377600
H	-2.04982600	-2.39946700	0.07933100
H	-1.14843500	1.43555800	1.48020000
H	-0.52971300	2.34804300	0.10610300
H	-2.98188600	1.81338000	-0.12238100
H	-2.03364100	1.11174600	-1.42813500
H	-3.05269900	-0.36071300	1.05661800
H	-3.64555300	-0.57123500	-0.58381400
C	1.33805000	0.59492100	-0.29237600
H	1.54451700	1.64625600	-0.49697100
C	2.49473600	-0.32585400	-0.57146300
H	2.21218900	-1.37100400	-0.42527900
H	2.77604900	-0.23034600	-1.62841200
C	3.72608800	-0.00383200	0.29308600
H	4.56872800	-0.65231400	0.03669800
H	4.04710400	1.03250300	0.15100700
H	3.50334300	-0.13837600	1.35496100

R20

C	-3.08916100	0.40893300	0.91446500
C	-1.66632200	0.95162900	0.72015100
C	-0.65098500	-0.17729300	0.45762700
C	-1.10052800	-1.05135800	-0.72988300
C	-2.52830600	-1.58488200	-0.53756700
C	-3.52993200	-0.45267000	-0.27532300
H	-1.65639100	1.64839400	-0.12524900
H	-1.35946900	1.52900300	1.59885000
H	-3.12726700	-0.19444800	1.83099200
H	-3.78578100	1.24028600	1.06122200
H	-1.05401900	-0.45325100	-1.64699300
H	-0.41259400	-1.89091500	-0.86674000
H	-2.82779600	-2.16265700	-1.41779200

H	-2.54257800	-2.28197600	0.31067400
H	-3.60430400	0.17815800	-1.17023700
H	-4.52902600	-0.86380200	-0.09857400
H	-0.62398000	-0.81403500	1.35462900
C	0.77882900	0.37082200	0.31331500
H	0.98867100	1.06712600	1.12903600
C	1.88385800	-0.67568000	0.19712000
H	1.78502500	-1.19681800	-0.76064100
H	1.72321500	-1.42738400	0.97902600
C	3.29583400	-0.08981800	0.32004100
H	3.42518700	0.69274100	-0.43390800
H	3.39837600	0.40201700	1.29460900
O	0.85978500	1.19361800	-0.91137200
O	0.82215300	2.48858800	-0.66765900
C	4.39287000	-1.14532400	0.16001100
H	5.38659900	-0.70046700	0.25577900
H	4.30536800	-1.92915900	0.91902900
H	4.33832300	-1.62661800	-0.82121100

TS20

C	3.00457800	0.50855000	0.75061500
C	1.62627400	0.27976500	1.38735600
C	0.56281900	-0.11486400	0.34931700
C	0.99983000	-1.26783900	-0.56332600
C	2.39311800	-1.02980400	-1.16864900
C	3.43727900	-0.69716300	-0.09411800
H	1.70917400	-0.52339500	2.13252300
H	1.30268400	1.17566800	1.92648400
H	2.96732800	1.40141200	0.11462500
H	3.74493500	0.71081700	1.53103000
H	1.01889000	-2.19525200	0.02648900
H	0.27275300	-1.42559500	-1.36401000
H	2.70189400	-1.91243200	-1.73770800
H	2.33732700	-0.20088200	-1.88488900
H	3.57423300	-1.56803800	0.56068600
H	4.40817300	-0.50322100	-0.56088300
H	0.56003400	0.93253100	-0.45878500
C	-0.78045400	0.05510800	0.69473500
H	-0.99840200	0.65281300	1.57594100
O	-1.07127300	1.96965000	-0.53097000
O	0.09077600	1.99515800	-1.05354900
C	-1.94353500	-0.68479300	0.10694600
H	-1.82602500	-0.76746700	-0.97899700
H	-1.90859500	-1.72087300	0.48234300
C	-3.31088200	-0.07428700	0.43832400

H	-3.41643100	-0.00289700	1.52743500
H	-3.34276000	0.94901900	0.05515300
C	-4.47653000	-0.88239600	-0.13648500
H	-4.48307100	-1.90601900	0.25174200
H	-5.43689300	-0.42582200	0.11625600
H	-4.41625500	-0.94248000	-1.22757800

P20

C	2.07800800	1.49062500	-0.23068500
C	0.80571900	1.05386600	0.52577600
C	0.42836800	-0.37152100	0.18789500
C	1.56036400	-1.35379500	0.40332800
C	2.83321300	-0.92672100	-0.35431100
C	3.23663500	0.51109200	-0.00119500
H	1.00653500	1.11680200	1.60505700
H	-0.00963300	1.74902400	0.31957700
H	1.85297300	1.54249200	-1.30276400
H	2.36604400	2.50000200	0.08099900
H	1.79596500	-1.39984800	1.47665100
H	1.25430100	-2.35947800	0.10211300
H	3.65195700	-1.61822000	-0.12959500
H	2.64525900	-0.99734600	-1.43252800
H	3.53972100	0.55203500	1.05342100
H	4.11007200	0.81485800	-0.58741900
C	-0.75646100	-0.77072200	-0.28976700
H	-0.87254400	-1.83545900	-0.49545300
C	-1.98723300	0.04920400	-0.56353000
H	-1.78913200	1.11760600	-0.44076900
H	-2.28019000	-0.08820200	-1.61372000
C	-3.18097700	-0.34758300	0.32626200
H	-3.36984300	-1.42188900	0.21574700
H	-2.90735100	-0.19356300	1.37583100
C	-4.45669200	0.43377400	0.00078200
H	-5.28529100	0.13166500	0.64711900
H	-4.30728300	1.50986900	0.13446700
H	-4.76722100	0.27033800	-1.03606900

R21

C	-0.63918300	0.36132000	0.26074100
C	0.37096500	1.43660200	-0.15320200
C	1.64143700	0.86320900	-0.79726300
C	2.28647200	-0.21133700	0.08724400
C	1.28413600	-1.32516100	0.41639300
C	0.01102200	-0.76786000	1.06844500
H	1.38691300	0.42864200	-1.76942700

H	-0.12164500	2.14035700	-0.83127300
H	0.63488600	1.99949000	0.74974400
H	3.16716300	-0.62991400	-0.40938100
H	1.73759800	-2.06489900	1.08306600
H	-0.72993600	-1.55740800	1.21430300
H	0.24931000	-0.36217100	2.05877600
H	2.34840100	1.67564700	-0.99126500
H	2.64142900	0.24740000	1.01986600
H	1.01535500	-1.85601700	-0.50297400
O	-1.09281200	-0.19857800	-1.05865700
O	-1.94970100	-1.18707200	-0.93850700
C	-1.86722800	0.94316600	0.94920000
H	-1.58947900	1.36811400	1.91668300
H	-2.61300100	0.16339800	1.11065700
H	-2.31261400	1.73293200	0.33918000

TS21

C	-1.40955200	-0.98227200	0.95426900
C	0.01904500	-0.43349400	1.11326000
C	0.46782900	0.67735900	0.38447200
C	-0.35545200	1.27792900	-0.72336000
C	-1.52443200	0.39254300	-1.16402600
C	-2.29826700	-0.10402700	0.06150800
H	0.44621600	-0.53310200	2.11238100
H	0.78003400	-1.29797000	0.48560200
H	-1.37271500	-1.99516000	0.53650200
H	-1.86294300	-1.08353600	1.94473300
H	-0.74574900	2.23785000	-0.34879100
H	0.29522100	1.52781300	-1.56669100
H	-2.17943600	0.95394300	-1.83633500
H	-1.14120900	-0.46156800	-1.73300500
H	-2.64968800	0.76434700	0.63291900
H	-3.18882700	-0.66243500	-0.24087700
C	1.63811200	1.48211800	0.86789200
H	2.29091800	0.89363700	1.51527200
H	2.22861600	1.86873300	0.03449000
H	1.27741400	2.34252800	1.44726700
O	1.74433300	-0.58530400	-1.03157000
O	1.58047500	-1.71670000	-0.46187400

P21

C	1.19481600	1.29806000	-0.10242100
C	-0.31142300	1.21919100	-0.08907800
C	-1.00986500	0.08373100	0.00536900
C	-0.32670900	-1.26609900	0.08243200
C	1.15631100	-1.21455900	-0.30841600

C	1.85102500	-0.01524100	0.34452300
H	1.53814800	1.56252400	-1.11309000
H	1.52409500	2.12200500	0.54154900
H	-0.43364000	-1.66073500	1.10337100
H	-0.85982700	-1.97726900	-0.56072100
H	1.65194100	-2.14942400	-0.02902900
H	1.23920000	-1.12697400	-1.39851300
H	1.77260500	-0.10568200	1.43473000
H	2.91846700	-0.00719000	0.10377800
C	-2.51499400	0.06648600	0.04931900
H	-2.93216600	1.07542400	0.02568900
H	-2.92859100	-0.49502000	-0.79694900
H	-2.87576600	-0.42949300	0.95864900
H	-0.84943100	2.16242000	-0.15983700

R22

C	0.39835900	-0.03582800	0.22763600
C	-0.23373100	-1.35859200	-0.21932900
C	-1.64089600	-1.19384400	-0.81398400
C	-2.56861300	-0.41175200	0.12318400
C	-1.95950800	0.94947400	0.48040600
C	-0.55864400	0.79936500	1.08849200
H	-1.56620400	-0.66819800	-1.77166700
H	0.42391600	-1.84768400	-0.94195100
H	-0.27787400	-2.01056900	0.66126100
H	-3.54976700	-0.27800100	-0.34268500
H	-2.60330800	1.48567100	1.18429500
H	-0.10227700	1.77766100	1.25662000
H	-0.63100400	0.30567500	2.06471000
H	-2.05793500	-2.18220500	-1.03065900
H	-2.73460200	-0.99018000	1.04189800
H	-1.89821300	1.56889700	-0.42075300
O	0.61491800	0.68890700	-1.07203400
O	1.11148600	1.89625700	-0.92075600
C	1.76485900	-0.19375000	0.90895000
H	1.59347100	-0.67586400	1.87732000
H	2.13392800	0.81350200	1.12102500
C	2.82304700	-0.96958700	0.11817300
H	2.98222900	-0.52617400	-0.86714400
H	2.54989600	-2.01819600	-0.02150300
H	3.77727200	-0.94855200	0.65038800

TS22

C	2.03165600	0.48661000	0.91492300
C	0.50560400	0.53750200	1.10196300

C	-0.35727300	-0.32574600	0.41031600
C	0.14585100	-1.20747300	-0.70050500
C	1.56475600	-0.86257000	-1.16508100
C	2.49179400	-0.68830200	0.04152500
H	0.17261000	0.80983700	2.10525800
H	0.12756100	1.61847200	0.47082500
H	2.38143300	1.42405700	0.46690500
H	2.50768400	0.42883900	1.89831400
H	0.12679000	-2.24239900	-0.32182700
H	-0.55373000	-1.18489500	-1.53958200
H	1.93346900	-1.64883300	-1.83004300
H	1.54174800	0.06305900	-1.75093500
H	2.48257300	-1.61276100	0.63271200
H	3.52523500	-0.53141200	-0.28066300
C	-1.72389000	-0.61246900	0.98692800
H	-2.03671600	0.25117000	1.58169800
H	-1.58970000	-1.43460400	1.70739900
O	-1.06764500	1.34593800	-1.02726800
O	-0.45667000	2.31963200	-0.46805700
C	-2.83400600	-0.99304000	0.00146200
H	-2.60244400	-1.91286000	-0.54093100
H	-3.76919600	-1.16069800	0.54167500
H	-2.99974600	-0.19860300	-0.72740100

P22

C	1.31925000	1.46440600	-0.10369100
C	-0.12638200	1.03016600	-0.09903400
C	-0.53540600	-0.23877400	-0.00370200
C	0.45611300	-1.38279100	0.08914800
C	1.88402300	-0.98261900	-0.30239900
C	2.27137900	0.34923700	0.34719600
H	1.59306200	1.80350100	-1.11329500
H	1.43798700	2.34399700	0.53997500
H	0.44626300	-1.77844600	1.11507400
H	0.11221400	-2.20987700	-0.54423600
H	2.58701000	-1.77207900	-0.01884200
H	1.94602000	-0.88198600	-1.39282000
H	2.21630600	0.24502000	1.43762700
H	3.30594300	0.61292900	0.10686400
C	-1.98888400	-0.65850600	0.02906800
H	-2.17011900	-1.32790100	-0.82349200
H	-2.13810500	-1.29021200	0.91618100
H	-0.86152700	1.82541500	-0.17999100
C	-3.03670500	0.45481800	0.02457400
H	-2.97075100	1.06611000	-0.87954800

H	-4.04319000	0.03019000	0.06310300
H	-2.92144400	1.11772200	0.88644700

R23

C	0.00575300	0.03836300	0.14502500
C	-0.51602300	-1.31220400	-0.35908400
C	-1.98166700	-1.26672700	-0.81876100
C	-2.90048900	-0.67167400	0.25471100
C	-2.41238700	0.71855500	0.67967700
C	-0.95359200	0.68404300	1.15440100
H	-2.05125900	-0.66347200	-1.72978400
H	0.12066100	-1.66511900	-1.17393400
H	-0.40615100	-2.02989300	0.46264800
H	-3.92780500	-0.61513800	-0.11820900
H	-3.04296000	1.11910500	1.47928300
H	-0.59305400	1.69176800	1.37357000
H	-0.88259800	0.10679300	2.08361800
H	-2.30679500	-2.27701600	-1.08579400
H	-2.92179400	-1.33575700	1.12922600
H	-2.50216400	1.41016900	-0.16469000
O	0.01231000	0.88110600	-1.10028100
O	0.38020300	2.12458000	-0.88898100
C	1.43895000	-0.00490800	0.69162900
H	1.41659200	-0.57173200	1.62966200
H	1.71252600	1.02199400	0.95381500
C	2.50946500	-0.58906500	-0.23895400
H	2.47336700	-0.07061300	-1.20174900
H	2.29819600	-1.64331500	-0.44426400
C	3.91585000	-0.46646400	0.35622000
H	4.18028700	0.58046700	0.53241100
H	3.99001100	-0.99245100	1.31339500
H	4.66766700	-0.89079800	-0.31428800

TS23

C	2.41466900	0.13510100	1.06306200
C	0.90550600	0.42369400	1.14091300
C	-0.02128300	-0.23544100	0.31902400
C	0.43003800	-1.11050800	-0.81954600
C	1.91969700	-0.97062100	-1.14930100
C	2.75565900	-1.03611800	0.13155900
H	0.53509300	0.67696400	2.13599100
H	0.75994200	1.59368400	0.57507500
H	2.94608300	1.03081600	0.72074700
H	2.78907600	-0.06855200	2.07069000
H	0.21737500	-2.15181200	-0.52737500

H	-0.18268700	-0.91829000	-1.70344600
H	2.21510900	-1.75830800	-1.84830900
H	2.09274900	-0.01515800	-1.65682500
H	2.54912700	-1.98527300	0.64208100
H	3.82469100	-1.02974600	-0.09991700
C	-1.46018300	-0.32948800	0.76796500
H	-1.68561400	0.53665600	1.39952800
H	-1.52301100	-1.20478300	1.43507900
O	-0.32373700	1.62523800	-1.02874400
O	0.38034200	2.44443700	-0.34497400
C	-2.53652400	-0.46395600	-0.31800400
H	-2.35829100	-1.36757000	-0.90970300
H	-2.45925700	0.38259900	-1.00462900
C	-3.94688100	-0.52350200	0.27619500
H	-4.17345600	0.38088700	0.84927200
H	-4.70188400	-0.61508700	-0.50881200
H	-4.06206700	-1.37939300	0.94911100

P23

C	1.88185800	1.45047100	-0.09866100
C	0.42804200	1.04442000	-0.10716000
C	-0.00668800	-0.21641300	-0.01560300
C	0.96228800	-1.37915400	0.08581600
C	2.40122500	-1.00667000	-0.29246500
C	2.80821800	0.31744000	0.36089100
H	2.17131300	1.78420700	-1.10570300
H	2.01153000	2.32773800	0.54610100
H	0.93545800	-1.77469900	1.11149700
H	0.60832800	-2.19937000	-0.55088400
H	3.08612500	-1.80962100	-0.00257200
H	2.47523300	-0.90726100	-1.38226200
H	2.74101700	0.21442700	1.45075800
H	3.84984000	0.56119800	0.13017400
C	-1.46774000	-0.60994000	0.00385500
H	-1.65629100	-1.27148600	-0.85450400
H	-1.63641300	-1.24483200	0.88647700
H	-0.29019000	1.85413100	-0.19484900
C	-2.50546600	0.51540500	-0.00072000
H	-2.37212300	1.13385200	-0.89496900
H	-2.33418500	1.17362800	0.85803800
C	-3.94265000	-0.01337900	0.03950900
H	-4.12083100	-0.60944600	0.94009000
H	-4.66789100	0.80473300	0.03473500
H	-4.15543900	-0.65028000	-0.82489400

R24

C	-0.44728900	0.10740000	0.17543800
C	-0.81848800	-1.31633400	-0.25605500
C	-2.25735000	-1.43975500	-0.78137900
C	-3.27812000	-0.86598000	0.20804400
C	-2.94153400	0.58994300	0.55278900
C	-1.51124000	0.72667400	1.09190100
H	-2.33677500	-0.90586300	-1.73393100
H	-0.11198000	-1.66014900	-1.01534900
H	-0.68495900	-1.96553100	0.61750800
H	-4.28745800	-0.93343000	-0.20944900
H	-3.64547000	0.98249700	1.29297300
H	-1.25623100	1.77699100	1.25129000
H	-1.43399800	0.22542700	2.06372000
H	-2.47480700	-2.49149500	-0.99180000
H	-3.27811400	-1.46860400	1.12612800
H	-3.05109700	1.21077200	-0.34270000
O	-0.45301100	0.86302200	-1.12533600
O	-0.20878400	2.14668500	-0.98617200
C	0.95429800	0.23441300	0.78700100
H	0.93430100	-0.26844400	1.76073800
H	1.12020800	1.29761600	0.98728800
C	2.11687000	-0.30920600	-0.05262200
H	2.08200000	0.13526400	-1.05294900
H	2.01347200	-1.39146300	-0.18878500
C	3.48284300	-0.01886800	0.58168800
H	3.59735300	1.06400600	0.70905200
H	3.51392400	-0.44914800	1.59032200
C	4.65417200	-0.56247400	-0.24104900
H	4.66712400	-0.12613000	-1.24449500
H	5.61331900	-0.33565000	0.23215800
H	4.58857700	-1.64919600	-0.35419200

TS24

C	2.92906900	0.08410600	0.94057400
C	1.44587500	0.46057800	1.10124900
C	0.43126600	-0.18891600	0.38126000
C	0.75725600	-1.14375900	-0.73575000
C	2.22398500	-1.09605700	-1.17466600
C	3.14511100	-1.14567200	0.04751200
H	1.15905400	0.78635700	2.10284100
H	1.32364300	1.60347300	0.48048400
H	3.48286700	0.93239300	0.52163300
H	3.35850400	-0.09420400	1.93093200
H	0.51729100	-2.15642600	-0.37265200

H	0.09123300	-0.96620600	-1.58341100
H	2.43087800	-1.92912700	-1.85252500
H	2.40581900	-0.17478500	-1.73893800
H	2.92877400	-2.05800300	0.61748400
H	4.19400200	-1.20609700	-0.25671100
C	-0.97585500	-0.18002200	0.92954900
H	-1.10683700	0.72425800	1.53357300
H	-1.03993000	-1.02065100	1.63945700
O	0.13550200	1.61265200	-1.04831500
O	0.92826200	2.42571900	-0.46118300
C	-2.13138500	-0.29660200	-0.07365300
H	-2.05499300	-1.23947500	-0.62681400
H	-2.05013400	0.50779800	-0.80999900
C	-3.50335700	-0.23225800	0.60812200
H	-3.58854400	0.71272100	1.15799700
H	-3.57583800	-1.02882500	1.35911400
C	-4.66855100	-0.35278500	-0.37795300
H	-4.63471800	-1.30500900	-0.91661100
H	-5.63256500	-0.29572200	0.13468200
H	-4.63909900	0.44888200	-1.12201600

P24

C	2.27280900	1.54209500	-0.10167800
C	0.86379900	1.00071600	-0.11252000
C	0.54992500	-0.29536400	-0.01854900
C	1.62390100	-1.36155500	0.08768300
C	3.02270900	-0.85472700	-0.28576100
C	3.29898400	0.50283400	0.36686800
H	2.53397700	1.89588900	-1.10961000
H	2.31710600	2.43103000	0.53858600
H	1.63084600	-1.75684400	1.11382400
H	1.35134100	-2.21225400	-0.54916500
H	3.77950500	-1.58870500	0.00818500
H	3.09116900	-0.75019500	-1.37542000
H	3.23460300	0.39586000	1.45650500
H	4.31444400	0.84311900	0.14185900
C	-0.86743000	-0.82563900	0.00076500
H	-0.98880200	-1.50741000	-0.85375900
H	-0.97553500	-1.46854400	0.88704500
H	0.07164200	1.73793900	-0.20389400
C	-2.00665200	0.19590300	-0.01253700
H	-1.93392300	0.81783200	-0.91264000
H	-1.90074500	0.87587200	0.84115600
C	-3.39355100	-0.45698500	0.03300400
H	-3.47034500	-1.08041900	0.93211100

H	-3.50077500	-1.13914800	-0.81914100
C	-4.53936600	0.55858600	0.01907400
H	-4.51137300	1.17441900	-0.88538000
H	-5.51348500	0.06301000	0.05274600
H	-4.48041900	1.23335900	0.87890200

R25

C	-1.22930900	1.03453300	-0.59600100
C	-2.16599700	-0.14169900	-0.28501400
C	-1.38658000	-1.45641100	-0.14774300
C	-0.26586900	-1.33947200	0.89440500
C	0.64729400	-0.14273000	0.63269000
C	-0.11097700	1.17826300	0.45298600
H	-2.05899700	-2.27417300	0.12790800
H	-2.70550200	0.06104200	0.64962800
H	-2.92515200	-0.23360000	-1.06781000
H	-0.77694300	0.89224500	-1.58409200
H	-1.79746700	1.96914100	-0.64421400
H	-0.69491300	-1.21415500	1.89582800
H	0.34856300	-2.24287900	0.92108100
H	1.41212200	-0.05648700	1.40844800
H	-0.58904000	1.36271300	1.42502700
H	-0.94990600	-1.72298700	-1.11625000
C	0.82619100	2.35423900	0.15315800
H	1.33533000	2.21751900	-0.80338700
H	1.59118300	2.46187800	0.92792600
O	1.39604900	-0.37126800	-0.62220200
O	2.31130900	-1.30760900	-0.48699600
H	0.26334300	3.29041700	0.10659800

TS25

C	-2.18233800	-0.10722500	-0.34873300
C	-1.15848300	0.95855300	-0.75817500
C	-0.21040000	-1.24831800	0.82940400
C	-1.50039400	-1.43599700	0.01218600
H	-0.56949900	0.60604200	-1.61362800
H	-1.66452600	1.87437600	-1.07872200
H	-2.74518200	0.26193200	0.51747800
H	-2.91122800	-0.26931300	-1.14813200
H	0.75064600	-1.67621100	0.06077300
H	-0.06563500	-1.94983100	1.65201200
H	-1.27646600	-1.98694000	-0.90856700
H	-2.19271300	-2.06688400	0.57702300
C	0.85085900	2.32645100	0.05569900
H	0.37407000	3.26699800	-0.23380400

H	1.50437100	2.53028700	0.90874100
H	1.47647500	1.98676300	-0.77052200
O	2.09479300	-0.34366100	-0.37570300
O	1.84485000	-1.55897000	-0.66239400
C	-0.21365800	1.27936000	0.40884800
H	-0.83325300	1.71424900	1.21379300
C	0.34187300	0.02252100	1.02005300
H	1.07104900	0.15751500	1.81263600

P25

C	-1.91554500	0.15073900	0.26135300
C	-1.01087200	1.34305300	0.07827300
C	0.28388700	1.24711300	-0.22558300
C	1.02669200	-0.06108700	-0.38594000
C	0.19875900	-1.23208500	0.17100300
C	-1.27005400	-1.14222800	-0.25772700
H	-2.16859700	0.04586100	1.32596300
H	-2.86888000	0.33102500	-0.24889700
H	1.17611300	-0.23100300	-1.46342200
H	0.63728400	-2.18222400	-0.15134600
H	0.25582200	-1.21481300	1.26718400
H	-1.32576200	-1.15749900	-1.35276500
H	-1.82839600	-2.01363500	0.09760400
H	-1.45787200	2.32649700	0.20140600
C	2.41894600	0.00630300	0.26005600
H	2.96066800	-0.93414400	0.12169200
H	3.02185600	0.80726700	-0.17795600
H	2.33989400	0.19630500	1.33496900
H	0.86699300	2.15551500	-0.36304600

R26

C	0.94908200	-0.37004700	0.71278900
C	-0.45919900	-0.95500800	0.74108500
C	-1.39422000	-0.34762900	-0.32133200
C	-1.37990200	1.18758500	-0.22961100
C	0.04163700	1.75520000	-0.34545000
C	0.97687400	1.15598800	0.71299400
H	-0.99958700	-0.62552000	-1.30662200
H	-0.39981000	-2.04141000	0.62234600
H	-0.87304400	-0.76828500	1.74009900
H	1.56590700	-0.77193400	1.51972400
H	-2.01776100	1.60947800	-1.01334400
H	0.02715100	2.84469900	-0.24741000
H	2.00879000	1.48360600	0.56591900
H	0.67392700	1.48941700	1.71286400

H	-1.82127600	1.49379300	0.72925500
H	0.43918200	1.53666000	-1.34238400
O	1.56562700	-0.88064000	-0.53315200
O	2.85719100	-0.63440700	-0.58360900
C	-2.81048300	-0.91683800	-0.19658600
H	-2.81176900	-2.00679100	-0.29030400
H	-3.25064400	-0.66457900	0.77435100
H	-3.46633900	-0.51426200	-0.97372900

TS26

C	-1.54191100	0.96906300	-0.45992500
C	-0.24056700	1.75659000	-0.65140000
C	-0.08271800	-0.81953800	0.62285000
C	-1.28206100	-0.54006200	-0.30871100
H	0.29858100	1.37323100	-1.52492400
H	-2.05239900	1.33725200	0.44026200
H	-2.22391100	1.13900700	-1.29893400
H	0.83571300	-1.36207700	-0.14771600
H	-0.21774000	-1.65131000	1.31613300
H	-1.04081100	-0.92775100	-1.30729000
O	2.49349900	-0.36238800	-0.20390100
O	1.99855900	-1.40303200	-0.74649800
C	0.64344400	1.62572900	0.59324300
H	0.21143600	2.21811400	1.41491100
C	0.74595400	0.21104700	1.07424900
H	1.36969500	0.03733700	1.94425600
H	1.64330400	2.03550000	0.42244500
H	-0.45194000	2.81128100	-0.84837300
C	-2.53299600	-1.28189700	0.17741800
H	-3.37808000	-1.10585400	-0.49463600
H	-2.36280400	-2.36157400	0.22241400
H	-2.82238900	-0.94538900	1.17830400

P26

C	-1.91554500	0.15073900	0.26135300
C	-1.01087200	1.34305300	0.07827300
C	0.28388700	1.24711300	-0.22558300
C	1.02669200	-0.06108700	-0.38594000
C	0.19875900	-1.23208500	0.17100300
C	-1.27005400	-1.14222800	-0.25772700
H	-2.16859700	0.04586100	1.32596300
H	-2.86888000	0.33102500	-0.24889700
H	1.17611300	-0.23100300	-1.46342200
H	0.63728400	-2.18222400	-0.15134600
H	0.25582200	-1.21481300	1.26718400
H	-1.32576200	-1.15749900	-1.35276500

H	-1.82839600	-2.01363500	0.09760400
H	-1.45787200	2.32649700	0.20140600
C	2.41894600	0.00630300	0.26005600
H	2.96066800	-0.93414400	0.12169200
H	3.02185600	0.80726700	-0.17795600
H	2.33989400	0.19630500	1.33496900
H	0.86699300	2.15551500	-0.36304600

R27

C	0.94908200	-0.37004700	0.71278900
C	-0.45919900	-0.95500800	0.74108500
C	-1.39422000	-0.34762900	-0.32133200
C	-1.37990200	1.18758500	-0.22961100
C	0.04163700	1.75520000	-0.34545000
C	0.97687400	1.15598800	0.71299400
H	-0.99958700	-0.62552000	-1.30662200
H	-0.39981000	-2.04141000	0.62234600
H	-0.87304400	-0.76828500	1.74009900
H	1.56590700	-0.77193400	1.51972400
H	-2.01776100	1.60947800	-1.01334400
H	0.02715100	2.84469900	-0.24741000
H	2.00879000	1.48360600	0.56591900
H	0.67392700	1.48941700	1.71286400
H	-1.82127600	1.49379300	0.72925500
H	0.43918200	1.53666000	-1.34238400
O	1.56562700	-0.88064000	-0.53315200
O	2.85719100	-0.63440700	-0.58360900
C	-2.81048300	-0.91683800	-0.19658600
H	-2.81176900	-2.00679100	-0.29030400
H	-3.25064400	-0.66457900	0.77435100
H	-3.46633900	-0.51426200	-0.97372900

TS27

C	1.42931700	1.17668800	-0.18888700
C	1.33504700	-0.35203200	-0.31731000
C	-1.01448100	1.10334500	0.57626000
C	0.04820200	1.84591800	-0.24996000
H	0.77988400	-0.58321500	-1.23594300
H	1.91323200	1.41611600	0.76740700
H	2.07244100	1.58439200	-0.97531500
H	-1.87303800	0.62987300	-0.30105300
H	-1.69990000	1.73287200	1.14412200
H	-0.27250600	1.91359800	-1.29594700
H	0.12129700	2.87723400	0.10734500
O	-2.02647100	-1.30034500	-0.28130500

O	-2.49572700	-0.32215900	-0.94880000
C	0.52906500	-0.90466800	0.86844600
H	1.14612000	-0.84733800	1.78010300
C	-0.73335100	-0.14478400	1.13620800
H	-1.35816700	-0.51064400	1.94345700
C	2.71283500	-1.01188400	-0.41134000
H	3.31731500	-0.78543800	0.47376500
H	2.62849200	-2.09991500	-0.48825000
H	3.26082700	-0.65659800	-1.28851700
H	0.30178100	-1.96639700	0.72916100

P27

C	1.29462600	-1.22880100	-0.10975700
C	1.92065000	0.14097700	-0.05216900
C	1.21312300	1.26511400	0.05644000
C	-0.29372200	1.29921800	0.10331600
C	-0.93657700	-0.02986100	-0.33123500
C	-0.18252200	-1.20605200	0.30899900
H	1.39143200	-1.63135600	-1.12808000
H	1.85693700	-1.91832600	0.53067700
H	-0.62224000	1.55189100	1.12278100
H	-0.66330300	2.11397200	-0.53133000
H	-0.81379700	-0.11329500	-1.42000100
H	-0.24847600	-1.11358100	1.40078700
H	-0.66411800	-2.15327800	0.04443500
H	3.00532900	0.19351200	-0.10122800
C	-2.43408700	-0.06306300	-0.01527600
H	-2.95667500	0.78300500	-0.47223400
H	-2.89817500	-0.98165000	-0.38647400
H	-2.60660100	-0.01549800	1.06536800
H	1.73073700	2.21941400	0.11339100

R28

C	-1.23677300	0.36356000	0.59526000
C	-0.27029100	1.54042600	0.51147900
C	0.99096300	1.20850800	-0.29896500
C	1.69152100	-0.06601600	0.20435900
C	0.69963700	-1.24220600	0.20565500
C	-0.56523100	-0.93979800	1.02005800
H	0.71936600	1.07360100	-1.35242100
H	-0.78881100	2.40449200	0.08616000
H	0.00577100	1.80978700	1.53753200
H	-2.09354600	0.58890000	1.23420300
H	1.18208900	-2.14103600	0.60315700
H	-1.29444000	-1.74907300	0.93459300

H	-0.31413800	-0.84749900	2.08355200
H	1.68208900	2.05688200	-0.26061000
H	2.00216600	0.11124800	1.24496100
H	0.41818000	-1.47154500	-0.82898800
O	-1.79948900	0.21601100	-0.76639900
O	-2.80539900	-0.63144600	-0.79826400
C	2.94412300	-0.38352000	-0.61679600
H	3.45253300	-1.27391300	-0.23537100
H	2.68698000	-0.56987100	-1.66474500
H	3.65716800	0.44578300	-0.59101300

TS28

C	-1.71122100	0.01654100	0.17488500
C	-0.90319900	1.10957000	-0.54243500
C	0.58860000	-0.80560200	1.00080300
C	-0.80696500	-1.19167900	0.48292300
H	-0.48000200	0.70174300	-1.46847300
H	-2.04611000	0.43612900	1.13428700
H	1.42578100	-1.19141600	0.06326400
H	0.97497600	-1.41488700	1.81819500
H	-0.71378700	-1.79954300	-0.42601300
H	-1.29703200	-1.83321900	1.22184300
O	2.46192800	0.25558900	-0.69999900
O	2.31538300	-0.99717700	-0.87791500
C	0.22589500	1.63070000	0.35276200
H	-0.20071000	2.23499800	1.16850700
C	1.01300800	0.52506000	0.98526600
H	1.84915800	0.81671100	1.61104200
H	0.89553500	2.29989300	-0.19560000
C	-2.95021500	-0.40394000	-0.61954000
H	-3.62532900	0.44207000	-0.77814700
H	-2.66851500	-0.79581100	-1.60256700
H	-3.51088500	-1.18582400	-0.09843700
H	-1.55698700	1.93795100	-0.83257100

P28

C	1.29462600	-1.22880100	-0.10975700
C	1.92065000	0.14097700	-0.05216900
C	1.21312300	1.26511400	0.05644000
C	-0.29372200	1.29921800	0.10331600
C	-0.93657700	-0.02986100	-0.33123500
C	-0.18252200	-1.20605200	0.30899900
H	1.39143200	-1.63135600	-1.12808000
H	1.85693700	-1.91832600	0.53067700
H	-0.62224000	1.55189100	1.12278100
H	-0.66330300	2.11397200	-0.53133000

H	-0.81379700	-0.11329500	-1.42000100
H	-0.24847600	-1.11358100	1.40078700
H	-0.66411800	-2.15327800	0.04443500
H	3.00532900	0.19351200	-0.10122800
C	-2.43408700	-0.06306300	-0.01527600
H	-2.95667500	0.78300500	-0.47223400
H	-2.89817500	-0.98165000	-0.38647400
H	-2.60660100	-0.01549800	1.06536800
H	1.73073700	2.21941400	0.11339100

R29

C	0.54742700	1.27452700	-0.65424700
C	-0.66170600	2.14842300	-0.28981700
C	-1.92870800	1.30538300	-0.09514900
C	-1.70684600	0.19324600	0.93841800
C	-0.47817800	-0.65585600	0.62016000
C	0.79679500	0.16508100	0.38684700
H	-2.76556100	1.93684500	0.21785200
H	-0.44505800	2.69946500	0.63494100
H	-0.82507400	2.90091700	-1.06752900
H	0.38387800	0.81285300	-1.63494500
H	1.43995200	1.89903600	-0.74679200
H	-1.55786400	0.62881000	1.93376400
H	-2.57532200	-0.46680500	1.00505400
H	-0.32000600	-1.41734000	1.38801600
H	0.99825700	0.65237200	1.35228200
H	-2.21674200	0.85529200	-1.05132400
C	2.00199100	-0.73643100	0.05884300
H	1.85503700	-1.18894200	-0.92670700
H	2.01636300	-1.56764800	0.77407000
O	-0.72325300	-1.41324300	-0.62706200
O	-1.61182900	-2.36933400	-0.45691600
C	3.35598600	-0.02128000	0.10666000
H	4.17191600	-0.72723100	-0.06859600
H	3.43535200	0.76104800	-0.65213900
H	3.52497000	0.44338300	1.08358500

TS29

C	-0.69092500	2.15661100	-0.34334900
C	0.47282800	1.23326700	-0.72565000
C	-1.68157000	0.09057100	0.80330800
C	-1.96345300	1.36016300	-0.01756200
H	0.19347500	0.61414600	-1.58693900
H	1.33864700	1.82717700	-1.02875200
H	-0.39576100	2.74897900	0.53160500

H	-0.89521700	2.86977800	-1.14729400
H	-1.99892500	-0.90144500	0.02375200
H	-2.38817700	-0.11933000	1.60731100
H	-2.46832100	1.09072200	-0.95246200
H	-2.66751600	1.99252900	0.53091600
C	1.99533100	-0.66765400	0.14793600
H	2.07886100	-1.36897500	0.98591400
H	1.72544300	-1.26988900	-0.72294700
O	-0.54130500	-2.12448700	-0.37205600
O	-1.76466700	-1.98207400	-0.69552500
C	0.84946200	0.31906300	0.45070900
H	1.20941200	0.97287500	1.26699900
C	-0.37091700	-0.34295400	1.02934900
H	-0.19213800	-1.05776300	1.82646400
C	3.35278000	0.00565000	-0.07681100
H	4.13279000	-0.74364100	-0.23495500
H	3.34794800	0.65799400	-0.95376800
H	3.64603900	0.61103200	0.78722700

P29

C	2.37655500	-0.18579500	-0.26435100
C	1.74222800	1.16892400	-0.07957600
C	0.45662100	1.34463000	0.22647700
C	-0.54946700	0.22529800	0.38286500
C	0.01648700	-1.09555900	-0.16988300
C	1.47333300	-1.31108800	0.25820100
H	2.59984400	-0.34264500	-1.32922300
H	3.34718500	-0.21034800	0.24455700
H	-0.73908300	0.09211200	1.46037000
H	-0.59942600	-1.93789400	0.15710900
H	-0.03766000	-1.07140300	-1.26647400
H	1.52482900	-1.33357100	1.35335300
H	1.83555600	-2.28220700	-0.09294400
H	2.38498300	2.03761300	-0.19942100
C	-1.89602000	0.61622000	-0.26188300
H	-2.19619600	1.59424300	0.13225400
H	-1.74384500	0.75672100	-1.33913600
H	0.07850300	2.35528600	0.36767200
C	-3.03106300	-0.38502200	-0.02518000
H	-3.97027300	-0.01587500	-0.44600000
H	-2.82515700	-1.35397600	-0.48688900
H	-3.19130600	-0.55371200	1.04474700

R30

C	1.42690300	-0.35968900	0.68994400

C	-0.00078700	-0.82428200	0.95563400
C	-1.03649100	-0.21925100	-0.01159500
C	-0.89786400	1.31354700	-0.04100600
C	0.53037200	1.75495100	-0.39143700
C	1.56343900	1.15522300	0.57041200
H	-0.80948600	-0.59611500	-1.01784800
H	-0.03517900	-1.91744200	0.91438600
H	-0.25293800	-0.53909500	1.98492300
H	2.12234600	-0.75786000	1.43228000
H	-1.59990700	1.74348500	-0.76054800
H	0.60488400	2.84638100	-0.37521400
H	2.58407600	1.38833200	0.25770400
H	1.43104600	1.57693000	1.57404000
H	-1.17506100	1.71517400	0.94417100
H	0.76618200	1.44199500	-1.41427100
O	1.82043400	-0.99908400	-0.58668600
O	3.10540100	-0.86170200	-0.83419900
C	-2.45337000	-0.69100700	0.35825200
H	-2.43371300	-1.78001700	0.48442900
H	-2.72143600	-0.27693600	1.33905600
C	-3.53673100	-0.33311600	-0.66462200
H	-3.66670800	0.74730700	-0.76582200
H	-3.29059000	-0.73279400	-1.65339900
H	-4.50303500	-0.75131700	-0.37029900

TS30

C	-0.95432800	1.27216900	-0.35660200
C	0.45288500	1.78967600	-0.67931600
C	0.23749500	-0.71297200	0.71001000
C	-0.97178100	-0.25154000	-0.13231100
H	0.84216900	1.27502100	-1.56489600
H	-1.31858700	1.77312500	0.55108700
H	-1.64601100	1.53755600	-1.16037700
H	0.97037300	-1.43870000	-0.10514600
H	0.01469700	-1.48353100	1.45007800
H	-0.90620600	-0.72612400	-1.12155500
O	2.77587700	-0.77229200	-0.33061800
O	2.05332600	-1.71620800	-0.78838300
C	1.39399300	1.54913200	0.50484600
H	1.14192500	2.24198500	1.32291200
C	1.27564100	0.15871200	1.04756300
H	1.92794900	-0.09972700	1.87444400
H	2.43473000	1.76100800	0.24260300
H	0.42377300	2.85577700	-0.92127900
C	-2.28096600	-0.74228600	0.51425000

H	-2.19594000	-1.82113700	0.68974400
H	-2.38099600	-0.27942900	1.50430500
C	-3.54137600	-0.47321200	-0.31332900
H	-4.42120900	-0.91018300	0.16635600
H	-3.73224800	0.59605400	-0.43403500
H	-3.45742000	-0.91176700	-1.31290200

P30

C	2.37655500	-0.18579500	-0.26435100
C	1.74222800	1.16892400	-0.07957600
C	0.45662100	1.34463000	0.22647700
C	-0.54946700	0.22529800	0.38286500
C	0.01648700	-1.09555900	-0.16988300
C	1.47333300	-1.31108800	0.25820100
H	2.59984400	-0.34264500	-1.32922300
H	3.34718500	-0.21034800	0.24455700
H	-0.73908300	0.09211200	1.46037000
H	-0.59942600	-1.93789400	0.15710900
H	-0.03766000	-1.07140300	-1.26647400
H	1.52482900	-1.33357100	1.35335300
H	1.83555600	-2.28220700	-0.09294400
H	2.38498300	2.03761300	-0.19942100
C	-1.89602000	0.61622000	-0.26188300
H	-2.19619600	1.59424300	0.13225400
H	-1.74384500	0.75672100	-1.33913600
H	0.07850300	2.35528600	0.36767200
C	-3.03106300	-0.38502200	-0.02518000
H	-3.97027300	-0.01587500	-0.44600000
H	-2.82515700	-1.35397600	-0.48688900
H	-3.19130600	-0.55371200	1.04474700

R31

C	1.42690300	-0.35968900	0.68994400
C	-0.00078700	-0.82428200	0.95563400
C	-1.03649100	-0.21925100	-0.01159500
C	-0.89786400	1.31354700	-0.04100600
C	0.53037200	1.75495100	-0.39143700
C	1.56343900	1.15522300	0.57041200
H	-0.80948600	-0.59611500	-1.01784800
H	-0.03517900	-1.91744200	0.91438600
H	-0.25293800	-0.53909500	1.98492300
H	2.12234600	-0.75786000	1.43228000
H	-1.59990700	1.74348500	-0.76054800
H	0.60488400	2.84638100	-0.37521400
H	2.58407600	1.38833200	0.25770400

H	1.43104600	1.57693000	1.57404000
H	-1.17506100	1.71517400	0.94417100
H	0.76618200	1.44199500	-1.41427100
O	1.82043400	-0.99908400	-0.58668600
O	3.10540100	-0.86170200	-0.83419900
C	-2.45337000	-0.69100700	0.35825200
H	-2.43371300	-1.78001700	0.48442900
H	-2.72143600	-0.27693600	1.33905600
C	-3.53673100	-0.33311600	-0.66462200
H	-3.66670800	0.74730700	-0.76582200
H	-3.29059000	-0.73279400	-1.65339900
H	-4.50303500	-0.75131700	-0.37029900

TS31

C	0.91806600	1.36911600	0.00565500
C	1.00456200	-0.16411600	0.08705600
C	-1.60889600	1.08862500	0.32446000
C	-0.48499100	1.84310100	-0.40345900
H	0.65949400	-0.57873500	-0.87039000
H	1.17334700	1.78212700	0.99072400
H	1.65293700	1.76485900	-0.70024700
H	-2.23339200	0.40183100	-0.60789900
H	-2.44873800	1.69598900	0.66273200
H	-0.59950300	1.72960500	-1.48772900
H	-0.58531700	2.91380100	-0.20283400
O	-2.18966500	-1.51389900	-0.33002000
O	-2.62100800	-0.69575300	-1.20606600
C	0.04301900	-0.65394500	1.18125600
H	0.46390200	-0.40427700	2.16899100
C	-1.31632900	-0.03116300	1.10614600
H	-2.04465200	-0.36607100	1.83651600
C	2.42981900	-0.68199300	0.34160700
H	2.82912000	-0.19737100	1.24216800
H	2.37376200	-1.75216600	0.57343100
H	-0.04698800	-1.74482000	1.16359600
C	3.40189600	-0.48077800	-0.82548500
H	3.55102300	0.57656500	-1.05905000
H	4.38261300	-0.90436500	-0.59327600
H	3.03489900	-0.97283800	-1.73147100

P31

C	1.94112900	-1.02486500	-0.24460400
C	2.31830300	0.43130400	-0.15589900
C	1.43505300	1.39952600	0.08500300
C	-0.04394500	1.15965400	0.25942200
C	-0.48194300	-0.24624300	-0.19228900

C	0.53319600	-1.28730600	0.30729800
H	2.00480600	-1.35602900	-1.29086200
H	2.67676400	-1.63016900	0.29791300
H	-0.31683900	1.30865300	1.31537800
H	-0.59767100	1.92497800	-0.29540100
H	-0.45792200	-0.26226000	-1.29183700
H	0.56323900	-1.25262900	1.40388900
H	0.20403500	-2.29533500	0.03399200
H	3.36638300	0.68396500	-0.29496900
C	-1.91160700	-0.59419600	0.25262600
H	-2.12659600	-1.62562700	-0.05085100
H	-1.95336900	-0.58602900	1.34961200
H	1.77722500	2.42892800	0.15745400
C	-3.00251800	0.32547000	-0.30711300
H	-3.99576200	-0.01808400	-0.00545200
H	-2.89238800	1.35407200	0.04596400
H	-2.97791100	0.34550300	-1.40149300

R32

C	-1.67016200	0.39116700	0.58307900
C	-0.61448100	1.49067900	0.59712200
C	0.65742600	1.10076900	-0.17154700
C	1.23843000	-0.24418300	0.30156800
C	0.15825700	-1.33666200	0.20166200
C	-1.11801800	-0.97674900	0.97333100
H	0.42579000	1.03070300	-1.24098900
H	-1.04527200	2.41045400	0.19088600
H	-0.36828300	1.69301200	1.64595500
H	-2.53611200	0.65377900	1.19489300
H	0.55240000	-2.28719800	0.57549200
H	-1.89962600	-1.72406200	0.81637100
H	-0.91096600	-0.95188100	2.04994300
H	1.39513400	1.90035900	-0.06278000
H	1.51188300	-0.13886100	1.36321100
H	-0.09023200	-1.49653700	-0.85424100
O	-2.17908600	0.34727400	-0.80660700
O	-3.24179600	-0.42016000	-0.92109700
C	2.50624900	-0.64927900	-0.46944600
H	2.78705900	-1.66354400	-0.16195200
H	2.26570400	-0.71286600	-1.53821700
C	3.70940100	0.27839500	-0.26776200
H	3.52115400	1.28536700	-0.64853500
H	3.96611800	0.36616000	0.79291000
H	4.58970600	-0.10663300	-0.78935900

TS32

C	-1.25882200	-0.18244500	0.32106900
C	-0.58781200	1.02033200	-0.36191100
C	1.15225300	-0.86855400	0.93069500
C	-0.24085900	-1.32863600	0.47097600
H	-0.19606100	0.71002300	-1.33824700
H	-1.55409300	0.13640500	1.33197300
H	1.95365100	-1.11120500	-0.08376000
H	1.63895600	-1.50342200	1.67147200
H	-0.16361000	-1.85624700	-0.48830400
H	-0.62612600	-2.06342500	1.18481000
O	2.82281300	0.46943400	-0.78876200
O	2.76171000	-0.77429300	-1.05750800
C	0.55635400	1.56792500	0.49876000
H	0.14063300	2.07732200	1.38214400
C	1.46887600	0.49018900	0.99588200
H	2.31876300	0.80444200	1.59146500
H	1.13303000	2.32646800	-0.03891900
C	-2.52307500	-0.66974400	-0.40692600
H	-2.26791500	-0.88744700	-1.45183700
H	-2.83025100	-1.62581600	0.03365200
H	-1.31132000	1.81740400	-0.55055300
C	-3.70863800	0.29960400	-0.35632400
H	-3.48444600	1.25049900	-0.84655000
H	-4.58186300	-0.12602700	-0.85785200
H	-3.99519200	0.51786500	0.67733700

P32

C	1.94112900	-1.02486500	-0.24460400
C	2.31830300	0.43130400	-0.15589900
C	1.43505300	1.39952600	0.08500300
C	-0.04394500	1.15965400	0.25942200
C	-0.48194300	-0.24624300	-0.19228900
C	0.53319600	-1.28730600	0.30729800
H	2.00480600	-1.35602900	-1.29086200
H	2.67676400	-1.63016900	0.29791300
H	-0.31683900	1.30865300	1.31537800
H	-0.59767100	1.92497800	-0.29540100
H	-0.45792200	-0.26226000	-1.29183700
H	0.56323900	-1.25262900	1.40388900
H	0.20403500	-2.29533500	0.03399200
H	3.36638300	0.68396500	-0.29496900
C	-1.91160700	-0.59419600	0.25262600
H	-2.12659600	-1.62562700	-0.05085100
H	-1.95336900	-0.58602900	1.34961200

H	1.77722500	2.42892800	0.15745400
C	-3.00251800	0.32547000	-0.30711300
H	-3.99576200	-0.01808400	-0.00545200
H	-2.89238800	1.35407200	0.04596400
H	-2.97791100	0.34550300	-1.40149300

R33

C	-0.82882500	0.67908400	0.62567500
C	0.31600200	-0.31913200	0.41030600
C	-0.07599300	-1.37889600	-0.63976400
C	-1.40782000	-2.06434400	-0.30065500
C	-2.53953300	-1.04373400	-0.12713400
C	-2.17553500	0.02050900	0.91705400
H	-0.15101600	-0.89701500	-1.62161000
H	0.42626400	-0.83284700	1.37665600
H	-0.57437000	1.40601000	1.40093300
H	-1.66579600	-2.78358200	-1.08411200
H	-3.46747600	-1.54415300	0.16506600
H	-2.93711200	0.80247200	0.97089000
H	-2.11204900	-0.43498600	1.91257500
H	0.71486500	-2.12938500	-0.71844500
H	-1.29286400	-2.64187900	0.62605300
H	-2.73618500	-0.55280600	-1.08628800
O	-0.93755100	1.46882000	-0.62083100
O	-1.67629500	2.54618600	-0.45936800
C	1.64559700	0.39593800	0.10626200
H	1.77671200	1.20878500	0.83226500
H	1.58158400	0.87620000	-0.87602300
C	2.88489600	-0.50662300	0.15791800
H	2.80762200	-1.29299000	-0.59982900
H	2.92023800	-1.01801900	1.12809900
C	4.18870100	0.26794000	-0.05710500
H	4.32298800	1.03818600	0.70869600
H	4.19532300	0.76668200	-1.03107500
H	5.05710300	-0.39516800	-0.01759400

TS33

C	1.44784500	-2.06355100	-0.36659400
C	0.13943600	-1.33884400	-0.70517700
C	2.13661900	0.12708900	0.76546900
C	2.58640600	-1.07270000	-0.08522600
H	0.28581200	-0.68573000	-1.57428100
H	-0.63056800	-2.06411600	-0.97944600
H	1.28267100	-2.69220100	0.51715100
H	1.73434700	-2.73742100	-1.17932800

H	2.27173100	1.16717900	-0.00504000
H	2.82622800	0.43486100	1.55253100
H	2.99735900	-0.71749100	-1.03734900
H	3.40861100	-1.58472300	0.42294700
C	-1.62918100	0.30714000	0.22112000
H	-1.78978900	0.99797200	1.05808100
H	-1.48508500	0.93526800	-0.66262600
O	0.62922800	2.15023700	-0.34003200
O	1.84998900	2.20610600	-0.69821600
C	-0.33863000	-0.49330200	0.48657500
H	-0.56983400	-1.19578900	1.30884600
C	0.78184300	0.34670100	1.03635500
H	0.51895100	1.01666600	1.84888800
C	-2.88443200	-0.55672100	0.04821500
H	-2.76328600	-1.22781200	-0.80881300
H	-3.00559900	-1.20199400	0.92746900
C	-4.15260800	0.27944800	-0.14827400
H	-5.03419200	-0.35573600	-0.26987700
H	-4.33107700	0.93587400	0.70908500
H	-4.07380000	0.91288700	-1.03702600

P33

C	-2.88358200	-0.15031600	0.36534900
C	-2.23607400	1.19068200	0.13288300
C	-0.96841600	1.33986400	-0.25190600
C	0.00488400	0.20012800	-0.46235800
C	-0.55176900	-1.10632000	0.13288100
C	-2.03585600	-1.29562000	-0.20446200
H	-3.04335100	-0.29698900	1.44307500
H	-3.88421600	-0.15885300	-0.08228200
H	0.12391900	0.05779600	-1.54865200
H	0.02682200	-1.96220600	-0.22545600
H	-0.43112100	-1.07743000	1.22397200
H	-2.15447300	-1.32248000	-1.29426400
H	-2.39438400	-2.25775900	0.17418800
H	-2.85343600	2.07241000	0.28648400
C	1.39507500	0.56980800	0.09572300
H	1.68861900	1.54127300	-0.32232400
H	1.31372600	0.71896700	1.18042400
H	-0.58023700	2.34218300	-0.42196500
C	2.50555700	-0.44572400	-0.19978800
H	2.26833600	-1.40654700	0.26866700
H	2.54379900	-0.63292200	-1.28021500
C	3.88122000	0.02211500	0.28536800
H	3.88172700	0.19277900	1.36651400

H	4.65536700	-0.71844100	0.06627000
H	4.17267100	0.96052300	-0.19657600

R34

C	1.87809700	-0.41490400	0.68489100
C	0.42077500	-0.74945100	0.98321500
C	-0.57712800	-0.07748700	0.01983700
C	-0.30717100	1.43681200	-0.04609600
C	1.14767600	1.74590900	-0.42801200
C	2.14180900	1.07989900	0.53126800
H	-0.40072800	-0.49316500	-0.98106800
H	0.29214700	-1.83622100	0.96637000
H	0.21301500	-0.42294300	2.00996700
H	2.54970000	-0.85678700	1.42446300
H	-0.98140300	1.91037400	-0.76484900
H	1.31574600	2.82693800	-0.43808500
H	3.17337900	1.21874100	0.19892400
H	2.06216200	1.53166500	1.52747300
H	-0.53245900	1.88186600	0.93353000
H	1.33788500	1.39151400	-1.44681900
O	2.19321600	-1.11075900	-0.58387300
O	3.48086300	-1.09024100	-0.85376100
C	-2.02127400	-0.41867100	0.42485000
H	-2.09236300	-1.50309700	0.57939500
H	-2.23756900	0.04003900	1.39975500
C	-3.09981000	-0.00181400	-0.58325100
H	-3.09135400	1.08510200	-0.71502100
H	-2.85903900	-0.43105500	-1.56334800
C	-4.50548200	-0.44356800	-0.16444600
H	-4.78691800	-0.00595800	0.79848700
H	-4.56462900	-1.53165000	-0.06243500
H	-5.25515900	-0.13770800	-0.89920000

TS34

C	-0.32395400	1.44208600	-0.36490600
C	1.14073300	1.79832200	-0.64702400
C	0.60077600	-0.68630500	0.68826700
C	-0.52103100	-0.07430500	-0.17994500
H	1.48682300	1.25906200	-1.53591200
H	-0.64900800	1.96081900	0.54754100
H	-0.96113700	1.80400000	-1.17603200
H	1.26334300	-1.48602900	-0.11768200
H	0.26894000	-1.43249200	1.41221100
H	-0.47159100	-0.53058800	-1.17862100
O	3.13771900	-1.02959400	-0.29909500

O	2.32125500	-1.87919700	-0.78292200
C	2.02120900	1.42696700	0.54951400
H	1.83720900	2.13086200	1.37617500
C	1.72577000	0.05229100	1.06345900
H	2.32207700	-0.29369300	1.90069200
H	3.08511100	1.51660300	0.31103900
H	1.24152000	2.86499900	-0.86627600
C	-1.89839600	-0.43084300	0.40934500
H	-1.93935000	-1.51685200	0.56253000
H	-1.98715200	0.02024100	1.40692100
C	-3.09851800	-0.01477300	-0.44978900
H	-3.12030100	1.07442600	-0.55917500
H	-2.97437300	-0.42179700	-1.46092700
C	-4.43523300	-0.48788100	0.12951200
H	-5.27419200	-0.17791200	-0.49930900
H	-4.46712700	-1.57871600	0.21237800
H	-4.60072400	-0.07597700	1.12999400

P34

C	-2.88358200	-0.15031600	0.36534900
C	-2.23607400	1.19068200	0.13288300
C	-0.96841600	1.33986400	-0.25190600
C	0.00488400	0.20012800	-0.46235800
C	-0.55176900	-1.10632000	0.13288100
C	-2.03585600	-1.29562000	-0.20446200
H	-3.04335100	-0.29698900	1.44307500
H	-3.88421600	-0.15885300	-0.08228200
H	0.12391900	0.05779600	-1.54865200
H	0.02682200	-1.96220600	-0.22545600
H	-0.43112100	-1.07743000	1.22397200
H	-2.15447300	-1.32248000	-1.29426400
H	-2.39438400	-2.25775900	0.17418800
H	-2.85343600	2.07241000	0.28648400
C	1.39507500	0.56980800	0.09572300
H	1.68861900	1.54127300	-0.32232400
H	1.31372600	0.71896700	1.18042400
H	-0.58023700	2.34218300	-0.42196500
C	2.50555700	-0.44572400	-0.19978800
H	2.26833600	-1.40654700	0.26866700
H	2.54379900	-0.63292200	-1.28021500
C	3.88122000	0.02211500	0.28536800
H	3.88172700	0.19277900	1.36651400
H	4.65536700	-0.71844100	0.06627000
H	4.17267100	0.96052300	-0.19657600

R35

C	1.87809700	-0.41490400	0.68489100
C	0.42077500	-0.74945100	0.98321500
C	-0.57712800	-0.07748700	0.01983700
C	-0.30717100	1.43681200	-0.04609600
C	1.14767600	1.74590900	-0.42801200
C	2.14180900	1.07989900	0.53126800
H	-0.40072800	-0.49316500	-0.98106800
H	0.29214700	-1.83622100	0.96637000
H	0.21301500	-0.42294300	2.00996700
H	2.54970000	-0.85678700	1.42446300
H	-0.98140300	1.91037400	-0.76484900
H	1.31574600	2.82693800	-0.43808500
H	3.17337900	1.21874100	0.19892400
H	2.06216200	1.53166500	1.52747300
H	-0.53245900	1.88186600	0.93353000
H	1.33788500	1.39151400	-1.44681900
O	2.19321600	-1.11075900	-0.58387300
O	3.48086300	-1.09024100	-0.85376100
C	-2.02127400	-0.41867100	0.42485000
H	-2.09236300	-1.50309700	0.57939500
H	-2.23756900	0.04003900	1.39975500
C	-3.09981000	-0.00181400	-0.58325100
H	-3.09135400	1.08510200	-0.71502100
H	-2.85903900	-0.43105500	-1.56334800
C	-4.50548200	-0.44356800	-0.16444600
H	-4.78691800	-0.00595800	0.79848700
H	-4.56462900	-1.53165000	-0.06243500
H	-5.25515900	-0.13770800	-0.89920000

TS35

C	0.29210200	1.52433600	-0.02144900
C	0.54338700	0.01718500	0.15569000
C	-2.20024800	0.97918900	0.21957100
C	-1.13593000	1.81554300	-0.50840200
H	0.28651300	-0.48673100	-0.78620700
H	0.45784200	2.01722500	0.94582600
H	1.00872400	1.96028000	-0.72234800
H	-2.69758800	0.17266900	-0.69271500
H	-3.11822600	1.50398700	0.48512100
H	-1.19107400	1.63374400	-1.58801400
H	-1.36169300	2.87730100	-0.37246100
O	-2.45963400	-1.70662900	-0.29072200
O	-2.93401900	-0.99511500	-1.23504100
C	-0.40497700	-0.51333600	1.24281800

H	-0.05574000	-0.16199700	2.22761700
C	-1.82018400	-0.05269800	1.08036100
H	-2.53816100	-0.42559800	1.80249900
C	2.00381700	-0.32392500	0.49437300
H	2.31791000	0.26840200	1.36501500
H	2.05248100	-1.37383000	0.81068000
H	-0.37443500	-1.60653400	1.28914300
C	3.00328100	-0.11700600	-0.65074100
H	2.99752700	0.93035200	-0.97072200
H	2.67820000	-0.70358300	-1.51836100
C	4.43232300	-0.51724700	-0.27137500
H	5.12290300	-0.36943600	-1.10596000
H	4.79879200	0.07644300	0.57196000
H	4.48381700	-1.57097800	0.01995000

P35

C	2.44786400	-1.05449600	-0.23013200
C	2.85657400	0.39293800	-0.13692000
C	1.99215800	1.38060300	0.09305500
C	0.50600000	1.17383400	0.24868500
C	0.04211700	-0.22203900	-0.20831000
C	1.02791500	-1.28519000	0.30441900
H	2.51715000	-1.38740900	-1.27550000
H	3.16301100	-1.67589600	0.32154000
H	0.22293200	1.32956200	1.30092900
H	-0.02324000	1.95099800	-0.31347800
H	0.08067700	-0.23958400	-1.30731100
H	1.04499000	-1.25040100	1.40126900
H	0.67977000	-2.28578500	0.02742100
H	3.91168700	0.62201200	-0.26309300
C	-1.39967700	-0.53993600	0.21879200
H	-1.63254700	-1.56895300	-0.08424900
H	-1.45800800	-0.52766500	1.31603800
H	2.35603900	2.40223400	0.16978800
C	-2.47482300	0.39245100	-0.35450600
H	-2.30361400	1.41796300	-0.01060600
H	-2.38358900	0.41722200	-1.44750900
C	-3.89582600	-0.03306400	0.02806000
H	-4.02673800	-0.04084400	1.11474800
H	-4.64335100	0.64609900	-0.39101300
H	-4.11898600	-1.04014500	-0.33783500

R36

C	-2.20007600	0.39314400	0.49020100
C	-1.17492500	1.52005900	0.53599100

C	0.16510700	1.12953400	-0.10696000
C	0.73644400	-0.17584500	0.47506000
C	-0.30703200	-1.30060300	0.34328600
C	-1.65058600	-0.93913500	0.99065200
H	0.02536300	1.00497400	-1.18728000
H	-1.59200900	2.40811500	0.05234300
H	-1.02063300	1.77728500	1.59037600
H	-3.11915300	0.66043100	1.01651200
H	0.07707900	-2.22134300	0.79408000
H	-2.39900500	-1.71399500	0.80759200
H	-1.53356500	-0.85852100	2.07807300
H	0.87199300	1.95306200	0.02408500
H	0.92197200	-0.01467100	1.54847500
H	-0.46481600	-1.51704600	-0.71996000
O	-2.59201100	0.27016000	-0.93210000
O	-3.62391100	-0.53005000	-1.09425800
C	2.06963500	-0.58350400	-0.17554000
H	2.34809700	-1.57736400	0.19773700
H	1.91732800	-0.69945500	-1.25717500
C	3.24197300	0.37488900	0.07176200
H	3.01265500	1.36316500	-0.34003000
H	3.36946900	0.51556400	1.15228200
C	4.55674800	-0.12455800	-0.53532400
H	4.47008000	-0.24531300	-1.61962500
H	4.84345300	-1.09440800	-0.11715400
H	5.37533800	0.57475700	-0.34422500
**TS36**			
C	-0.75394100	-0.09386300	0.48735400
C	-0.10506300	1.03992100	-0.32470700
C	1.67796300	-0.81978300	0.95320300
C	0.24070000	-1.26138500	0.63203400
H	0.19759300	0.65410300	-1.30581500
H	-0.95639700	0.29820200	1.49519500
H	2.39007400	-1.15452700	-0.10033000
H	2.20242500	-1.42103700	1.69627000
H	0.22787000	-1.84958600	-0.29454300
H	-0.10761700	-1.93760700	1.41900600
O	3.24657800	0.34658700	-0.97482000
O	3.12865300	-0.90912600	-1.15379200
C	1.11966000	1.60213400	0.40536500
H	0.79063900	2.18247500	1.28150800
C	2.03701300	0.52897400	0.90277500
H	2.93964700	0.85254900	1.40904600
H	1.67353300	2.30339600	-0.22589100

C	-2.08647500	-0.58386000	-0.10325200
H	-1.93180400	-0.85586100	-1.15601300
H	-2.37388600	-1.51161200	0.40821700
H	-0.81799200	1.84746400	-0.50901100
C	-3.25159900	0.40830200	0.00227000
H	-3.00845100	1.33231900	-0.53297000
H	-3.38719600	0.69082600	1.05349400
C	-4.56575400	-0.15660900	-0.54582100
H	-5.38073600	0.56621200	-0.45234000
H	-4.47317500	-0.41742600	-1.60464400
H	-4.86140500	-1.06257500	-0.00761300

P36

C	2.44786400	-1.05449600	-0.23013200
C	2.85657400	0.39293800	-0.13692000
C	1.99215800	1.38060300	0.09305500
C	0.50600000	1.17383400	0.24868500
C	0.04211700	-0.22203900	-0.20831000
C	1.02791500	-1.28519000	0.30441900
H	2.51715000	-1.38740900	-1.27550000
H	3.16301100	-1.67589600	0.32154000
H	0.22293200	1.32956200	1.30092900
H	-0.02324000	1.95099800	-0.31347800
H	0.08067700	-0.23958400	-1.30731100
H	1.04499000	-1.25040100	1.40126900
H	0.67977000	-2.28578500	0.02742100
H	3.91168700	0.62201200	-0.26309300
C	-1.39967700	-0.53993600	0.21879200
H	-1.63254700	-1.56895300	-0.08424900
H	-1.45800800	-0.52766500	1.31603800
H	2.35603900	2.40223400	0.16978800
C	-2.47482300	0.39245100	-0.35450600
H	-2.30361400	1.41796300	-0.01060600
H	-2.38358900	0.41722200	-1.44750900
C	-3.89582600	-0.03306400	0.02806000
H	-4.02673800	-0.04084400	1.11474800
H	-4.64335100	0.64609900	-0.39101300
H	-4.11898600	-1.04014500	-0.33783500

R37

C	-1.33970500	0.66002100	0.62423100
C	-0.13768100	-0.26704500	0.40249000
C	-0.47052300	-1.34729400	-0.64704000
C	-1.75611700	-2.11275900	-0.30071800
C	-2.94731200	-1.16346500	-0.11714300

C	-2.64153300	-0.07916900	0.92472900
H	-0.58073900	-0.86925200	-1.62736500
H	0.00749100	-0.77407300	1.36774400
H	-1.12517400	1.40195800	1.39736700
H	-1.97501000	-2.84531300	-1.08377400
H	-3.83977600	-1.72098600	0.18232300
H	-3.44940400	0.65436100	0.98446500
H	-2.54280600	-0.53036500	1.91934400
H	0.36461100	-2.04761800	-0.73141700
H	-1.59970100	-2.68368400	0.62403600
H	-3.18268000	-0.68596700	-1.07442500
O	-1.50360700	1.44030600	-0.62224300
O	-2.30740900	2.46957800	-0.45781100
C	1.14419000	0.52882200	0.09454400
H	1.22119900	1.35294600	0.81551400
H	1.05022000	0.99808900	-0.89055700
C	2.43784400	-0.29256000	0.15435700
H	2.41725200	-1.08425700	-0.60324300
H	2.50205300	-0.80051000	1.12605100
C	3.69767800	0.55691700	-0.05213700
H	3.73087500	1.34378400	0.71110800
H	3.62904400	1.07355200	-1.01689000
C	4.99275000	-0.25828300	-0.00013800
H	5.87036200	0.37747000	-0.14508500
H	5.10450200	-0.76285000	0.96478900
H	5.00827400	-1.02747800	-0.77860800

TS37

C	1.79485300	-2.11286300	-0.36921400
C	0.52375800	-1.31482400	-0.68626700
C	2.62892700	0.04062100	0.73799500
C	2.99417500	-1.18894700	-0.10991800
H	0.69117900	-0.67299700	-1.55996000
H	-0.29177100	-1.99500600	-0.94420900
H	1.60944200	-2.72966700	0.51888400
H	2.02778200	-2.80347700	-1.18496800
H	2.80608400	1.06452500	-0.04431800
H	3.35039800	0.31533300	1.50856600
H	3.41281300	-0.86515400	-1.06986000
H	3.79109500	-1.74618800	0.39090700
C	-1.12969700	0.43312100	0.26761300
H	-1.22583100	1.14230800	1.09886100
H	-0.97005800	1.04053400	-0.62772200
O	1.21461600	2.13578000	-0.35554800
O	2.42871000	2.12040400	-0.73936400

C	0.11763600	-0.44020200	0.51101100
H	-0.13679200	-1.12535200	1.34096100
C	1.29433700	0.33767600	1.03365300
H	1.08531700	1.02681900	1.84580400
C	-2.43797000	-0.35615000	0.13944000
H	-2.37958900	-1.04976400	-0.70748700
H	-2.57856800	-0.97750000	1.03423500
C	-3.66514100	0.54476200	-0.04455900
H	-3.73095000	1.24130000	0.79994200
H	-3.52516300	1.16459100	-0.93808400
C	-4.97663500	-0.23631600	-0.16525500
H	-5.82994200	0.43440700	-0.29734300
H	-4.95435900	-0.91739700	-1.02183600
H	-5.16314600	-0.83805300	0.72993600

P37

C	-3.39498500	0.32457400	-0.36472700
C	-2.86517200	-1.06643200	-0.12735400
C	-1.61442200	-1.32252600	0.25645900
C	-0.54538700	-0.27093100	0.46028200
C	-0.99083600	1.07906100	-0.13279900
C	-2.45333900	1.39402200	0.20450700
H	-3.53927600	0.48192800	-1.44309300
H	-4.39218200	0.42015000	0.08048700
H	-0.40780400	-0.13964400	1.54572800
H	-0.34178600	1.88295100	0.22557200
H	-0.87334700	1.04113500	-1.22404100
H	-2.56926400	1.43155400	1.29427100
H	-2.72826900	2.38288100	-0.17508200
H	-3.55669300	-1.89231200	-0.27504700
C	0.80470100	-0.75762500	-0.10665600
H	1.01192700	-1.75504200	0.30135800
H	0.70612000	-0.88828300	-1.19214700
H	-1.31426400	-2.35378000	0.43129900
C	1.99983700	0.15418000	0.19494100
H	1.84334900	1.14005100	-0.25759100
H	2.06226700	0.32202900	1.27848900
C	3.33395400	-0.41393000	-0.30364000
H	3.26856900	-0.59186100	-1.38389300
H	3.50185400	-1.39583000	0.15537900
C	4.52856500	0.49761400	-0.00886900
H	5.46345600	0.06145800	-0.37140400
H	4.40969000	1.47377700	-0.48949000
H	4.63815700	0.67079700	1.06634000

R38

C	2.41547900	-0.41330200	0.61314300
C	0.98612300	-0.75039600	1.02279100
C	-0.08326900	-0.08201700	0.13689100
C	0.17553200	1.43302500	0.05112900
C	1.59671700	1.74706600	-0.43826400
C	2.66301600	1.08225800	0.44110800
H	0.02043200	-0.49727400	-0.87441400
H	0.85870400	-1.83743200	1.01745400
H	0.85694800	-0.42243700	2.06188100
H	3.14314200	-0.85413400	1.29831500
H	-0.55203700	1.90364400	-0.61562000
H	1.76045100	2.82862600	-0.45848500
H	3.66563400	1.22438600	0.03071100
H	2.65894300	1.53261400	1.44113300
H	0.02182000	1.87824600	1.04453300
H	1.71072600	1.39567000	-1.46953000
O	2.63271500	-1.10801700	-0.67655800
O	3.89561900	-1.08534400	-1.04513600
C	-1.49163700	-0.43092300	0.64730300
H	-1.54815500	-1.51758300	0.79092700
H	-1.63189100	0.01285900	1.64272100
C	-2.64501200	-0.00077100	-0.26761400
H	-2.65553700	1.08995700	-0.37468900
H	-2.47739200	-0.40522700	-1.27430100
C	-4.01798700	-0.46096500	0.23748100
H	-4.18041900	-0.06773100	1.24844100
H	-4.01878100	-1.55348200	0.33336200
C	-5.17314400	-0.02749500	-0.66934000
H	-6.13573400	-0.37339600	-0.28297600
H	-5.22214800	1.06264400	-0.75410900
H	-5.05628800	-0.43193700	-1.67957800

TS38

C	0.79663300	-0.87881100	0.18571700
C	-0.15456300	-1.64489600	-0.75056800
C	-1.32632300	0.17366300	1.16300600
C	0.10931700	0.41186000	0.67522900
H	-0.42573600	-1.00086000	-1.59236300
H	0.94462500	-1.48736500	1.08952600
H	-2.08156400	0.93464700	0.38690500
H	-1.58261900	0.64064600	2.11325700
H	0.68610200	0.86041100	1.48970800
O	-3.34954500	-0.08679900	-0.65645100
O	-2.98901800	1.12735100	-0.51234100

C	-1.41711300	-2.09047000	-0.00702600
H	-1.17544400	-2.93540600	0.65702300
C	-1.99145800	-1.01616800	0.86050700
H	-2.87091000	-1.28022700	1.43817900
H	-2.18065500	-2.46518300	-0.69533700
O	0.17802800	1.30719600	-0.44810300
O	-0.33009300	2.60016900	-0.01974100
H	-1.05722000	2.71159300	-0.64893500
C	2.17562400	-0.64619900	-0.45669800
H	2.58424800	-1.62888500	-0.72456100
H	2.04903300	-0.09576900	-1.39383500
H	0.35413900	-2.51848000	-1.16807900
C	3.19085100	0.08938800	0.42566900
H	2.83120200	1.10264200	0.62943200
H	3.26892700	-0.41751100	1.39604500
C	4.57871600	0.17133700	-0.21719800
H	5.28364200	0.70733900	0.42386500
H	4.99006500	-0.82574800	-0.40344400
H	4.53707200	0.69659800	-1.17612800

P38

C	0.91171800	0.60644500	-0.56553000
C	2.22630300	-0.12687300	-0.68011000
C	2.39104100	-1.38730000	-0.27770000
C	1.29814000	-2.21360100	0.34403700
C	0.08690600	-1.36624300	0.75678500
C	-0.27667300	-0.34512800	-0.33512500
H	0.73633800	1.20097800	-1.47015800
H	0.99096200	-2.98973600	-0.37199400
H	1.69628700	-2.75739400	1.20836500
H	0.31759200	-0.82272300	1.67883300
H	-0.39182400	-0.89029000	-1.28289200
H	3.05452100	0.41950100	-1.11959300
H	3.35791800	-1.86752700	-0.40988800
O	0.90801000	1.52416500	0.55120300
O	1.88847600	2.55834300	0.27992700
H	2.59979400	2.29118500	0.87844400
H	-0.76292400	-2.01843800	0.97291400
C	-1.58766000	0.41589300	-0.06850100
H	-1.64519300	1.26580400	-0.75952800
H	-1.55843400	0.85163500	0.93528500
C	-2.85373300	-0.43499400	-0.23062300
H	-2.82988100	-1.28227000	0.46338400
H	-2.87187800	-0.86714800	-1.23900700
C	-4.13866600	0.36548500	0.00269900

H	-4.21719900	1.20052600	-0.70028900
H	-5.02719700	-0.25940200	-0.12232800
H	-4.16502400	0.78313200	1.01381000

R39

C	2.41547900	-0.41330200	0.61314300
C	0.98612300	-0.75039600	1.02279100
C	-0.08326900	-0.08201700	0.13689100
C	0.17553200	1.43302500	0.05112900
C	1.59671700	1.74706600	-0.43826400
C	2.66301600	1.08225800	0.44110800
H	0.02043200	-0.49727400	-0.87441400
H	0.85870400	-1.83743200	1.01745400
H	0.85694800	-0.42243700	2.06188100
H	3.14314200	-0.85413400	1.29831500
H	-0.55203700	1.90364400	-0.61562000
H	1.76045100	2.82862600	-0.45848500
H	3.66563400	1.22438600	0.03071100
H	2.65894300	1.53261400	1.44113300
H	0.02182000	1.87824600	1.04453300
H	1.71072600	1.39567000	-1.46953000
O	2.63271500	-1.10801700	-0.67655800
O	3.89561900	-1.08534400	-1.04513600
C	-1.49163700	-0.43092300	0.64730300
H	-1.54815500	-1.51758300	0.79092700
H	-1.63189100	0.01285900	1.64272100
C	-2.64501200	-0.00077100	-0.26761400
H	-2.65553700	1.08995700	-0.37468900
H	-2.47739200	-0.40522700	-1.27430100
C	-4.01798700	-0.46096500	0.23748100
H	-4.18041900	-0.06773100	1.24844100
H	-4.01878100	-1.55348200	0.33336200
C	-5.17314400	-0.02749500	-0.66934000
H	-6.13573400	-0.37339600	-0.28297600
H	-5.22214800	1.06264400	-0.75410900
H	-5.05628800	-0.43193700	-1.67957800

TS39

C	-0.21438400	1.54643300	-0.00223600
C	0.04606800	0.05971300	0.29441600
C	-2.70721200	0.95594200	0.02859000
C	-1.59146300	1.76957700	-0.64583600
H	-0.10904600	-0.50788700	-0.63346400
H	-0.15597600	2.10142200	0.94357600
H	0.56058300	1.95637700	-0.65494200

H	-3.10137100	0.08239700	-0.87346000
H	-3.65537600	1.47503100	0.17070300
H	-1.53472800	1.51620100	-1.71084100
H	-1.84953900	2.83188500	-0.60471700
O	-2.86667700	-1.76149400	-0.33049700
O	-3.26206300	-1.12204400	-1.35903100
C	-0.99453900	-0.42435400	1.31684100
H	-0.75212500	-0.00058900	2.30517300
C	-2.39546300	-0.01105200	0.98671200
H	-3.17436400	-0.35520600	1.65821000
C	1.47288200	-0.22416100	0.79230400
H	1.68726000	0.42480600	1.65253800
H	1.51062800	-1.25282800	1.17280200
H	-0.94751200	-1.51114400	1.43933100
C	2.57351300	-0.05546100	-0.26232700
H	2.58254700	0.97446900	-0.63777700
H	2.34378900	-0.69304300	-1.12587600
C	3.97215400	-0.40293700	0.26152300
H	4.20140500	0.23108700	1.12669400
H	3.97167300	-1.43467900	0.63336000
C	5.07030500	-0.24207200	-0.79342000
H	5.12086400	0.78857900	-1.15842700
H	6.05318500	-0.49915300	-0.38927300
H	4.88685700	-0.88919500	-1.65679200

P39

C	3.04341200	-0.91218400	-0.33665400
C	3.34282300	0.55720300	-0.19027900
C	2.41549500	1.46254300	0.11911600
C	0.95781700	1.13316600	0.32200500
C	0.58292800	-0.27459600	-0.18040300
C	1.66935400	-1.27827900	0.24102300
H	3.09378100	-1.19158200	-1.39862200
H	3.82713600	-1.49909700	0.15654300
H	0.70836700	1.22112100	1.39059600
H	0.34700900	1.89007100	-0.18241800
H	0.57348300	-0.24132700	-1.27959600
H	1.73095200	-1.29217900	1.33668300
H	1.38801300	-2.28970300	-0.07044800
H	4.37100000	0.87427700	-0.34516400
C	-0.80956300	-0.72333500	0.29071100
H	-0.97170300	-1.75624500	-0.04293000
H	-0.82046400	-0.75445500	1.38888000
H	2.70295000	2.50515300	0.23054200
C	-1.97688400	0.14001900	-0.20365000

H	-1.87109100	1.16458300	0.17146300
H	-1.93595700	0.21039900	-1.29875700
C	-3.34948900	-0.40090200	0.21431200
H	-3.38750900	-0.48083800	1.30753800
H	-3.46642100	-1.42220300	-0.16815300
C	-4.51574400	0.46272900	-0.27443700
H	-4.52354500	0.53515000	-1.36657600
H	-5.47848000	0.04827900	0.03711100
H	-4.44840400	1.48040300	0.12284500

R40

C	-2.68176500	0.44208900	0.49121000
C	-1.59823900	1.50898100	0.59299400
C	-0.27303500	1.07104300	-0.05027200
C	0.21902400	-0.28587100	0.48515300
C	-0.88234700	-1.34579500	0.29852600
C	-2.21211900	-0.93618700	0.94572000
H	-0.40570800	0.99665600	-1.13609300
H	-1.96008200	2.43657100	0.14009900
H	-1.44421600	1.71607100	1.65839300
H	-3.59224300	0.73873100	1.01671000
H	-0.55458700	-2.30285700	0.71687200
H	-2.99948800	-1.66105700	0.72523700
H	-2.10447400	-0.90302700	2.03653800
H	0.47613900	1.84856300	0.12101500
H	0.39983300	-0.17749100	1.56591600
H	-1.03890200	-1.51181200	-0.77391100
O	-3.06010900	0.39548800	-0.93931000
O	-4.13272700	-0.33854400	-1.14458500
C	1.53572700	-0.74103200	-0.16807400
H	1.74780100	-1.76654200	0.15994300
H	1.39242800	-0.79587800	-1.25555500
C	2.75840800	0.13225700	0.14075300
H	2.59504500	1.15329500	-0.22255000
H	2.88056400	0.21098300	1.22919800
C	4.05641700	-0.40643300	-0.47304400
H	3.93343700	-0.48832900	-1.55974900
H	4.23133900	-1.42640700	-0.10995900
C	5.27845400	0.46253700	-0.16258100
H	6.18575500	0.05241000	-0.61432900
H	5.14840700	1.47976300	-0.54517000
H	5.44849000	0.53527000	0.91624600

TS40

C	0.23463400	-0.23137700	-0.52080400

C	-0.32380900	0.99746400	0.21715100
C	-2.24791600	-0.84201100	-0.87315600
C	-0.83043300	-1.34402900	-0.55310900
H	-0.61912500	0.70568600	1.23246300
H	0.43257600	0.07116300	-1.55985600
H	-2.95098200	-1.05651400	0.21818700
H	-2.82651500	-1.46327600	-1.55725300
H	-0.82589000	-1.86114200	0.41493600
H	-0.54387400	-2.09666400	-1.29420700
O	-3.69067500	0.55340500	0.99980600
O	-3.64403000	-0.69134400	1.26681700
C	-1.53460900	1.57566500	-0.52345000
H	-1.19803300	2.06683600	-1.44976500
C	-2.52718500	0.52568700	-0.91419600
H	-3.42388500	0.86457300	-1.42104100
H	-2.02734700	2.35529200	0.06526700
C	1.55118100	-0.75629300	0.07598400
H	1.40414800	-0.94867400	1.14716200
H	1.77387000	-1.73076600	-0.37734300
H	0.43933100	1.77281400	0.32124500
C	2.76603900	0.15964200	-0.11781400
H	2.58578800	1.13113100	0.35695400
H	2.89694900	0.36328900	-1.18877000
C	4.06512100	-0.43107500	0.44341600
H	3.93364200	-0.64011900	1.51189900
H	4.25620000	-1.39999100	-0.03367800
C	5.27906100	0.48158600	0.24793100
H	6.18783600	0.03027100	0.65527800
H	5.45574000	0.68318400	-0.81331700
H	5.13471100	1.44486000	0.74714100

P40

C	3.04341200	-0.91218400	-0.33665400
C	3.34282300	0.55720300	-0.19027900
C	2.41549500	1.46254300	0.11911600
C	0.95781700	1.13316600	0.32200500
C	0.58292800	-0.27459600	-0.18040300
C	1.66935400	-1.27827900	0.24102300
H	3.09378100	-1.19158200	-1.39862200
H	3.82713600	-1.49909700	0.15654300
H	0.70836700	1.22112100	1.39059600
H	0.34700900	1.89007100	-0.18241800
H	0.57348300	-0.24132700	-1.27959600
H	1.73095200	-1.29217900	1.33668300
H	1.38801300	-2.28970300	-0.07044800

H	4.37100000	0.87427700	-0.34516400
C	-0.80956300	-0.72333500	0.29071100
H	-0.97170300	-1.75624500	-0.04293000
H	-0.82046400	-0.75445500	1.38888000
H	2.70295000	2.50515300	0.23054200
C	-1.97688400	0.14001900	-0.20365000
H	-1.87109100	1.16458300	0.17146300
H	-1.93595700	0.21039900	-1.29875700
C	-3.34948900	-0.40090200	0.21431200
H	-3.38750900	-0.48083800	1.30753800
H	-3.46642100	-1.42220300	-0.16815300
C	-4.51574400	0.46272900	-0.27443700
H	-4.52354500	0.53515000	-1.36657600
H	-5.47848000	0.04827900	0.03711100
H	-4.44840400	1.48040300	0.12284500

R41

C	1.59911800	1.90038000	-0.88356700
C	0.18098700	1.64622300	-0.35348900
C	-0.01202200	0.22111500	0.19847500
C	1.09268700	-0.12353200	1.21689600
C	2.51077400	0.19338700	0.75147500
C	2.66842200	1.59317800	0.17244300
H	-0.05024500	2.34815900	0.45666700
H	-0.54796600	1.82212800	-1.14690900
H	1.77061000	1.27849500	-1.76488000
H	1.69006000	2.94194500	-1.20509100
H	0.91699000	0.44761100	2.13443900
H	1.02839400	-1.17821700	1.48611300
H	3.22252600	0.00945500	1.56070100
H	2.59184300	2.30104900	1.00618900
H	3.67372900	1.70252300	-0.24403700
C	-1.39619000	0.00367700	0.83789600
H	-1.42120100	-1.02125800	1.22011500
H	-1.47878800	0.66590400	1.70844600
O	0.12885100	-0.59317600	-0.99252200
O	0.00015200	-1.99387100	-0.65581400
H	0.93234300	-2.27508000	-0.67205500
O	2.93606800	-0.72352200	-0.33688400
O	2.82178700	-1.99279000	-0.01530100
C	-2.59498300	0.21391900	-0.09374800
H	-2.65079500	1.26150300	-0.41192000
H	-2.44768100	-0.38030900	-1.00066100
C	-3.92552300	-0.17444200	0.56256300

H	-4.06298700	0.40697300	1.48291500
H	-3.87995500	-1.22569200	0.87069700
C	-5.13201900	0.03489900	-0.35676800
H	-6.06426100	-0.25739900	0.13430300
H	-5.22760200	1.08450500	-0.65248800
H	-5.03738200	-0.55824000	-1.27142500

TS41

C	0.13183500	1.49786900	-0.63288200
C	1.58012800	1.77359000	-1.05028400
C	1.12250100	-0.17410300	1.01848700
C	-0.03244200	0.12245800	0.04026100
H	1.91261700	0.99134200	-1.73793500
H	-0.20592100	2.26483100	0.07369600
H	-0.53133800	1.54173700	-1.49894600
H	1.77596800	-1.19844000	0.50162200
H	0.82105300	-0.65451800	1.94939000
O	3.63378200	-0.80223800	0.12070600
O	2.81922600	-1.77467300	-0.00683400
C	2.49352900	1.80636100	0.17647200
H	2.31120200	2.72765600	0.75247100
C	2.24058700	0.65864600	1.10067200
H	2.86189900	0.60386100	1.98824200
H	3.55028900	1.83524700	-0.10444600
H	1.64361600	2.72258000	-1.58943500
C	-1.37644900	-0.03279600	0.77515800
H	-1.40687100	-1.04772200	1.18457200
H	-1.37151300	0.65035700	1.63328600
O	0.00001100	-0.75835700	-1.11729900
O	-0.08431800	-2.14239400	-0.67771600
H	0.76161500	-2.47489900	-1.01046700
C	-2.62804400	0.20057900	-0.07725300
H	-2.66297500	1.24040800	-0.42285800
H	-2.56816800	-0.42438700	-0.97360200
C	-3.92381000	-0.11311400	0.68080500
H	-3.97312100	0.49970500	1.58956900
H	-3.89861600	-1.15601400	1.01840600
C	-5.18249100	0.12002600	-0.15945100
H	-6.08878900	-0.11827200	0.40378600
H	-5.25637400	1.16356500	-0.48113200
H	-5.17623700	-0.50283800	-1.05899000

P41

C	-0.49867000	0.17227900	0.06581500
C	-1.56692800	0.65728900	1.02616800
C	-2.78937500	0.13141500	1.11166100

C	-3.28426400	-0.98739400	0.23580500
C	-2.37448900	-1.20292000	-0.97838800
C	-0.89639500	-1.16802000	-0.57592400
H	-3.34757600	-1.90705000	0.83561400
H	-4.31070500	-0.77751100	-0.08534000
H	-2.55580000	-0.41262800	-1.71256200
H	-0.68404900	-1.96408900	0.14684300
H	-0.25958300	-1.34404000	-1.44573200
H	-1.27851200	1.46294400	1.69442300
H	-3.47801900	0.51291700	1.86216500
O	-0.41309900	1.05521600	-1.09544700
O	-0.15744500	2.41387800	-0.65638400
H	-1.05126000	2.78272600	-0.68711900
C	0.86418400	0.12244800	0.78104100
H	1.06861400	1.13002500	1.15676900
H	0.76098100	-0.52472000	1.66053600
H	-2.60924300	-2.15373400	-1.46561300
C	2.04480900	-0.33996500	-0.07969100
H	1.90393000	-1.38260500	-0.38796100
H	2.06884900	0.25614100	-0.99743600
C	3.38952100	-0.21614800	0.64698700
H	3.35695000	-0.79784100	1.57670900
H	3.54082900	0.82767300	0.94683000
C	4.57670700	-0.67931800	-0.20209000
H	5.52122700	-0.57280400	0.33862100
H	4.47388100	-1.73140700	-0.48636200
H	4.65323300	-0.09474700	-1.12404600

R42

C	1.59911800	1.90038000	-0.88356700
C	0.18098700	1.64622300	-0.35348900
C	-0.01202200	0.22111500	0.19847500
C	1.09268700	-0.12353200	1.21689600
C	2.51077400	0.19338700	0.75147500
C	2.66842200	1.59317800	0.17244300
H	-0.05024500	2.34815900	0.45666700
H	-0.54796600	1.82212800	-1.14690900
H	1.77061000	1.27849500	-1.76488000
H	1.69006000	2.94194500	-1.20509100
H	0.91699000	0.44761100	2.13443900
H	1.02839400	-1.17821700	1.48611300
H	3.22252600	0.00945500	1.56070100
H	2.59184300	2.30104900	1.00618900
H	3.67372900	1.70252300	-0.24403700

C	-1.39619000	0.00367700	0.83789600
H	-1.42120100	-1.02125800	1.22011500
H	-1.47878800	0.66590400	1.70844600
O	0.12885100	-0.59317600	-0.99252200
O	0.00015200	-1.99387100	-0.65581400
H	0.93234300	-2.27508000	-0.67205500
O	2.93606800	-0.72352200	-0.33688400
O	2.82178700	-1.99279000	-0.01530100
C	-2.59498300	0.21391900	-0.09374800
H	-2.65079500	1.26150300	-0.41192000
H	-2.44768100	-0.38030900	-1.00066100
C	-3.92552300	-0.17444200	0.56256300
H	-4.06298700	0.40697300	1.48291500
H	-3.87995500	-1.22569200	0.87069700
C	-5.13201900	0.03489900	-0.35676800
H	-6.06426100	-0.25739900	0.13430300
H	-5.22760200	1.08450500	-0.65248800
H	-5.03738200	-0.55824000	-1.27142500

TS42

C	-0.76144600	1.96145200	-0.39282000
C	0.04523400	0.66771700	-0.15664200
C	-2.78007300	0.77044900	0.63829100
C	-2.27892100	1.73190300	-0.44831100
H	-0.51662400	2.64681400	0.42546200
H	-0.42078300	2.43205400	-1.31883300
H	-3.25501200	-0.25960100	0.00743200
H	-3.69557400	1.06190200	1.15464400
H	-2.54739700	1.34937900	-1.43593500
H	-2.79023000	2.69261000	-0.33961900
O	-2.31391900	-1.93383500	0.34880400
O	-3.27659900	-1.53206600	-0.38493200
C	-0.38832100	0.04228900	1.17756800
H	0.06175100	0.62163500	1.99712000
C	-1.86884800	0.03493900	1.39803400
H	-2.21130300	-0.43215500	2.31508500
C	1.55357200	0.97651100	-0.22042900
H	1.74103200	1.83864200	0.43350900
H	1.75719500	1.31511200	-1.24217700
H	0.01585700	-0.96665800	1.27446100
O	-0.37705300	-0.15479700	-1.27080400
O	0.12279600	-1.50929800	-1.13840900
H	-0.67995100	-1.96471100	-0.83168100
C	2.54155000	-0.14015900	0.14525600
H	2.36363400	-0.48083100	1.17254000

H	2.37298200	-1.00445200	-0.49988900
C	4.00038100	0.31917000	0.02585700
H	4.16362000	1.19266200	0.66979100
H	4.18710200	0.65948600	-0.99984400
C	5.00632900	-0.77682800	0.38855100
H	6.03625500	-0.42340500	0.28853400
H	4.86983200	-1.11374400	1.42109100
H	4.88907300	-1.64943700	-0.26109200

P42

C	2.32436000	-1.47103100	-0.74311300
C	3.32808000	-0.59195900	-0.04853500
C	2.98158300	0.38125700	0.79188900
C	1.55429700	0.71868600	1.13725700
C	0.51107600	0.06279000	0.21478900
C	0.92862700	-1.38302900	-0.10738100
H	2.66667600	-2.51205400	-0.72548300
H	1.34449700	0.42396500	2.17370300
H	1.40098100	1.80177600	1.10873100
H	0.91787600	-1.93653600	0.83760400
H	0.18870100	-1.84531500	-0.76344500
H	4.37872400	-0.76750400	-0.26437200
C	-0.89524000	0.16600600	0.83318600
H	-1.05819900	1.21314000	1.10864000
H	-0.89414100	-0.40465900	1.76974700
H	3.75356400	0.97988500	1.26886600
H	2.26943500	-1.19136300	-1.80186200
O	0.53165100	0.67754700	-1.09907300
O	0.17735400	2.08447500	-0.99233800
H	1.01101300	2.48743400	-1.26931200
C	-2.05338500	-0.29630000	-0.05791900
H	-1.95768700	-1.36500800	-0.28216100
H	-1.99664300	0.22822600	-1.01637700
C	-3.42468800	-0.04731500	0.58203100
H	-3.47533400	-0.56106300	1.55021500
H	-3.53062300	1.02224600	0.79971100
C	-4.59086400	-0.50515200	-0.29849700
H	-5.55460500	-0.30870800	0.17949800
H	-4.58466300	0.01466600	-1.26146600
H	-4.53468300	-1.57902100	-0.50319000

R43

C	-0.65677300	-1.76666000	-0.64535600
C	0.35260500	-0.96286700	0.19776500
C	-0.26595400	0.37927500	0.63730800

C	-1.60676200	0.18940400	1.35696700
C	-2.59793900	-0.67059500	0.58172800
C	-1.99740700	-1.97537900	0.07391500
H	0.53776100	-1.51695000	1.12969400
H	-0.82890300	-1.24443600	-1.59024500
H	-0.22616000	-2.74108400	-0.89514100
H	-1.42276000	-0.29945800	2.31977600
H	-2.05347600	1.15891900	1.57603000
H	-3.49981100	-0.84654100	1.17400700
H	-1.86041700	-2.62834800	0.94379700
H	-2.71640800	-2.47330200	-0.58295600
O	-0.40604000	1.13999200	-0.57039400
O	-0.89784900	2.45835200	-0.24194100
H	-1.83436400	2.37179400	-0.49420400
O	-3.07870000	0.03706400	-0.63374800
O	-3.55888000	1.23322500	-0.37998100
C	1.69768100	-0.81005500	-0.53581000
H	2.04170500	-1.81483400	-0.81145200
H	1.53260200	-0.26953800	-1.47269400
H	0.41638900	0.91615400	1.30469100
C	2.80318300	-0.11092200	0.26380700
H	2.51553100	0.92661100	0.46655600
H	2.91720100	-0.59927500	1.24130100
C	4.15617000	-0.11296600	-0.45819000
H	4.45792200	-1.14846500	-0.65774600
H	4.03973500	0.36438100	-1.43831200
C	5.26120700	0.59838700	0.32754700
H	6.21119700	0.57978400	-0.21345300
H	5.00387600	1.64622100	0.51042400
H	5.42407300	0.12357600	1.30036000

TS43

C	-0.49855000	-0.03459300	0.67972700
C	0.66356100	0.96774800	0.79627500
C	1.37360300	-1.76197700	0.22008500
C	-0.05494800	-1.29209100	-0.10100900
H	-0.72033800	-0.33780100	1.71250400
H	2.07184400	-1.54124000	-0.85535600
H	1.51169400	-2.84244000	0.27533300
H	-0.14784500	-1.09318700	-1.17327700
O	3.55290800	-0.29392500	-0.62665500
O	3.02811800	-0.96105400	-1.57711600
C	1.83949300	0.35068500	1.55582000
H	1.55719800	0.25571200	2.61470100
C	2.20645600	-1.00666400	1.04783000

H	3.07911000	-1.47593000	1.48799800
H	2.69976300	1.02265500	1.53653200
H	0.33276900	1.87929600	1.30786500
O	1.00421600	1.29722900	-0.55475100
O	2.08720900	2.25927200	-0.54828400
H	2.82643000	1.68402500	-0.80850500
C	-1.76353400	0.61653500	0.09503600
H	-1.97311500	1.53090700	0.66416500
H	-1.55599900	0.93812300	-0.93063700
H	-0.74503600	-2.11047700	0.11877000
C	-3.01064600	-0.27544600	0.11958000
H	-2.84850000	-1.16613300	-0.49856100
H	-3.17973000	-0.63931700	1.14231000
C	-4.27248700	0.44337500	-0.37293200
H	-4.44896800	1.32877500	0.24961300
H	-4.09892500	0.81716500	-1.38899900
C	-5.51897700	-0.44578900	-0.36143800
H	-5.73740600	-0.80872100	0.64789200
H	-6.39947300	0.09659600	-0.71626800
H	-5.38691900	-1.32089200	-1.00545600

P43

C	-2.61512500	0.25045200	0.99031600
C	-2.74169800	1.51873800	0.18696900
C	-1.76130400	2.00258300	-0.57360200
C	-0.40983100	1.34993300	-0.70377200
C	-0.14323600	0.30121200	0.39442500
C	-1.38798200	-0.57942300	0.60321100
H	-2.56235000	0.48515300	2.06218500
H	-3.50691300	-0.37325000	0.87463000
H	-0.32542300	0.87932500	-1.69237700
H	-0.01607700	0.84167200	1.34253900
H	-1.18661800	-1.33582600	1.37027200
H	-3.68958400	2.04740700	0.24387600
H	-1.92528900	2.91370200	-1.14320600
O	-1.57321900	-1.26288500	-0.64644200
O	-2.57861000	-2.29467300	-0.44965900
H	-3.24320600	-2.00764300	-1.09005700
H	0.36693500	2.12085000	-0.67207700
C	1.12287300	-0.53695800	0.14898300
H	1.18503400	-1.30288300	0.93244000
H	1.01567200	-1.07931300	-0.79580500
C	2.42961700	0.26570100	0.14272200
H	2.42070300	0.99342900	-0.67704600
H	2.50389000	0.85050300	1.06964700

C	3.67604200	-0.61688200	0.00447200
H	3.69932300	-1.33926800	0.82931000
H	3.59619000	-1.20941500	-0.91478300
C	4.98355300	0.17988500	-0.01329900
H	5.10751100	0.75777200	0.90804000
H	5.85133600	-0.47815500	-0.11107400
H	5.00605000	0.88494900	-0.85024900

R44

C	-0.65677300	-1.76666000	-0.64535600
C	0.35260500	-0.96286700	0.19776500
C	-0.26595400	0.37927500	0.63730800
C	-1.60676200	0.18940400	1.35696700
C	-2.59793900	-0.67059500	0.58172800
C	-1.99740700	-1.97537900	0.07391500
H	0.53776100	-1.51695000	1.12969400
H	-0.82890300	-1.24443600	-1.59024500
H	-0.22616000	-2.74108400	-0.89514100
H	-1.42276000	-0.29945800	2.31977600
H	-2.05347600	1.15891900	1.57603000
H	-3.49981100	-0.84654100	1.17400700
H	-1.86041700	-2.62834800	0.94379700
H	-2.71640800	-2.47330200	-0.58295600
O	-0.40604000	1.13999200	-0.57039400
O	-0.89784900	2.45835200	-0.24194100
H	-1.83436400	2.37179400	-0.49420400
O	-3.07870000	0.03706400	-0.63374800
O	-3.55888000	1.23322500	-0.37998100
C	1.69768100	-0.81005500	-0.53581000
H	2.04170500	-1.81483400	-0.81145200
H	1.53260200	-0.26953800	-1.47269400
H	0.41638900	0.91615400	1.30469100
C	2.80318300	-0.11092200	0.26380700
H	2.51553100	0.92661100	0.46655600
H	2.91720100	-0.59927500	1.24130100
C	4.15617000	-0.11296600	-0.45819000
H	4.45792200	-1.14846500	-0.65774600
H	4.03973500	0.36438100	-1.43831200
C	5.26120700	0.59838700	0.32754700
H	6.21119700	0.57978400	-0.21345300
H	5.00387600	1.64622100	0.51042400
H	5.42407300	0.12357600	1.30036000

TS44

C	0.47606400	-0.21670900	0.47118500
C	-0.00038500	-1.25340300	-0.56272200
C	-1.98909700	-0.01412300	1.13724100
C	-0.68904700	0.73278400	0.81044900
H	-0.36722200	-0.72834700	-1.44957100
H	0.69555000	-0.74869200	1.40819500
H	-2.87537800	0.43808200	0.26258000
H	-2.50520500	0.30833300	2.04082200
H	-0.41494200	1.36606800	1.66141700
O	-3.54709800	-0.94815200	-0.90700600
O	-3.67527800	0.30951400	-0.74245200
C	-1.11121700	-2.13328400	0.02082300
H	-0.67443600	-2.85565200	0.72841500
C	-2.13649900	-1.35705700	0.78499600
H	-2.92419300	-1.93411500	1.25772600
H	-1.59669300	-2.73579200	-0.75284200
O	-0.80812300	1.58379300	-0.34241200
O	-1.79397700	2.60835100	-0.04135800
H	-2.43293500	2.43965800	-0.74878100
C	1.74767800	0.54343800	0.05303300
H	1.61236900	0.95253300	-0.95281400
H	1.86928800	1.41054700	0.71336700
H	0.82948400	-1.88603100	-0.88572200
C	3.02767600	-0.29991000	0.10737200
H	2.93839300	-1.16792500	-0.55682100
H	3.15446600	-0.70349200	1.12091900
C	4.28450500	0.48894800	-0.27933000
H	4.15782800	0.89341200	-1.29056900
H	4.38298400	1.35681200	0.38373300
C	5.56600500	-0.34700300	-0.22001500
H	6.44185600	0.24450900	-0.50003400
H	5.73792100	-0.73732100	0.78807000
H	5.51257600	-1.20272000	-0.90045800

P44

C	-1.41211700	-0.58255000	-0.56884200
C	-2.68454600	0.21954900	-0.69792900
C	-2.78753300	1.48601700	-0.29394200
C	-1.66060400	2.25184200	0.34418800
C	-0.50105900	1.34081600	0.76941500
C	-0.17812000	0.30442600	-0.32089400
H	-1.25685600	-1.18351100	-1.47291100
H	-1.30388200	3.01256600	-0.36537600
H	-2.04048200	2.81363100	1.20523400
H	-0.77162500	0.80819400	1.68698900

H	-0.02257900	0.84486400	-1.26554900
H	-3.53477800	-0.28113600	-1.14963100
H	-3.72585200	2.01708900	-0.43702300
O	-1.47103200	-1.50162300	0.54519400
O	-2.50115500	-2.48198500	0.25872000
H	-3.20543100	-2.17812300	0.84799000
H	0.37919500	1.94680900	0.99785400
C	1.08739200	-0.52561400	-0.04006000
H	1.10839200	-1.37474700	-0.73392600
H	1.02079500	-0.96246300	0.96131500
C	2.39783200	0.25861100	-0.18202100
H	2.41184100	1.10312000	0.51724800
H	2.45289000	0.69534300	-1.18822800
C	3.64358400	-0.60218200	0.06017300
H	3.63965800	-1.44625500	-0.63992500
H	3.58761700	-1.04093900	1.06353800
C	4.95472400	0.17544800	-0.08502700
H	5.00421400	1.00468600	0.62780200
H	5.82194000	-0.46640600	0.09273300
H	5.05513100	0.59797000	-1.08980600
